# Evolutionary conserved circular MEF2A RNAs regulate myogenic differentiation and skeletal muscle development

**DOI:** 10.1371/journal.pgen.1010923

**Published:** 2023-09-07

**Authors:** Xiaoxu Shen, Xiyu Zhao, Haorong He, Jing Zhao, Yuanhang Wei, Yuqi Chen, Shunshun Han, Yifeng Zhu, Yao Zhang, Qing Zhu, Huadong Yin

**Affiliations:** 1 Key Laboratory of Livestock and Poultry Multi-omics, Ministry of Agriculture and Rural Affairs, College of Animal Science and Technology, Sichuan Agricultural University, Chengdu, Sichuan, China; 2 Farm Animal Genetic Resources Exploration and Innovation Key Laboratory of Sichuan Province, Sichuan Agricultural University, Chengdu, Sichuan, China; 3 Institute of Animal Nutrition, Key Laboratory for Animal Disease-Resistance Nutrition of China, Ministry of Education, Sichuan Agricultural University, Chengdu, Sichuan, China; The Jackson Laboratory, UNITED STATES

## Abstract

Circular RNAs (circRNAs) have been recognized as critical regulators of skeletal muscle development. Myocyte enhancer factor 2A (MEF2A) is an evolutionarily conserved transcriptional factor that regulates myogenesis. However, it remains unclear whether MEF2A produces functional circRNAs. In this study, we identified two evolutionarily conserved circular MEF2A RNAs (circMEF2As), namely circMEF2A1 and circMEF2A2, in chicken and mouse muscle stem cells. Our findings revealed that circMEF2A1 promotes myogenesis by regulating the miR-30a-3p/PPP3CA/NFATC1 axis, whereas circMEF2A2 facilitates myogenic differentiation by targeting the miR-148a-5p/SLIT3/ROBO2/β-catenin signaling pathway. Furthermore, in vivo experiments demonstrated that circMEF2As both promote skeletal muscle growth. We also discovered that the linear MEF2A mRNA-derived MEF2A protein binds to its own promoter region, accelerating the transcription of MEF2A and upregulating the expression of both linear MEF2A and circMEF2As, forming a MEF2A autoregulated positive feedback loop. Moreover, circMEF2As positively regulate the expression of linear MEF2A by adsorbing miR-30a-3p and miR-148a-5p, which directly contribute to the MEF2A autoregulated feedback loop. Importantly, we found that mouse circMEF2As are essential for the myogenic differentiation of C2C12 cells. Collectively, our results demonstrated the evolution, function, and underlying mechanisms of circMEF2As in animal myogenesis, which may provide novel insight for both the farm animal meat industry and human medicine.

## Author summary

Skeletal muscles play important motor and metabolic functions in animals. The development of skeletal muscles is a complex biological process regulated by numerous genes, including MEF2A, a well-known muscle growth regulator. MEF2A messenger RNA-derived protein is a transcription factor that typically controls the expression of myogenic genes. In our study, we uncovered a non-canonical role of the MEF2A gene in skeletal muscle development, wherein it generates two non-coding circRNAs. These circRNAs regulate skeletal muscle myogenesis independently of the MEF2A protein. Circular RNAs have a different structure from linear messenger RNAs and were previously considered an erroneous product of genome transcription. However, our study not only highlights the potential role of MEF2A-derived circular RNAs in skeletal muscle development, but also reveals their evolutionary conservation and expression in multiple species, which proves that circRNAs are not errors to the organisms. In summary, our study demonstrated the multiple roles of the MEF2A gene in skeletal muscle development and provides strong evidence that circRNAs are important for animal physiological regulation.

## 1 Introduction

Skeletal muscle is an essential tissue that plays a pivotal role in supporting both the motor and metabolic functions of the organism [1]. In addition, the skeletal muscle of farm animals serves as the primary protein source for human consumption [2]. During the embryonic stage, multinucleated muscle fibers, comprising the skeletal muscle, are formed through the proliferation and differentiation of muscle progenitor cells [3]. Postnatally, the muscle progenitor cells transition into a quiescent state as skeletal muscle satellite cells (also known as skeletal muscle stem cells, SMSCs), retaining their proliferative and differentiative potential [4]. Upon muscle injury, satellite cells become activated and serve as repair cells or can differentiate and fuse with existing muscle fibers, thus increasing their thickness [5]. The precise orchestration of a large number of genes governs this series of biological processes, and deviations in gene expression can result in various pathological conditions of muscles, including sarcopenia, muscular dystrophy, and muscle metabolism disorders [6].

The family of evolutionarily conserved muscle regulatory factors (MRFs) is the foremost determinant of myogenesis in most animal species. The molecular mechanisms underlying the regulation of myogenic differentiation by MRFs have been extensively studied [7]. MEF2, another conserved gene family containing MADS-box domains, is primarily involved in skeletal muscle formation [8]. Among the MEF2 family members, MEF2A is crucial for skeletal muscle cell differentiation, and its absence impairs this process [9]. However, MEF2B, MEF2C, or MEF2D have independent but partially overlapping functions in myogenic differentiation [10], indicating that MEF2A is an indispensable and the most important member of the MEF2 family for myogenesis. Furthermore, non-coding genes have also been shown to play significant roles in the regulatory network of skeletal muscle development [11].

CircRNAs are a type of natural RNA molecule, which is formed by the back-splicing of RNA precursors and possesses a unique circular structure with a 5’ head to 3’ tail connection [12]. Initially, circRNAs were thought to be non-coding RNA molecules, and were considered by some to be the result of splicing errors [13]. However, recent research has demonstrated that circRNAs are expressed abundantly, dynamically, and conserved across various animal tissues and cells, and participate in many biological processes, including myogenesis [14–17]. For instance, circSVIL has been found to promote chicken myoblast proliferation and differentiation by sponging miR-203 [18], and regulate bovine myoblast development by interacting with Signal transducer and activator of transcription 1 (STAT1) and inhibiting STAT1 phosphorylation [19]. Similarly, circFGFR2 promotes chicken myogenesis by targeting miR-133a-5p and miR-29b-1-5p [20], and facilitates skeletal muscle development and regeneration in mice and pigs via a feedback loop [21]. Overall, conserved circRNAs are important regulatory elements in the complex network controlling skeletal muscle development.

The MRF and MEF2 families are known to play essential roles in skeletal muscle formation. However, the potential functional role of circRNAs produced by these genes remains uncertain. By conducting an extensive search of databases and prior sequencing data, we have identified two evolutionarily conserved circRNAs generated by the MEF2A gene. Therefore, the primary objective of this study is to investigate the functional and molecular mechanisms underlying circMEF2As in the context of skeletal muscle development.

## 2 Results

### 2.1 Identification of the evolutionarily conserved circMEF2As

**Fig 1:**
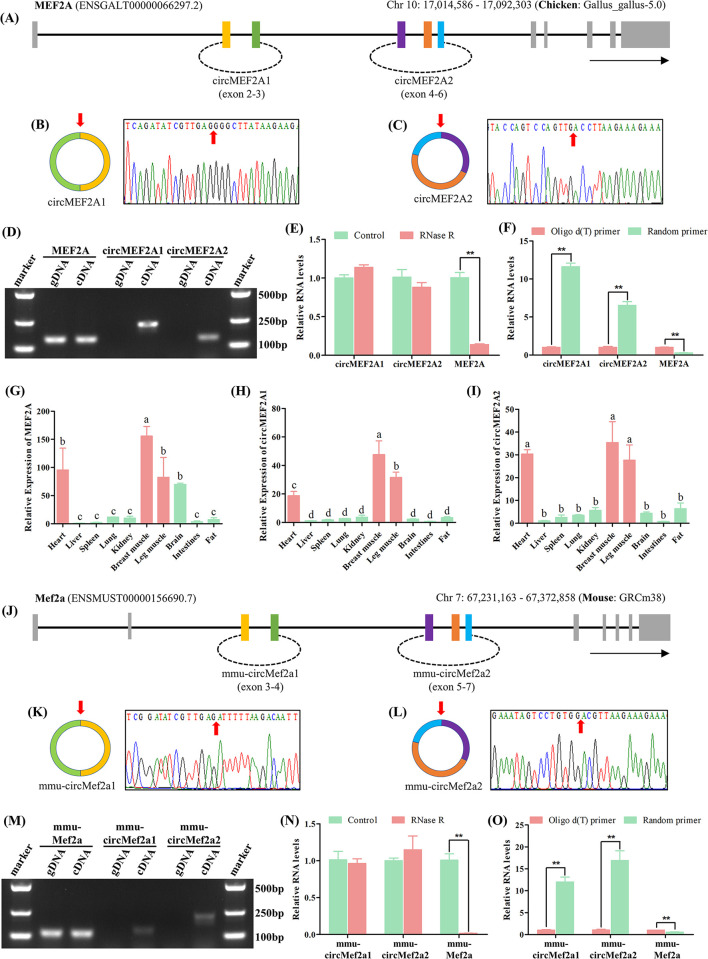
Identification of the evolutionarily conserved circMEF2As. (A) Origin information of MEF2A transcript from the chicken genome, circMEF2A1 and circMEF2A2 derived from the 2–3 and 4–6 exons of MEF2A transcript respectively. (B, C) The back-splicing junctions of circMEF2A1 and circMEF2A2 were amplified by divergent primers and sequenced by Sanger sequencing. (D) RT-PCR analysis of circMEF2A1, circMEF2A2, and linear MEF2A on the cDNA and genomic DNA samples extracted from chicken SMSCs; DNA marker: DL2000. (E) qRT-PCR analysis of circMEF2A1, circMEF2A2, and linear MEF2A on the cDNA samples generated from the RNase R treated and non-treated chicken SMSCs’ total RNAs, n = 3. (F) qRT-PCR analysis of circMEF2A1, circMEF2A2, and linear MEF2A on the cDNA samples generated by random primers and oligo d(T) primers from chicken SMSCs’ total RNAs, n = 3. (G-I) qRT-PCR analysis of circMEF2A1, circMEF2A2, and linear MEF2A on the cDNA samples generated from the different tissues of 7-day-old Tianfu chicks, n = 3. (J) Origin information of Mef2a transcript from the mouse genome, mmu-circMef2a1 and mmu-circMef2a2 derived from the 3–4 and 5–7 exons of mmu-Mef2a transcript respectively. (K, L) The back-splicing junctions of mmu-circMef2a1 and mmu-circMef2a2 were amplified by divergent primers and sequenced by Sanger sequencing. (M) RT-PCR analysis of mmu-circMef2a1, mmu-circMef2a2, and linear mmu-Mef2a on the cDNA and genomic DNA samples extracted from mouse C2C12 cells; DNA marker: DL2000. (N) qRT-PCR analysis of mmu-circMef2a1, mmu-circMef2a2, and linear mmu-Mef2a on the cDNA samples generated from the RNase R treated and non-treated mouse C2C12 cells’ total RNAs, n = 3. (O) qRT-PCR analysis of mmu-circMef2a1, mmu-circMef2a2, and linear mmu-Mef2a on the cDNA samples generated by random primers and oligo d(T) primers from mouse C2C12 cells’ total RNAs, n = 3. Data were displayed as mean ± SEM, independent sample *t*-test (E, F, N, and O), and one-way ANOVA analysis (G-I) were used to analyze the statistical differences between each dataset, **P < 0.01, *P < 0.05, and ^a, b, c^ *P* < 0.05.

To investigate the presence and function of circRNAs derived from the MRFs and MEF2s families, we employed the chicken as a research model and scanned the circRNAs expressed in chicken tissues using the circAtlas 2.0 database. Our findings indicated that MRFs exhibit a low level of circRNA production, whereas MEF2s, especially MEF2A, produce a substantially higher number of circRNAs (S1A Fig). We therefore focused our attention on exploring the expression profiles of circMEF2As in chicken skeletal muscle tissues. To this end, we analyzed three circRNA sequencing datasets (S1B–S1D Fig) [22–24], which revealed that the two most highly expressed circMEF2As are derived from exons 2–3 and 4–6 of the linear MEF2A transcript, respectively, we named these circRNAs circMEF2A1 and circMEF2A2 (Fig 1A). Subsequently, we identified the back-splicing sites of these circMEF2As using divergent primer amplification (Fig 1B–1D) and demonstrated that circMEF2As exhibit higher resistance to RNase R digestion compared to linear mRNAs (Fig 1E). Furthermore, our in vitro reverse transcription system revealed that random primers are more effective than oligo d(T) primers in reverse transcribing circMEF2As, while the opposite is true for linear mRNAs (Fig 1F). These results indicate that circMEF2As exist in a circular form without a poly(A) tail and are truly present in chicken tissues. Finally, we found that circMEF2As exhibit stronger muscle specificity compared to linear MEF2A mRNA based on tissue-distribution analysis (Fig 1G–1I).

In addition, MEF2A is a gene that has been conserved throughout evolution [25]. In order to assess the conservation of circMEF2As across different species, we conducted a comprehensive search for circRNAs expressed in humans, macaques, mice, rats, and pigs using the circAtlas 2.0 database, our analysis revealed potential circMEF2As in these species, which exhibit similar exon numbers and positions, as well as high sequence similarities exceeding 70% and 80%, respectively (S2A and S2B Fig). Moreover, we performed an evolutionary analysis that demonstrated remarkable similarity in the evolutionary patterns of circMEF2As and linear MEF2A mRNA across species, consistent with the known evolutionary history and speciation of these organisms (S2C–S2F Fig). To further validate our findings, we utilized C2C12 cells to identify mouse circMEF2As, confirming the existence of back-splicing sites for circMEF2A1 and circMEF2A2 by Sanger sequencing, and verifying their circular form through RNase R digestion and in vitro reverse transcription assays (Fig 1J–1O). Collectively, our study highlights the existence and evolutionary conservation of circMEF2As in animal skeletal muscle cells.

### 2.2 CircMEF2A1 promotes myogenesis in vitro and in vivo

**Fig 2:**
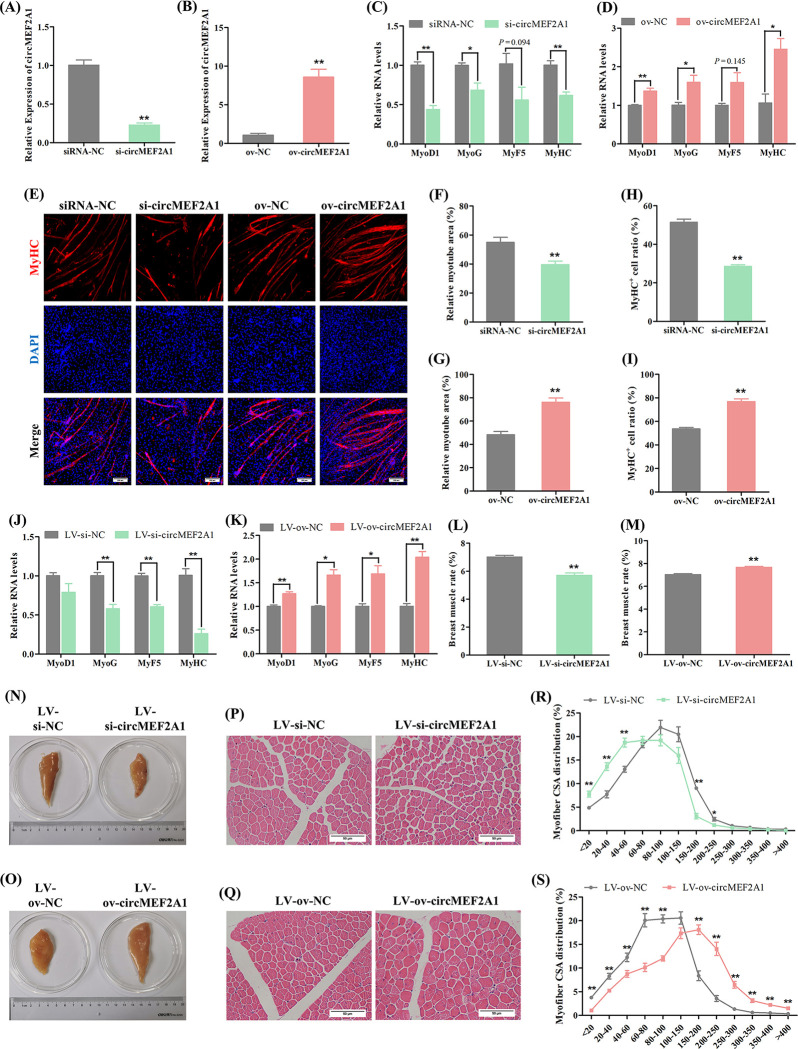
CircMEF2A1 promotes myogenesis in vitro and in vivo. (A, B) qRT-PCR analysis of circMEF2A1 in the cDNA samples generated from circMEF2A1 siRNA (si-circMEF2A1), negative control siRNA (siRNA-NC), overexpression vector (ov-circMEF2A1), and negative control vector (ov-NC) transfected SMSCs, n = 3. (C, D) qRT-PCR analysis of myogenic genes including MyoD1, MyoG, MyF5, and MyHC in the cDNA samples generated from si-circMEF2A1, siRNA-NC, ov-circMEF2A1, and ov-NC transfected SMSCs, n = 3. (E) Immunofluorescence of MyHC in si-circMEF2A1, siRNA-NC, ov-circMEF2A1, and ov-NC transfected SMSCs. Scale bars: 200 μm. (F, G) The relative myotube area of si-circMEF2A1, siRNA-NC, ov-circMEF2A1, and ov-NC transfected SMSCs was calculated by Image pro plus software, n = 9. (H, I) The proportion of MyHC^+^ cells of si-circMEF2A1, siRNA-NC, ov-circMEF2A1, and ov-NC transfected SMSCs was calculated by Image pro plus software, n = 9. (J, K) qRT-PCR analysis of myogenic genes in the cDNA samples generated from lentivirus packaged circMEF2A1 shRNA (LV-si-circMEF2A1), negative control shRNA (LV-si-NC), overexpression vector (LV-ov-circMEF2A1), and negative control vector (LV-ov-NC) infected breast muscles of Tianfu chicks, n = 3. (L, M) The breast muscle rate of the LV-si-circMEF2A1, LV-si-NC, LV-ov-circMEF2A1, and LV-ov-NC infected Tianfu chicks, n = 6. (N, O) Representative photographs of the unilateral breast muscles of the LV-si-circMEF2A1, LV-si-NC, LV-ov-circMEF2A1, and LV-ov-NC infected Tianfu chicks. (P, Q) Hematoxylin and eosin (H&E) staining of the cross-section of LV-si-circMEF2A1, LV-si-NC, LV-ov-circMEF2A1, and LV-ov-NC infected chicks’ breast muscle. Scale bars: 200 μm. (R, S) The myofiber cross-sectional area of LV-si-circMEF2A1, LV-si-NC, LV-ov-circMEF2A1, and LV-ov-NC infected chicks’ breast muscles was calculated by Image J software, n = 9. Data were displayed as mean ± SEM, independent sample *t*-test was used to analyze the statistical differences between each dataset, **P < 0.01 and *P < 0.05.

To investigate the involvement of circMEF2As in myogenesis, we conducted an analysis of the potential function of circMEF2A1 in the myogenic differentiation of SMSCs. The expression of circMEF2A1 in SMSCs was regulated using an overexpression vector (ov-circMEF2A1) and an effective siRNA (si-circMEF2A1) (Figs 2A, 2B and S3A). Our results demonstrated that the knockdown of circMEF2A1 during SMSC differentiation led to a decrease in the expression of myogenic differentiation marker genes such as Myogenin (MyoG), Myogenic differentiation 1 (MyoD1), Myogenic factor 5 (MyF5), and Myosin heavy chain (MyHC) (Fig 2C). Conversely, overexpression of circMEF2A1 resulted in increased expression of these marker genes (Fig 2D). Furthermore, the knockdown of circMEF2A1 resulted in a significant reduction in the relative myotube area and MyHC positive (MyHC^+^) cells, whereas circMEF2A1 overexpression markedly induced the formation and fusion of myotubes in SMSCs (Fig 2E–2I). Collectively, these findings suggest that circMEF2A1 has a positive role in myogenesis.

Subsequent lentivirus-mediated analyses were conducted to confirm the role of circMEF2A1 in regulating skeletal muscle development in vivo. Lentiviruses that specifically target circMEF2A1 were injected into the breast muscle, resulting in effective regulation of circMEF2A1 expression in vivo (S4A and S4B Fig). Compared to the control group, the expression of myogenic differentiation marker genes was downregulated by knockdown of circMEF2A1 (LV-si-circMEF2A1) (Fig 2J), but upregulated by overexpression of circMEF2A1 (LV-ov-circMEF2A1) (Fig 2K). Furthermore, knockdown of circMEF2A1 significantly reduced the breast muscle mass, breast muscle ratio to body weight, and cross-sectional area of breast muscle fibers, while overexpression of circMEF2A1 had the opposite effect (Figs 2L–2S and S4C–S4F). Taken together, these results strongly suggest that circMEF2A1 promotes skeletal muscle development.

### 2.3 CircMEF2A1 activates PPP3CA/NFATC1 signaling to regulate myogenesis via targeting miR-30a-3p

**Fig 3:**
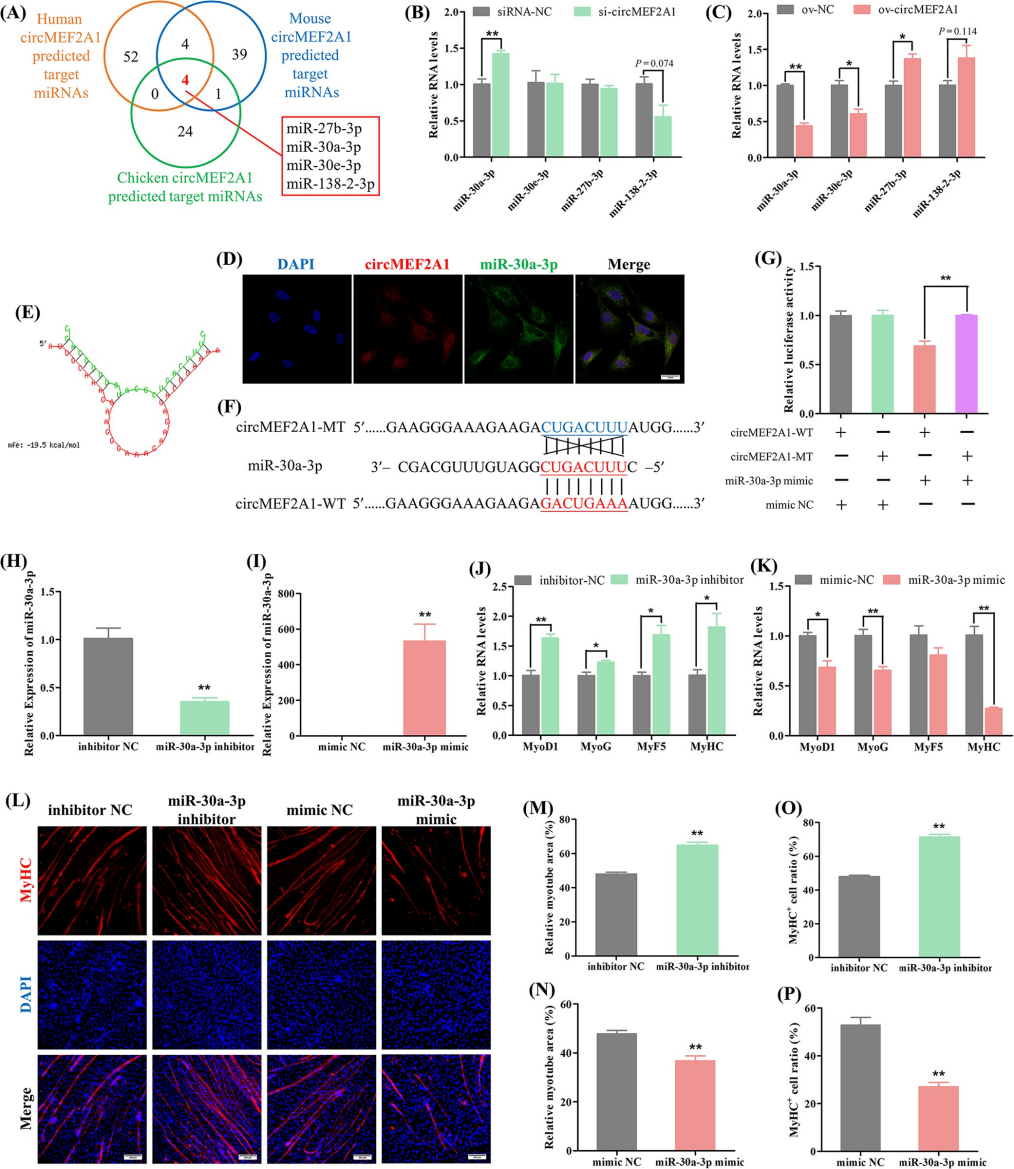
CircMEF2A1 targets and regulates miR-30a-3p. (A) Venn analysis of human, mouse, and chicken circMEF2A1 targeted miRNAs which predicted by RNAhybrid software, four co-targeted miRNAs including miR-30a-3p, miR-30e-3p, miR-27b-3p, miR-138-2-3p were observed. (B, C) qRT-PCR analysis of these four co-targeted miRNAs in the cDNA samples generated from si-circMEF2A1, siRNA-NC, ov-circMEF2A1, and ov-NC transfected SMSCs, n = 3. (D) RNA FISH analysis revealed the subcellular localization of circMEF2A1 and miR-30a-3p in normal growing SMSCs. Scale bars: 20 μm. (E) The image of circMEF2A1 and miR-30a-3p hybridization was predicted by RNAhybrid software. (F) Partial sequence of circMEF2A1 containing wild-type (circMEF2A1-WT) and mutant-type (circMEF2A1-MT) of miR-30a-3p response element were subcloned into the dual-luciferase reporter vector. (G) Dual-luciferase report analysis of circMEF2A1-WT and circMEF2A1-MT in DF-1 cells which co-transfected with miR-30a-3p mimic or negative control mimic (mimic NC), n = 3. (H, I) qRT-PCR analysis of miR-30a-3p in cDNA samples generated from miR-30a-3p inhibitor, negative control inhibitor (inhibitor NC), miR-30a-3p mimic and mimic NC transfected SMSCs, n = 3. (J, K) qRT-PCR analysis of myogenic genes in the cDNA samples generated from miR-30a-3p inhibitor, inhibitor NC, miR-30a-3p mimic and mimic NC transfected SMSCs, n = 3. (L) Immunofluorescence of MyHC in miR-30a-3p inhibitor, inhibitor NC, miR-30a-3p mimic and mimic NC transfected SMSCs. Scale bars: 200 μm. (M, N) The relative myotube area of miR-30a-3p inhibitor, inhibitor NC, miR-30a-3p mimic, and mimic NC transfected SMSCs was calculated by Image pro plus software, n = 9. (O, P) The proportion of MyHC^+^ cells of miR-30a-3p inhibitor, inhibitor NC, miR-30a-3p mimic, and mimic NC transfected SMSCs was calculated by Image pro plus software, n = 9. Data were displayed as mean ± SEM, independent sample *t*-test was used to analyze the statistical differences between each dataset, **P < 0.01 and *P < 0.05.

To elucidate the molecular mechanism underlying the regulation of muscle development by circMEF2A1, we investigated its subcellular localization and results indicate that circMEF2A1 is primarily expressed in the cytoplasm (S5A Fig), and the RNA binding protein immunoprecipitation-quantitative PCR (RIP-qPCR) results showed circMEF2A1 is highly enriched in the pull-down products of the Argonaute RISC catalytic component 2 (AGO2) protein (S5B Fig), suggesting its potential role in competing endogenous RNA (ceRNA) mechanism which binding to miRNAs. Due to the conservation of circMEF2A1, we predicted the target miRNAs of human, mouse, and chicken circMEF2A1 and identified four miRNAs that are potentially co-targeted across these three species, namely miR-27b-3p, miR-30a-3p, miR-30e-3p, and miR-138-2-3p (Figs 3A and S6A). Further investigation revealed that circMEF2A1 most significantly negatively regulates miR-30a-3p (Fig 3B and 3C). RNA-FISH results showed that they have the akin subcellular localization, which confirmed the physical basis of the interaction between circMEF2A1 and miR-30a-3p (Fig 3D). Moreover, the dual-luciferase reporter assay demonstrated that miR-30a-3p can bind to the response element on circMEF2A1, confirming that circMEF2A1 targets miR-30a-3p (Fig 3E–3G). Together, our findings shed light on the regulatory role of circMEF2A1 in muscle development through its interaction with miRNAs.

To investigate the functional significance of miR-30a-3p in myogenesis, we employed a specific miRNA inhibitor and mimic to manipulate the expression of miR-30a-3p in SMSCs (Fig 3H and 3I). Our results demonstrate that the interference of miR-30a-3p significantly upregulated the expression of muscle cell differentiation marker genes as well as increased the relative myotube area and the percentage of MyHC^+^ cells, while overexpression of miR-30a-3p resulted in a significant downregulation of myogenic genes as well as a reduction in the relative myotube area and the percentage of MyHC^+^ cells (Fig 3J–3P). These findings suggest that miR-30a-3p functions as a negative regulator of skeletal muscle development.

**Fig 4:**
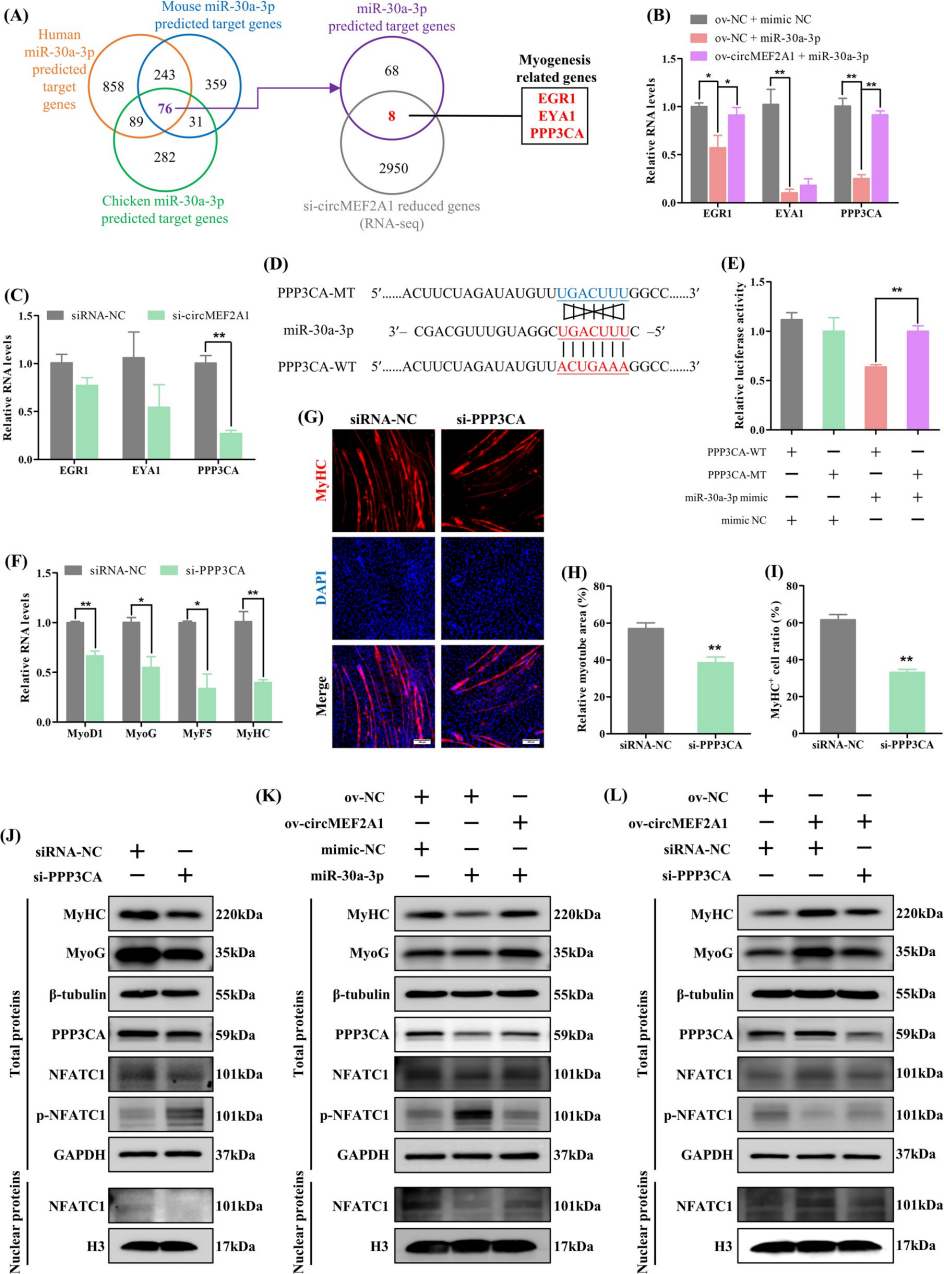
CircMEF2A1 activates PPP3CA/NFATC1 signaling to regulate myogenesis via targeting miR-30a-3p. (A) Venn analysis revealed human, mouse, and chicken miR-30a-3p was predicted co-targets 76 genes, and only 8 genes were found reduced in the circMEF2A1 knockdown SMSCs among these 76 genes, further 3 myogenesis relative genes were identified among these 8 genes. (B) qRT-PCR analysis of these 3 myogenesis relative genes in the cDNA samples generated from miR-30a-3p mimic or mimic NC and ov-circMEF2A1 or ov-NC co-transfected SMSCs, n = 3. (C) qRT-PCR analysis of these 3 myogenesis-relative genes in the cDNA samples generated from si-circMEF2A1 and siRNA-NC transfected SMSCs, n = 3. (D) Partial sequence of PPP3CA 3’UTR containing wild-type (PPP3CA-WT) and mutant-type (PPP3CA-MT) of miR-30a-3p response element was subcloned into the dual-luciferase reporter vector. (E) Dual-luciferase report analysis of PPP3CA-WT and PPP3CA-MT in DF-1 cells which co-transfected with miR-30a-3p mimic or mimic NC, n = 3. (F) qRT-PCR analysis of myogenic genes in the cDNA samples generated from PPP3CA siRNA (si-PPP3CA) and siRNA-NC transfected SMSCs, n = 3. (G) Immunofluorescence of MyHC in si-PPP3CA and siRNA-NC transfected SMSCs. Scale bars: 200 μm. (H) The relative myotube area of si-PPP3CA and siRNA-NC transfected SMSCs was calculated by Image pro plus software, n = 9. (I) The proportion of MyHC^+^ cells of si-PPP3CA and siRNA-NC transfected SMSCs was calculated by Image pro plus software, n = 9. (J) Western blot analysis of myogenic proteins including MyoG and MyHC, and circMEF2A1/miR-30a-3p target proteins including PPP3CA, NFATC1 and phosphorylated NFATC1 (p-NFATC1), and housekeeper proteins including β-tubulin, GAPDH, and H3, in total proteins or nuclear proteins extracted from si-PPP3CA and siRNA-NC transfected SMSCs. (K) Western blot analysis of myogenic proteins, circMEF2A1/miR-30a-3p target proteins, and housekeeper proteins in total proteins or nuclear proteins extracted from miR-30a-3p mimic or mimic NC and ov-circMEF2A1 or ov-NC co-transfected SMSCs. (L) Western blot analysis of myogenic proteins, circMEF2A1/miR-30a-3p target proteins, and housekeeper proteins in total proteins or nuclear proteins extracted from si-PPP3CA or siRNA-NC and ov-circMEF2A1 or ov-NC co-transfected SMSCs. Data were displayed as mean ± SEM, independent sample *t*-test was used to analyze the statistical differences between each dataset, **P < 0.01 and *P < 0.05.

Subsequently, we conducted a bioinformatic analysis to predict the target genes of miR-30a-3p, which identified 76 conserved target genes (Fig 4A). However, this number was too large to effectively screen for functionally relevant targets. To further investigate the regulation of upstream circMEF2A1, we performed transcriptome analysis on cells following circMEF2A1 knockdown, which revealed 2958 downregulated genes (S7 Fig). Venn analysis identified 8 potential target genes of miR-30a-3p that were also regulated by circMEF2A1. Among these genes, Early growth response 1 (EGR1) [26], EYA transcriptional coactivator and phosphatase 1 (EYA1) [27], and Protein phosphatase 3 catalytic subunit alpha (PPP3CA) [28] were of particular interest due to their known involvement in the regulation of skeletal muscle development (Figs 4A, S6B and S6C). Further investigation showed that PPP3CA was both regulated by circMEF2A1 and miR-30a-3p (Fig 4B and 4C). The dual-luciferase reporter assay confirmed that miR-30a-3p directly targets the 3’UTR of PPP3CA (Fig 4D and 4E). Moreover, specific siRNA-mediated knockdown of PPP3CA in SMSCs resulted in decreased expression of myogenic differentiation marker genes (Figs 4F and S3B), as well as a reduction in relative myotube area and the percentage of MyHC^+^ cells (Fig 4G–4I). These findings demonstrate that circMEF2A1 regulates PPP3CA by targeting miR-30a-3p, thereby controlling myogenesis.

PPP3CA, also known as calcineurin A, is a member of the calcineurin family, and has been identified as a regulator of myogenic differentiation through activation of the calcineurin/NFAT signaling pathway. To further investigate this, we conducted western blot assays and observed that PPP3CA knockdown led to a reduction in the protein levels of myogenic genes, as well as an increase in the phosphorylation of Nuclear factor of activated T cells 1 (NFATC1), thereby preventing active NFATC1 (non-phosphorylation) from entering the nucleus and executing the transcription process (Fig 4J). Similarly, overexpression of miR-30a-3p inhibited the activation of the calcineurin/NFAT signaling pathway and myogenic differentiation, which could be partially rescued by the addition of circMEF2A1 (Fig 4K). Additionally, circMEF2A1 was found to promote myogenic differentiation by stimulating the activation of the calcineurin/NFAT signaling pathway, but this effect was blocked in the absence of PPP3CA (Fig 4L). Taken together, our findings suggest the existence of a circMEF2A1/miR-30a-3p/PPP3CA/NFATC1 axis in regulating skeletal muscle myogenesis.

### 2.4 CircMEF2A2 promotes myogenesis in vitro and in vivo

**Fig 5:**
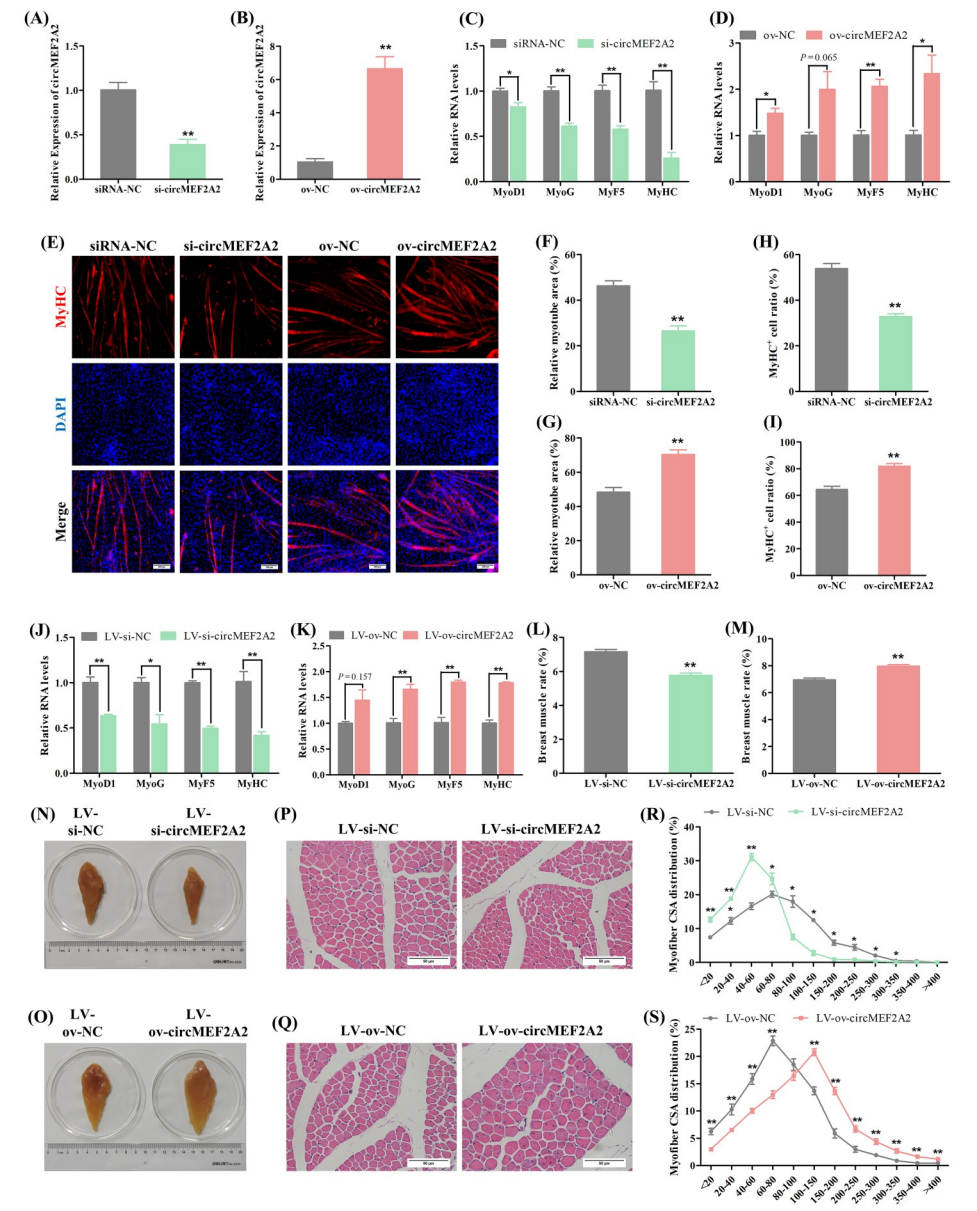
CircMEF2A2 promotes myogenesis in vitro and in vivo. (A, B) qRT-PCR analysis of circMEF2A2 in the cDNA samples generated from circMEF2A2 siRNA (si-circMEF2A2), siRNA-NC, overexpression vector (ov-circMEF2A2), and ov-NC transfected SMSCs, n = 3. (C, D) qRT-PCR analysis of myogenic genes in the cDNA samples generated from si-circMEF2A2, siRNA-NC, ov-circMEF2A2, and ov-NC transfected SMSCs, n = 3. (E) Immunofluorescence of MyHC in si-circMEF2A2, siRNA-NC, ov-circMEF2A2, and ov-NC transfected SMSCs. Scale bars: 200 μm. (F, G) The relative myotube area of si-circMEF2A2, siRNA-NC, ov-circMEF2A2, and ov-NC transfected SMSCs was calculated by Image pro plus software, n = 9. (H, I) The proportion of MyHC^+^ cells of si-circMEF2A2, siRNA-NC, ov-circMEF2A2, and ov-NC transfected SMSCs was calculated by Image pro plus software, n = 9. (J, K) qRT-PCR analysis of myogenic genes in the cDNA samples generated from lentivirus packaged circMEF2A2 shRNA (LV-si-circMEF2A2), LV-si-NC, overexpression vector (LV-ov-circMEF2A2), and LV-ov-NC infected breast muscles of Tianfu chicks, n = 3. (L, M) The breast muscle rate of the LV-si-circMEF2A2, LV-si-NC, LV-ov-circMEF2A2, and LV-ov-NC infected Tianfu chicks, n = 6. (N, O) Representative photographs of the unilateral breast muscles of the LV-si-circMEF2A2, LV-si-NC, LV-ov-circMEF2A2, and LV-ov-NC infected Tianfu chicks. (P, Q) Hematoxylin and eosin (H&E) staining of the cross-section of LV-si-circMEF2A2, LV-si-NC, LV-ov-circMEF2A2, and LV-ov-NC infected chicks’ breast muscle. Scale bars: 200 μm. (R, S) The myofiber cross-sectional area of LV-si-circMEF2A2, LV-si-NC, LV-ov-circMEF2A2, and LV-ov-NC infected chicks’ breast muscles calculated by image J software, n = 9. Data were displayed as mean ± SEM, independent sample *t*-test was used to analyze the statistical differences between each dataset, **P < 0.01 and *P < 0.05.

To investigate the function of the other member of circMEF2As, circMEF2A2, an overexpression vector (ov-circMEF2A2) and a siRNA (si-circMEF2A2) were employed to modulate the expression of circMEF2A2 in SMSCs following a screening process (Figs 5A, 5B and S3C). Knockdown of circMEF2A2 led to downregulation of myogenic marker genes (Fig 5C), a smaller relative myotube area, and less MyHC^+^ cells (Fig 5E, 5F and 5H), whereas overexpression of circMEF2A2 resulted in increased expression of myogenic genes (Fig 5D) and a faster rate of myotube synthesis (Fig 5E, 5G and 5I). These results suggest that circMEF2A2 facilitates myogenic differentiation.

Lentivirus-mediated in vivo analysis was also employed to investigate the role of circMEF2A2 in skeletal muscle growth. Specific lentiviruses were used to regulate the expression of circMEF2A2 in vivo (S8A and S8B Fig). Interference with circMEF2A2 in vivo (LV-si-circMEF2A2) resulted in breast muscle mass waste, a reduction in breast muscle rate, diminution of muscle fibers’ cross-sectional area, and disrupted expression of myogenic differentiation marker genes, whereas overexpression of circMEF2A2 in vivo (LV-ov-circMEF2A2) produced the opposite result (Figs 5J–5S and S8C–S8F). These findings indicate that circMEF2A2 positively regulates skeletal muscle development.

### 2.5 CircMEF2A2 activates the β-catenin pathway to promote myogenesis by targeting the miR-148a-5p/SLIT3 axis

**Fig 6:**
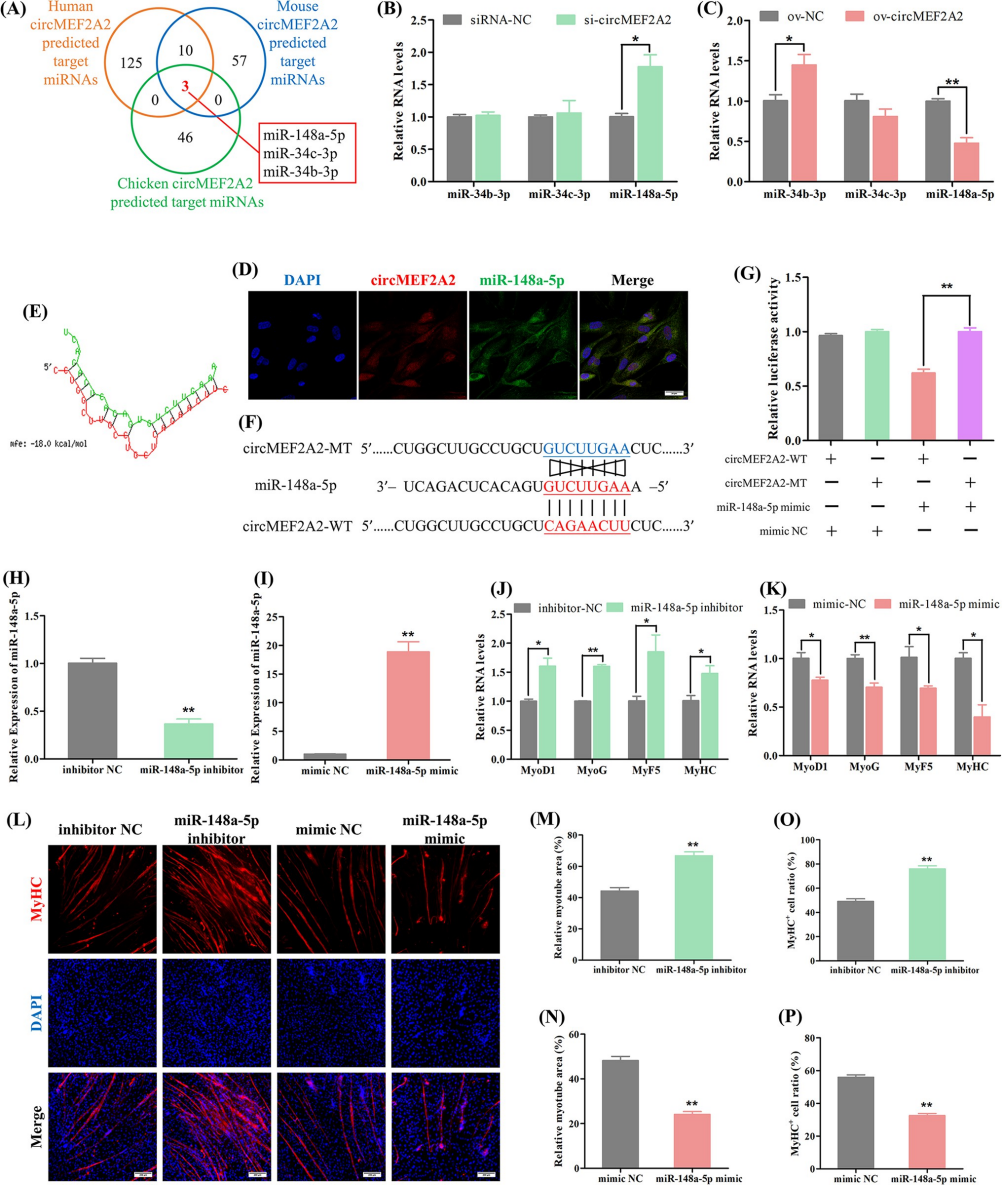
CircMEF2A2 targets and regulates miR-148a-5p. (A) Venn analysis of human, mouse, and chicken circMEF2A2 targeted miRNAs which predicted by RNAhybrid software, three co-targeted miRNAs including miR-148a-5p, miR-34b-3p, and miR-34c-3p were observed. (B, C) qRT-PCR analysis of these three co-targeted miRNAs in the cDNA samples generated from si-circMEF2A2, siRNA-NC, ov-circMEF2A2, and ov-NC transfected SMSCs, n = 3. (D) RNA FISH analysis revealed the subcellular localization of circMEF2A2 and miR-148a-5p in normal growing SMSCs. Scale bars: 20 μm. (E) The image of circMEF2A2 and miR-148a-5p hybridization which was predicted by RNAhybrid software. (F) Partial sequence of circMEF2A2 containing wild-type (circMEF2A2-WT) and mutant-type (circMEF2A2-MT) of miR-148a-5p response element were subcloned into the dual-luciferase reporter vector. (G) Dual-luciferase report analysis of circMEF2A2-WT and circMEF2A2-MT in DF-1 cells which co-transfected with miR-148a-5p mimic or mimic NC, n = 3. (H, I) qRT-PCR analysis of miR-148a-5p in cDNA samples generated from miR-148a-5p inhibitor, inhibitor NC, miR-148a-5p mimic and mimic NC transfected SMSCs, n = 3. (J, K) qRT-PCR analysis of myogenic genes in the cDNA samples generated from miR-148a-5p inhibitor, inhibitor NC, miR-148a-5p mimic and mimic NC transfected SMSCs, n = 3. (L) Immunofluorescence of MyHC in miR-148a-5p inhibitor, inhibitor NC, miR-148a-5p mimic and mimic NC transfected SMSCs. Scale bars: 200 μm. (M, N) The relative myotube area of miR-148a-5p inhibitor, inhibitor NC, miR-148a-5p mimic, and mimic NC transfected SMSCs was calculated by Image pro plus software, n = 9. (O, P) The proportion of MyHC^+^ cells of miR-148a-5p inhibitor, inhibitor NC, miR-148a-5p mimic and mimic NC transfected SMSCs was calculated by Image pro plus software, n = 9. Data were displayed as mean ± SEM, independent sample *t*-test was used to analyze the statistical differences between each dataset, **P < 0.01 and *P < 0.05.

CircMEF2A2 was predominantly localized in the cytoplasm and exhibited interaction with the AGO2 protein (S5 Fig), suggesting its potential role as a miRNA sponge, similar to circMEF2A1. Predicted target miRNAs of circMEF2A2 were subjected to Venn analysis, which revealed the co-targeting of three miRNAs, namely miR-34b-3p, miR-34c-3p, and miR-148a-5p, in human, mouse, and chicken (Figs 6A and S9A). Notably, miR-148a-5p was significantly negatively regulated by circMEF2A2 (Fig 6B and 6C). RNA-FISH analysis demonstrated the similar subcellular distribution of circMEF2A2 and miR-148a-5p (Fig 6D), and the dual-luciferase reporter assay confirmed the interaction between miR-148a-5p and the binding site on the circMEF2A2 sequence (Fig 6E–6G). Furthermore, we verified the function of miR-148a-5p in myogenesis by transfecting specific miRNA oligonucleotides into SMSCs (Fig 6H and 6I). The results revealed that miR-148a-5p inhibition induced the expression of myogenic differentiation marker genes and increased the relative myotube area and the number of MyHC^+^ cells, whereas excessive miR-148a-5p in SMSCs yielded a negative result, inhibiting the myogenic differentiation of SMSCs (Fig 6J–6P). Our findings suggest that circMEF2A2 can effectively target and inhibit miR-148a-5p.

**Fig 7:**
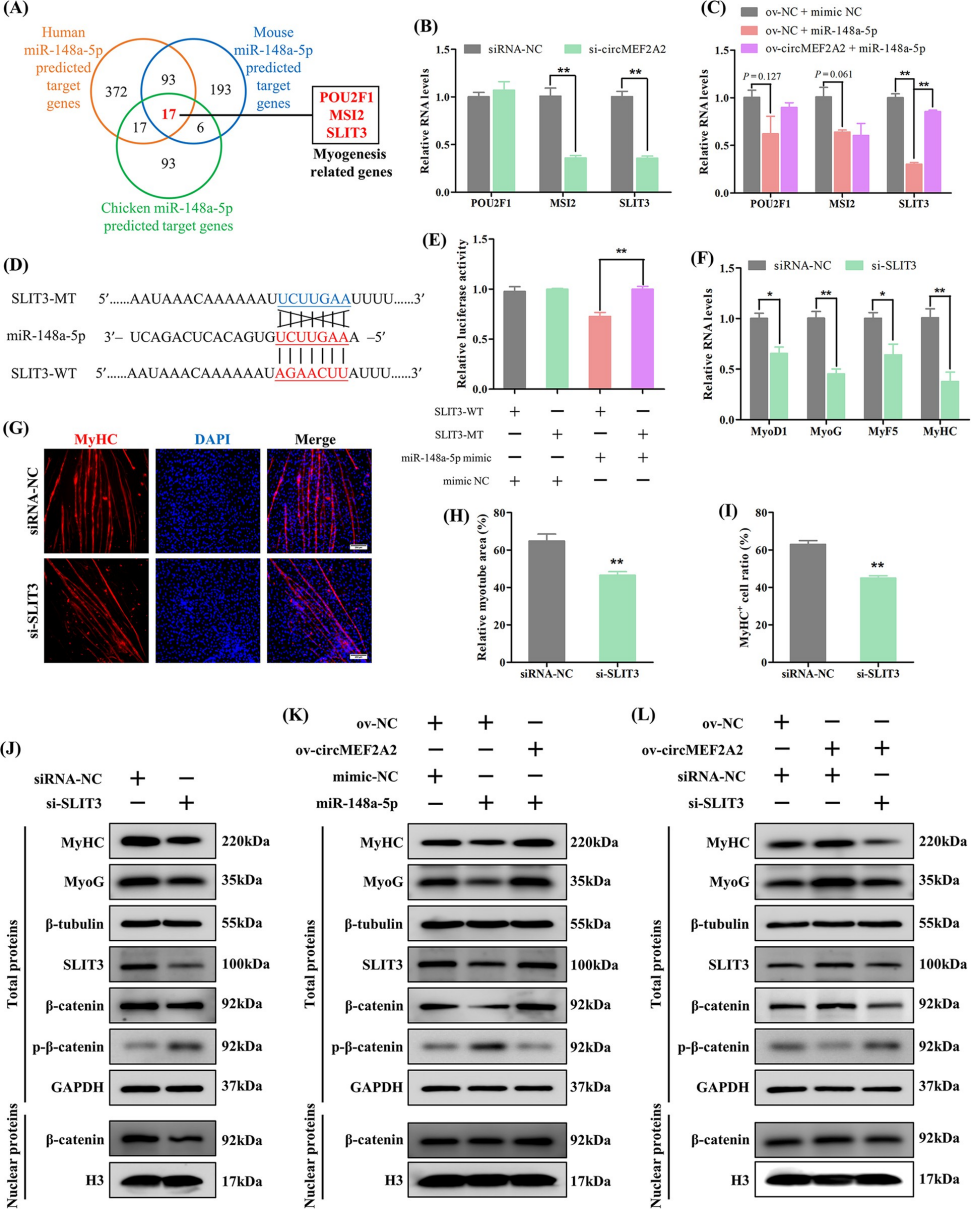
CircMEF2A2 activates the β-catenin pathway to promote myogenesis by targeting the miR-148a-5p/SLIT3 axis. (A) Venn analysis revealed human, mouse, and chicken miR-148a-5p was predicted co-targets 17 genes, and 3 myogenesis relative genes were identified among these 17 genes. (B) qRT-PCR analysis of these 3 myogenesis-relative genes in the cDNA samples generated from si-circMEF2A2 and siRNA-NC transfected SMSCs, n = 3. (C) qRT-PCR analysis of these 3 myogenesis relative genes in the cDNA samples generated from miR-148a-5p mimic or mimic NC and ov-circMEF2A2 or ov-NC co-transfected SMSCs, n = 3. (D) Partial sequence of SLIT3 3’UTR containing wild-type (SLIT3-WT) and mutant-type (SLIT3-MT) of miR-148a-5p response element were subcloned into the dual-luciferase reporter vector. (E) Dual-luciferase report analysis of SLIT3-WT and SLIT3-MT in DF-1 cells which co-transfected with miR-148a-5p mimic or mimic NC, n = 3. (F) qRT-PCR analysis of myogenic genes in the cDNA samples generated from SLIT3 siRNA (si-SLIT3) and siRNA-NC transfected SMSCs, n = 3. (G) Immunofluorescence of MyHC in si-SLIT3 and siRNA-NC transfected SMSCs. Scale bars: 200 μm. (H) The relative myotube area of si-SLIT3 and siRNA-NC transfected SMSCs was calculated by Image pro plus software, n = 9. (I) The proportion of MyHC^+^ cells of si-SLIT3 and siRNA-NC transfected SMSCs was calculated by Image pro plus software, n = 9. (J) Western blot analysis of myogenic proteins, and circMEF2A2/miR-148a-5p target proteins including SLIT3, β-catenin and p-β-catenin, and housekeeper proteins, in total proteins or nuclear proteins extracted from si-SLIT3 and siRNA-NC transfected SMSCs. (K) Western blot analysis of myogenic proteins, circMEF2A2/miR-148a-5p target proteins, and housekeeper proteins in total proteins or nuclear proteins extracted from miR-148a-5p mimic or mimic NC and ov-circMEF2A2 or ov-NC co-transfected SMSCs. (L) Western blot analysis of myogenic proteins, circMEF2A2/miR-148a-5p target proteins, and housekeeper proteins in total proteins or nuclear proteins extracted from si-SLIT3 or siRNA-NC and ov-circMEF2A2 or ov-NC co-transfected SMSCs. Data were displayed as mean ± SEM, independent sample *t*-test was used to analyze the statistical differences between each dataset, **P < 0.01 and *P < 0.05.

Subsequently, we performed bioinformatic analyses to identify target genes regulated by circMEF2A2 and miR-148a-5p. The results showed that 17 potential target genes of miR-148a-5p are evolutionarily conserved in humans, mice, and chickens (Figs 7A, S9B and S9C). Among these genes, POU class 2 homeobox 1 (POU2F1) [29], Slit guidance ligand 3 (SLIT3) [30], and Musashi RNA binding protein 2 (MSI2) [31] were reported to be related to muscle development and repair, but only SLIT3 was found to be jointly regulated by circMEF2A2 and miR-148a-5p (Fig 7B and 7C). Furthermore, the dual-luciferase reporter assay confirmed the direct targeting of the site on SLIT3 3’UTR by miR-148a-5p (Fig 7D and 7E). We then examined the role of SLIT3 in SMSC differentiation by transfecting specific siRNA (S3D Fig), and the results showed that the knockdown of SLIT3 reduced the expression of muscle differentiation marker genes (Fig 7F), and decreased the relative myotube area and the percentage of MyHC^+^ cells (Fig 7G–7I). Our data indicate that circMEF2A2 controls myogenesis by targeting miR-148a-5p to regulate the expression of SLIT3.

Previous studies have demonstrated that SLIT3 promotes myoblast differentiation and muscle regeneration by activating the Roundabout guidance receptor 2 (ROBO2)/ Catenin beta 1 (β-catenin) signaling pathway [30]. We also examined the marker protein of the β-catenin signal by western blotting and found that knockdown of SLIT3 not only reduced the expression of muscle differentiation genes but also promoted the phosphorylation of β-catenin protein, inhibiting its entry into the nucleus for transcriptional activity (Fig 7J). Overexpression of miR-148a-5p reduced the expression of SLIT3, resulting in the negative regulation of the β-catenin signal and myogenic differentiation. However, this inhibition effect of miR-148a-5p was reversed by the increase of circMEF2A2 (Fig 7K). Furthermore, circMEF2A2 overexpression stimulated the activation of the β-catenin signal and promoted the expression of myogenic differentiation marker genes. Nevertheless, the effect of circMEF2A2 was hindered by the reduction of SLIT3 (Fig 7L) and blocked by the reduction of the SLIT3 receptor ROBO2 (S3E and S10 Figs). These findings suggest the critical role of the circMEF2A2/miR-148a-5p/SLIT3/ROBO2/β-catenin axis in promoting myogenesis.

### 2.6 The relation between circMEF2As and MEF2A

Previous study have demonstrated that the MEF2A protein plays a positive role in the development of skeletal muscle [9]. In this study, we aimed to verify this finding in the chicken model. Specifically, we utilized siRNAs to down-regulate the expression of MEF2A in SMSCs (S3F Fig). Our results showed that the knockdown of MEF2A significantly reduced the expression of myogenic differentiation marker genes and inhibited the myotube formation of SMSCs (S11A–S11D Fig), indicating the conservation of the positive function of MEF2A across different species.

**Fig 8:**
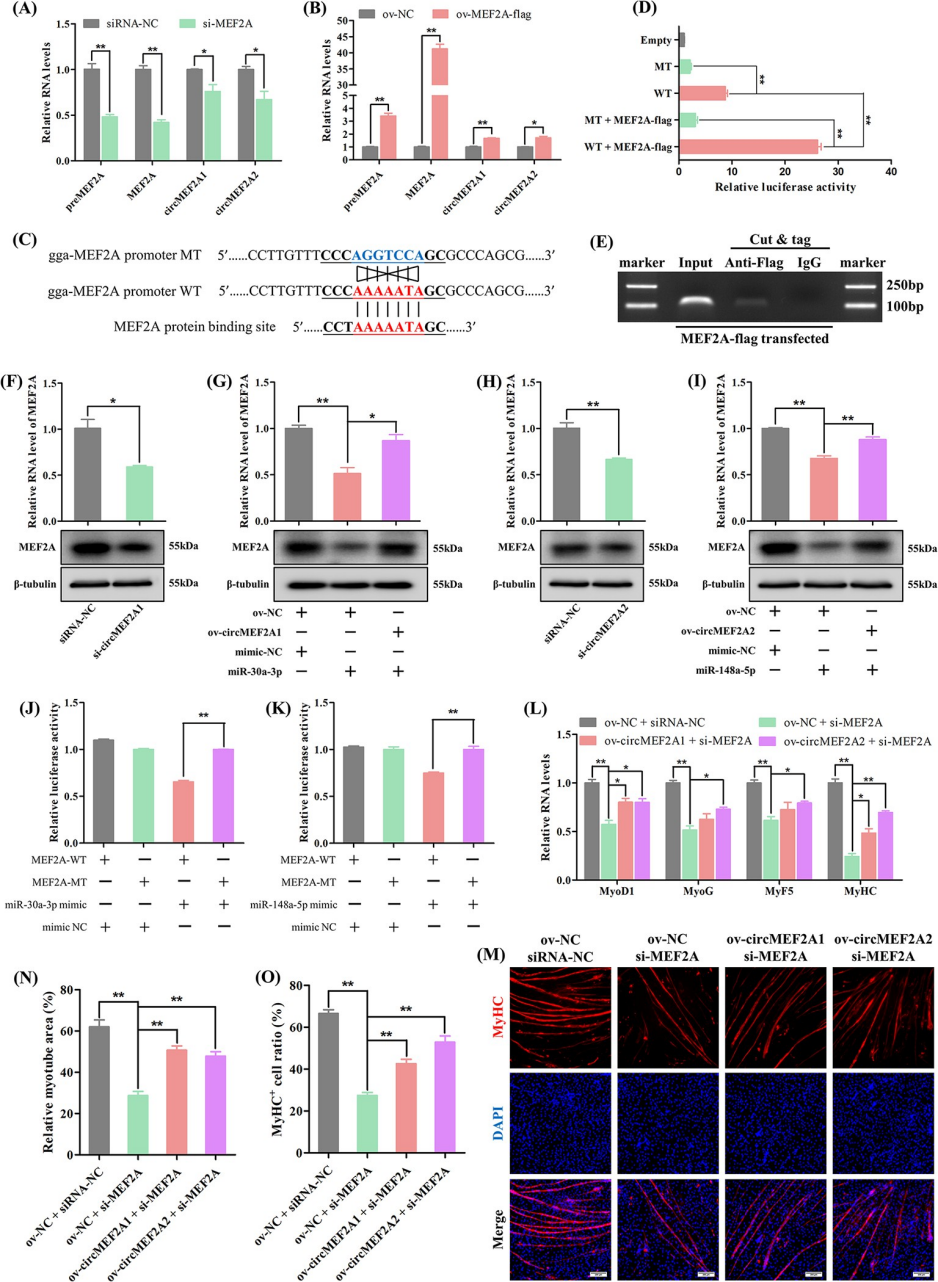
The relation between circMEF2As and MEF2A. (A) qRT-PCR analysis of linear MEF2A, preMEF2A, circMEF2A1, and circMEF2A2 in the cDNA samples generated from MEF2A siRNA (si-MEF2A) and siRNA-NC transfected SMSCs, n = 3. (B) qRT-PCR analysis of linear MEF2A, preMEF2A, circMEF2A1, and circMEF2A2 in the cDNA samples generated from MEF2A-flag fusion protein overexpression vector (ov-MEF2A-flag) and ov-NC transfected SMSCs, n = 3. (C) Partial MEF2A gene promoter sequences containing MEF2A protein binding site, the chicken MEF2A promoter sequence segment containing wild-type (WT) and mutant-type (MT) of MEF2A protein binding site were subcloned into promoter activity analyze dual-luciferase reporter vector. (D) Dual-luciferase report analysis of empty vector, promoter WT and promoter MT transfected SMSCs, additionally, promoter WT and promoter MT also co-transfected with ov-MEF2A-flag independently, n = 3. (E) Cut & tag PCR analysis were performed to test MEF2A protein binding ability on MEF2A promoter in ov-MEF2A-flag transfected SMSCs; DNA marker: DL2000. (F) RNA level and protein level of MEF2A in si-circMEF2A1 and siRNA-NC transfected SMSCs, n = 3. (G) RNA level and protein level of MEF2A in ov-circMEF2A1 or ov-NC and miR-30a-3p mimic or mimic NC co-transfected SMSCs, n = 3. (H) RNA level and protein level of MEF2A in si-circMEF2A2 and siRNA-NC transfected SMSCs, n = 3. (I) RNA level and protein level of MEF2A in ov-circMEF2A2 or ov-NC and miR-148a-5p mimic or mimic NC co-transfected SMSCs, n = 3. (J) Dual-luciferase report analysis of MEF2A-WT and MEF2A-MT in DF-1 cells which co-transfected with miR-30a-3p mimic or mimic NC, n = 3. (K) Dual-luciferase report analysis of MEF2A-WT and MEF2A-MT in DF-1 cells which co-transfected with miR-148a-5p mimic or mimic NC, n = 3. (L) qRT-PCR analysis of myogenic genes in the cDNA samples generated from ov-circMEF2A1, ov-circMEF2A2, ov-NC, siRNA-NC, and si-MEF2A co-transfected SMSCs, n = 3. (M) Immunofluorescence of MyHC in ov-circMEF2A1, ov-circMEF2A2, ov-NC, siRNA-NC, and si-MEF2A co-transfected SMSCs. Scale bars: 200 μm. (N) The relative myotube area of ov-circMEF2A1, ov-circMEF2A2, ov-NC, siRNA-NC, and si-MEF2A co-transfected SMSCs was calculated by Image pro plus software, n = 9. (O) The proportion of MyHC^+^ cells of ov-circMEF2A1, ov-circMEF2A2, ov-NC, siRNA-NC, and si-MEF2A co-transfected SMSCs was calculated by Image pro plus software, n = 9. Data were displayed as mean ± SEM, independent sample *t*-test was used to analyze the statistical differences between each dataset, **P < 0.01 and *P < 0.05.

Additionally, we discovered that circMEF2As can also promote the development of chicken skeletal muscle. Thus, we sought to explore the relationship between linear MEF2A and circMEF2As. Knockdown of MEF2A led to a significant decrease in the abundance of both linear MEF2A and circMEF2As (Fig 8A). Given that linear MEF2A-derived protein is a transcription factor, we speculated that linear MEF2A may regulate the expression of circMEF2As at the transcriptional level. We tested this hypothesis by detecting the expression of the MEF2A RNA precursor (preMEF2A) upon MEF2A interference, and the results showed a reduction in preMEF2A expression (Fig 8A). Besides, the expression of preMEF2A, linear MEF2A, and circMEF2As were upregulated by the overexpression of the Flag-tagged MEF2A protein (Fig 8B). We further confirmed the speculation by analyzing the promoter sequence of the chicken MEF2A gene and identifying a MEF2A protein binding element approximately 10bp upstream of the MEF2A transcription start site (Figs 8C and S11E–S11H). Importantly, this binding element was found to be highly conserved among humans, mice, and chickens (S11I Fig). We also discovered strong binding signals of MEF2A protein on the MEF2A promoter in humans and mice by searching a chip-seq database (S11J and S11K Fig). To confirm these findings in the chicken model, we constructed a promoter activity validation reporter of the MEF2A promoter containing either wild-type or mutant-type MEF2A protein binding elements. Our results showed that the promoter activity of the wild-type reporter was significantly higher than that of the mutant-type reporter, and it was further stimulated by the flag-labeled MEF2A protein (Fig 8D). Finally, Cut & tag technology was utilized to test the interaction between transcription factors and DNA sequences (Fig 8E), revealing that the MEF2A protein can directly interact with its own promoter. Collectively, these results suggest that linear MEF2A-derived protein can regulate the expression of both linear MEF2A and circMEF2As at the transcriptional level by binding to its own promoter.

CircMEF2As have been shown to regulate gene expression through the ceRNA mechanism. To investigate whether circMEF2As regulate the RNA and protein expression of MEF2A by binding miRNAs, a series of experiments were conducted. qRT-PCR and western blot results demonstrated that the knockdown of circMEF2As led to down-regulation of both the RNA and protein level of MEF2A (Fig 8F and 8H). This effect was also observed upon overexpression of miR-30a-3p and miR-148a-5p, and overexpression of circMEF2As relieved the inhibition of these miRNAs on MEF2A expression (Fig 8G and 8I). Subsequent prediction of the binding element of miR-30a-3p and miR-148a-5p on the linear MEF2A mRNA revealed that miR-30a-3p targets the 5’UTR of the linear MEF2A mRNA, while miR-148a-5p targets the coding sequence of the linear MEF2A mRNA, with the binding element being conserved among species (S12 Fig). Dual-luciferase reporter analysis further confirmed the direct interaction of miR-30a-3p and miR-148a-5p with their respective binding elements (Fig 8J and 8K). Taken together, our results suggest that circMEF2As positively regulate the linear MEF2A through the ceRNA mechanism.

Given the importance of the MEF2A protein in myogenic differentiation, we investigated whether circMEF2As can alleviate the obstruction in myogenesis caused by MEF2A deficiency. Results from qRT-PCR and immunofluorescence analysis showed that the knockdown of MEF2A decreased the expression of myogenic marker genes and inhibited the formation of myotubes in SMSCs, interestingly, the inhibition effect of MEF2A knockdown in myogenesis was partially rescued by circMEF2As, although not completely (Fig 8L–8O). These findings suggest that both linear MEF2A and circMEF2As play important and independent roles in myogenic differentiation.

### 2.7 Conservative function of circMEF2As in mouse myogenesis

**Fig 9:**
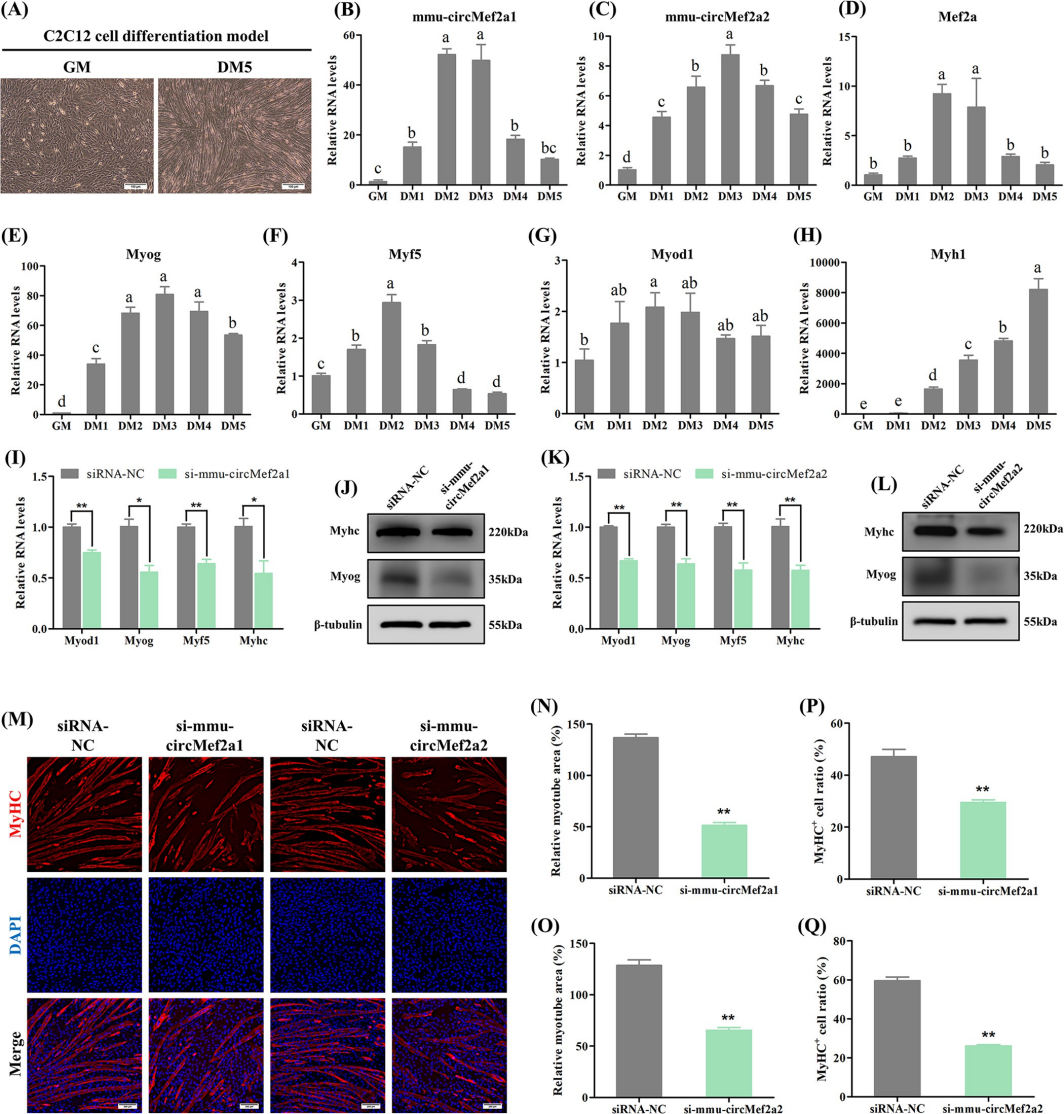
Conservative function of circMEF2As in mouse myogenesis. (A) Representative images of the C2C12 cells cultured in GM and cultured in DM for 5 days (DM5). (B-H) qRT-PCR analysis of mmu-circMef2a1, mmu-circMef2a2, and 5 mouse myogenic genes including Mef2a, Myog, Myf5, Myod1, and Myh1 in the cDNA samples generated from the C2C12 cells at the proliferative stage and induced to differentiate for 1 day (DM1), 2 days (DM2), 3 days (DM3), 4 days (DM4) and 5 days (DM5), n = 3. (I) qRT-PCR analysis of myogenic genes in the cDNA samples generated from the mmu-circMef2a1 siRNA (si-mmu-circMef2a1) and siRNA-NC transfected C2C12 cells, n = 3. (J) West blot analysis of Myhc, Myog, and β-tubulin proteins in si-mmu-circMef2a1 and siRNA-NC transfected C2C12 cells. (K) qRT-PCR analysis of myogenic genes in the cDNA samples generated from the mmu-circMef2a2 siRNA (si-mmu-circMef2a2) and siRNA-NC transfected C2C12 cells, n = 3. (L) West blot analysis of Myhc, Myog, and β-tubulin proteins in si-mmu-circMef2a2 and siRNA-NC transfected C2C12 cells. (M) Immunofluorescence of MyHC in si-mmu-circMef2a1, si-mmu-circMef2a2, and siRNA-NC transfected C2C12 cells. Scale bars: 200 μm. (N, O) The relative myotube area of si-mmu-circMef2a1, si-mmu-circMef2a2, and siRNA-NC transfected C2C12 cells was calculated by Image pro plus software, n = 9. (P, Q) The proportion of MyHC^+^ cells of si-mmu-circMef2a1, si-mmu-circMef2a2, and siRNA-NC transfected C2C12 cells was calculated by Image pro plus software, n = 9. Data were displayed as mean ± SEM, independent sample *t*-test (I, K and N-Q), and one-way ANOVA analysis (B-H) were used to analyze the statistical differences between each dataset, **P < 0.01, *P < 0.05, and ^a, b, c^ *P* < 0.05.

Genes that are evolutionarily conserved are typically more functionally significant [25]. Having observed the promotion of myogenesis by circMEF2As in chicken, we aimed to investigate whether the function of circMEF2As is conserved across species by examining the role of mouse circMef2as (mmu-circMef2a1 and mmu-circMef2a2) in mouse myogenesis using C2C12 cells. Initially, we established the C2C12 differentiation model, and observed the appearance of myotubes during myogenic differentiation (Fig 9A). Myogenic marker genes, including Myog, Myod1, Myf5, Myh1, and Mef2a, were induced by the differentiation process, and mmu-circMef2a1 and mmu-circMef2a2 were also highly expressed during differentiation, with expression patterns that were extremely similar to their host gene Mef2a (Fig 9B–9H), suggesting their potential role in myogenesis. Subsequently, we used siRNAs to investigate the role of mmu-circMef2as in C2C12 cell differentiation (S3G and S3H Fig). Results showed that the knockdown of mmu-circMef2as significantly reduced the RNA and protein levels of the myogenic marker genes (Fig 9I–9L), as well as inhibited myotube formation in C2C12 cells (Fig 9M–9Q). Together, our findings reveal the conserved function of circMEF2As in myogenesis across species.

## 3 Discussion

The growth and repair of skeletal muscle represent critical research areas in human medicine and agricultural animal production. These processes predominantly rely on microcosmic myogenesis and are regulated by several key genes, including the MRF and MEF families. The MEF2 family was the first to be identified as interacting with the enhancer of the muscle creatine kinase gene [32], and subsequently, MEF2A, an essential member of the MEF2 family, was discovered to be involved in skeletal muscle development [33], heart function [34], and brain activities [35]. MEF2A regulates gene expression through transcriptional regulation, whereby it binds to the promoter of target genes via its MADS DNA binding domain [36,37]. In myogenic differentiation, MEF2A exerts unique transcriptional regulation effects on target genes [10]. Additionally, the Gtl2-Dio3 microRNA mega-cluster is also regulated by MEF2A and is involved in myogenesis regulation [38]. Despite extensive research into the alternative mRNAs and their encoded proteins produced by the MEF2A gene, the existence of MEF2A-generated functional non-coding RNAs remains unknown. In this study, we have identified two circRNAs, circMEF2A1 and circMEF2A2, produced by the MEF2A gene in chicken skeletal muscles. These circRNAs are generated from different exons of the MEF2A gene and share the same precursor RNA. Furthermore, we have confirmed their existence and circular form in skeletal muscle cells through various assays, highlighting their potential role in regulating skeletal muscle growth. On the other hand, we noticed that circMEF2As was only significantly expressed in muscle tissues, whereas MEF2A linear RNA was highly expressed in both muscle and brain tissues. We conjecture that circMEF2As production may be aided by an RNA-binding protein that is exclusively expressed in muscle tissue, which would prevent the production of more circMEF2As in brain tissue, yet additional experimental data is needed to prove it.

CircRNAs were initially considered transcriptional byproducts and were not taken seriously until their abundance and dynamic expression were revealed in animal cells with the advancement of life science and technology [13]. In the research field of skeletal muscle development, numerous circRNAs have been identified as participating in the regulation of the physiological process of myogenesis [14]. Most of these circRNAs are derived from non-organ-specific regulatory genes and may play similar roles in other tissues. However, circular MEF2D RNA is exceptional in exhibiting a distinct function from MEF2D protein by inhibiting bovine myoblast proliferation and differentiation [39]. In this study, we identified circMEF2As as being generated from MEF2A and specifically enriched in heart and skeletal muscle tissues, suggesting that circMEF2As may play roles in muscle tissue development. Through a series of molecular biological technologies, we have ascertained that circMEF2A1 facilitates myogenic differentiation in vitro and promotes skeletal muscle growth in vivo by regulating the miR-30a-3p/PPP3CA/NFATC1 signal during myogenesis. Similarly, circMEF2A2 promotes skeletal muscle development by activating the SLIT3/ROBO2/β-catenin signaling via targeting miR-148a-5p. Unlike MEF2D and circMEF2D, the function of circMEF2As in myogenesis is the same as the host gene MEF2A, yet they are capable of independently performing functions without relying on MEF2A protein. These findings highlight the complex functional modality of the MEF2A gene. Additionally, circRNAs functions in several other ways, including adsorbing RNA-binding proteins or directly translating into proteins [40], more study is required to discover that whether circMEF2As influences skeletal muscle growth in these ways.

Currently, a great deal of research has emerged on the applications of circRNAs in human medicine, many circRNAs that contribute to the development of disease have been identified and targeting these circRNAs can be therapeutic for the disease substantially [41,42]. On the other hand, engineered circRNAs is capable of adsorbing miRNAs or translating large amounts of proteins in vivo due to its slow degradation rate, which can be utilized as a new strategy for in vivo therapy [43,44]. These studies demonstrate the great potential of circRNAs in human medicine, but the utilization depends on the function and molecular regulatory mechanisms of the target circRNAs in human disease. In this study, we have endeavored to investigate the conserved regulatory pathways of circMEF2As in humans, mice, and chickens, with the aim of providing novel insights into human medicine. However, a more precise understanding of the molecular mechanisms underlying the regulation of skeletal muscle development by circMEF2As in humans and mice remains an area of active research.

Prior studies have suggested that there is no clear correlation between the abundance of mRNA and circRNA produced from the same host gene at the macro transcriptional level [45]. Moreover, the functions of circRNAs may be independent of their host genes [46], although some circRNAs have been found to interact with their respective host genes. For example, circSMARCA5 can form an R-loop with its host gene and lead to transcriptional pausing of its host gene [47], while circSIRT1 can enhance the expression of its host gene Sirtuin 1 (SIRT1) through promoting its deubiquitylation and stabilization [48]. Additionally, Muscleblind (MBL) protein can accelerate the biogenesis of circMBL by binding to its flanking introns [49]. In our study, we investigated the interaction between circMEF2As and MEF2A. MEF2A has been reported to autoregulate in a transcriptional manner [50]. Our findings suggest that MEF2A can form an autoregulated positive feedback loop by binding to its own promoter, which increases the output of both linear MEF2A mRNA and circMEF2As. Furthermore, we discovered that circMEF2As can positively regulate the expression of linear MEF2A through the ceRNA mechanism, indicating their involvement in the feedback loop of MEF2A autoregulation. In addition, we also observed that MEF2A deficiency resulted in impaired myogenesis, as knockdown of MEF2A inhibited the myogenic differentiation of SMSCs. Overexpression of circMEF2As while knocking down MEF2A did not fully rescue the negative effect of MEF2A deficiency on myogenesis. These results suggest that the transcriptional regulation of MEF2A is critical for muscle cell differentiation, and while circMEF2As and MEF2A may regulate each other, their functions are relatively independent and cannot be substituted for each other.

In the context of evolution, the procreation of life involves random gene mutations, and advantageous genotypes are retained under environmental stress, leading to genetic differentiation and further speciation [51,52]. Hence, crucial genes such as MEF2A are conserved throughout evolution and widely expressed in eukaryotes, indicating their essential roles in vital life processes in animals, plants, and fungi. Numerous studies have demonstrated that certain circRNAs are highly conserved across different species [53]. For instance, CircRNA CDR1as is crucial for the physiological functions of both human and mouse brains [54], whereas CircZNF609 is conservatively expressed in human and mouse muscles and contributes to muscle repair [55]. Nevertheless, there are also contradictory perspectives that circRNAs are simply the byproducts of splicing errors arising from the genome [56]. In our investigation, we examined the circAtlas database and discovered that MEF2A gene can produce two conserved circMEF2As in humans, macaques, mice, rats, chickens, and pigs. Furthermore, we detected the presence of these circMEF2As in the muscle cells of chickens and mice, and their function in myogenesis is conserved in these species. Due to the strikingly similar sequence of circMEF2As across different species, convergent evolution seems implausible. Therefore, we propose that circMEF2As are the outcome of orthologous evolution. Based on the speciation time recorded in the TimeTree database, we speculate that circMEF2As originated from the common ancestor of chickens and mice at least 350 million years ago and subsequently spread to other mammals and birds (S2G Fig). Whether circMEF2As are conserved across a broader range of species requires further investigation. Overall, our study provides compelling evidence that circRNAs are conserved and play critical roles in various biological processes.

**Fig 10:**
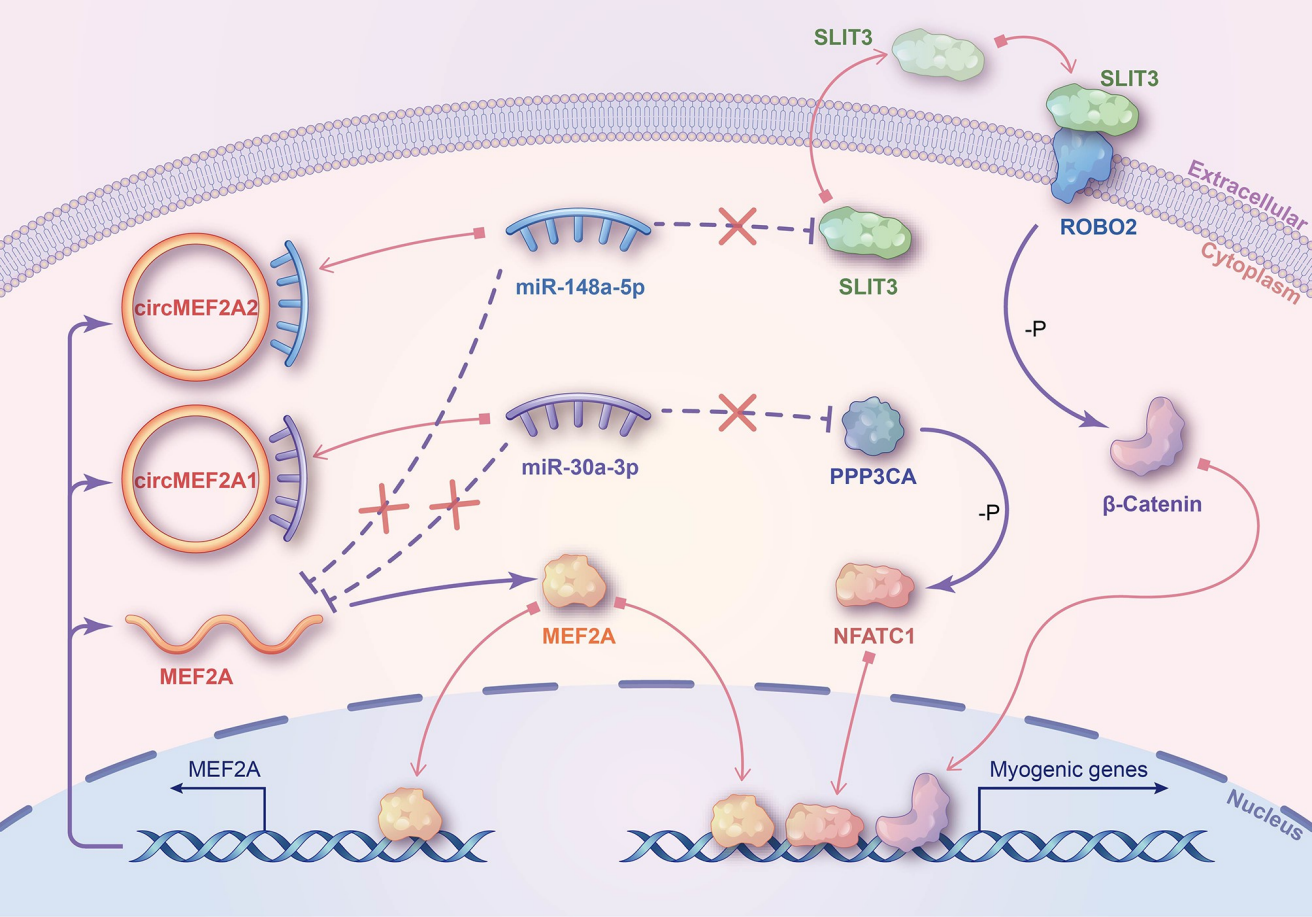
Schematic diagram of the molecular mechanism of circMEF2As regulating myogenesis. MEF2A protein promotes linear MEF2A and circMEF2As biogenesis through a transcription manner. CircMEF2A1 activates PPP3CA/NFATC1 signaling by targets miR-30a-3p, and circMEF2A2 activates SLIT3/ROBO2/β-Catenin signaling by targets miR-148a-5p. circMEF2As also regulate the expression of linear MEF2A via target miR-30a-3p and miR-148a-5p. MEF2A, NFATC1, and β-catenin proteins transfer to the nucleus to activate the transcription of myogenic genes.

In summary, our study has demonstrated that the MEF2A gene produces two evolutionarily conserved circRNAs, namely circMEF2A1 and circMEF2A2. CircMEF2A1 promotes myogenesis by regulating the miR-30a-3p/PPP3CA/NFATC1 axis, while circMEF2A2 facilitates myogenic differentiation by targeting the miR-148a-5p/SLIT3/ROBO2/β-catenin signaling pathway. Furthermore, we observed a reciprocal upregulation of circMEF2As and linear MEF2A expression, and an independent regulation of skeletal muscle development by these molecules in different ways (Fig 10). Importantly, our results also demonstrate the conservation of circMEF2As’ functions in myogenesis across chickens and mice. These findings have uncovered the intricate roles of the MEF2A gene in myogenesis and have highlighted the conservation and significance of circMEF2As during evolution. Our research may provide novel insights into the fields of human medicine, farm animal production, animal genetics, and evolution.

## 4 Materials and methods

### 4.1 Ethics statement

There are no any human tissues or cells used in this research. The feeding, care, and execution of experimental animals in this study adhered to the regulations prescribed by the Animal Welfare Committee of Sichuan Agricultural University (approve number: 2020102012). Specifically, 1-day-old Tianfu broilers were selected as the animal model and were reared under controlled conditions at the poultry breeding farm of Sichuan Agricultural University.

### 4.2 Cell culture

SMSCs were isolated from the breast muscles of 4-day-old Tianfu broilers following humane procedures. Briefly, chicks were anesthetized and euthanized, and the breast muscle tissues were meticulously dissected from the bone. The muscle tissue was then minced and homogenized with ophthalmic scissors. The muscle homogenate was digested with Collagenase I and Trypsin (Gibco, Langley, USA), filtered through 70 μm and 40 μm strainer, and isolated by centrifugation. Next, the cells were resuspended in a growth medium (GM) and cultured under controlled conditions (37°C, 5% CO_2_, humidified atmosphere) in cell culture plates. The cells were plated twice for 3 hours to eliminate fibroblasts and further SMSCs were counted and seeded in the culture plates. The GM was replaced with a differentiation medium (DM) to induce myogenic differentiation of SMSCs. DF-1 cells obtained from Fuheng biology (Shanghai, China) used for dual-luciferase reporter assays were cultured in the growth medium, while C2C12 cells (Fuheng biology) were utilized for investigating the function of mouse circMEF2As and were cultured in high glucose GM and DM. The formulation of GM and DM has been described in our previous study [24], all media were refreshed every 24 hours. Finally, the transfection of cells was conducted using the Lipofectamine 3000 Transfection Reagent (Invitrogen, Carlsbad, USA), in compliance with the manufacturer’s instructions.

### 4.3 Quantitative real-time PCR (qRT-PCR)

Total RNAs were extracted from tissues or cells using TRIzol reagent (TaKaRa, Otsu, Japan) following standard procedures. The quality and concentration of total RNAs were assessed using an ND-2000 nucleic acid and protein detector (NanoDrop Technologies, Wilmington, USA). Reverse transcription of mRNAs was performed using the PrimeScript RT Reagent with the gDNA Eraser Kit (TaKaRa) for cDNA synthesis, while the One Step miRNA cDNA Synthesis Kit (HaiGene, Haerbin, China) was used for miRNA reverse transcription. The expression levels of the target genes were quantified by qRT-PCR analysis using the TB Green PCR Master Mix (TaKaRa) and a CFX96-Touch real-time PCR detection system (Biorad, Hercules, USA). Internal reference genes, including β-actin, GAPDH, and U6, were utilized for normalizing RNA levels, and the relative expression levels of RNAs were calculated using the 2^-ΔΔCt^ method. The primers used in this study were designed using Premier 5 software, and detailed information is provided in S1 Table.

### 4.4 circRNA validation

The upstream and downstream sequences of the back-splicing junctions of circRNAs were selected to design divergent primers. The amplification of divergent primers using cDNA samples which generated from differentiating SMSCs or C2C12 cells, the amplification products were observed by agarose gel electrophoresis and identified by referring to the DL2000 DNA marker (TaKaRa), then sequenced by Sanger sequencing and mapped to the back-splicing junction of circRNAs.

For RNase R treatment, total RNAs extracted from differentiating SMSCs or C2C12 cells were divided into two parts. One part was incubated with 1 U/μl RNase R (Epicentre Technologies, Madison, USA) at 37°C for 10 minutes, while the other part underwent the same treatment with sterile water as a negative control. Reverse transcription was then performed to generate cDNA samples, and qRT-PCR analysis was used to analyze gene expression levels.

Furthermore, total RNAs extracted from differentiating SMSCs or C2C12 cells were divided into two parts. One part was reverse transcribed into cDNA using random N9 primers, while the other part was reverse transcribed using oligo d(T) primer. qRT-PCR analysis was then performed to analyze gene expression levels.

### 4.5 RNA oligonucleotides and overexpression vectors

The small interfering RNAs (siRNAs) targeting circMEF2A1, circMEF2A2, PPP3CA, SLIT3, MEF2A, ROBO2, mmu-circMEF2A1, mmu-circMEF2A2, as well as the oligonucleotides including miR-30a-3p mimic, miR-30a-3p inhibitor, miR-148a-5p mimic, and miR-148a-5p inhibitor were designed and synthesized by GenePharma Co., Ltd., (Shanghai, China) and their sequences can be found in the S2 Table. Moreover, the linear sequences of circMEF2As were chemically synthesized and subcloned into the pCD25-ciR vector (Geneseed Biotech, Guangzhou, China), and the resulting constructs were validated by Sanger sequencing. In the transfection assay, the concentration of siRNAs in medium was 100nM, and the concentration of plasmids in medium was 1ng/μL.

### 4.6 Dual-luciferase reporter assay

To verify circRNA-miRNA or miRNA-mRNA interactions, we synthesized sequences of circRNA or mRNA containing wild-type and mutant-type miRNA response elements and subcloned them into pmirGLO luciferase reporter (Tsingke Biotechnology, Beijing, China). DF-1 cells were seeded in the 48-well plates and co-transfected with the reporters and miRNA mimics, respectively. After 48 hours of transfection, the luciferase activity of Firefly and Renilla luciferases was measured using the Dual-Luciferase Reporter Gene Assay Kit (Beytime, Shanghai, China).

To analyze promoter activity, we synthesized the sequence of MEF2A promoter containing wild-type and mutant-type MEF2A protein binding sites and subcloned them into pEZX-FR01 luciferase reporter (GenePharma). DF-1 cells were seeded in the 48-well plates and then transfected with reporters alone or co-transfected with the MEF2A-flag overexpression vector, respectively. After transfection for 48 hours, the luciferase activity of Firefly and Renilla luciferases was measured using the Dual-Luciferase Reporter Gene Assay Kit (Beytime).

### 4.7 Fluorescence in situ hybridization (FISH)

The specific RNA FISH probes were custom synthesized (Tsingke Biotechnology) for the FISH assay, SMSCs were seeded on a cell-crawling piece in the 6-well plates and induced for differentiation for 24 hours, next harvested by fixed with 4% Paraformaldehyde (Beytime). The FISH assay was conducted using a Fluorescence in Situ Hybridization Kit (GenePharma) as per the manufacturer’s instructions. Confocal microscopy was used to capture the images. The sequences of the specific RNA FISH probes are available in S3 Table.

### 4.8 Western blotting

SMSCs were seeded into 6-well cell culture plates and transfected for 72 hours. Then the protein extraction was performed using a commercially available RIPA lysis buffer (Beytime), and protein concentration was quantified using a BCA assay Kit (Beytime). Subsequently, protein samples were resolved by 10% or 15% SDS-polyacrylamide gel electrophoresis, and the proteins were transferred onto PVDF membranes (Millipore, Bedford, USA) using a wet transfer method. The PVDF membranes were blocked with a blocking buffer (Beytime) at room temperature for 1 hour, and then incubated with primary antibodies overnight at 4°C. On the following day, after washing with TBST, the membranes were incubated with secondary antibodies (ZenBio, Chengdu, China; 1:1,000) for 1 hour at room temperature. Finally, protein bands were visualized using an Enhanced chemiluminescence reagent (Beytime), and a Gel imaging system (Biorad) was employed for image acquisition. The complete list of antibodies used is available in S4 Table.

### 4.9 Immunofluorescence

SMSCs were seeded into 48-well cell culture plates and transfected for 72 hours. After that, the cells were washed with phosphate-buffered saline (PBS) and fixed with 4% Paraformaldehyde for 20 minutes. The cells were then permeabilized using 0.5% Triton X-100 (Beytime) at room temperature for 30 minutes, and subsequently blocked with Goat serum (Beytime) for another 30 minutes. The SMSCs were then incubated overnight at 4°C with a diluted primary antibody specific to MyHC (Santa Cruz, CA, USA; 1:500). On the following day, the cells were washed with PBST and then incubated with a diluted fluorescent secondary antibody (ZenBio; 1:1,000) at 37°C for 1 hour. The cell nuclei were stained with DAPI for 5 minutes. Finally, the SMSCs were observed and photographed using a fluorescence microscope. The relative myotube area was calculated as the percentage of MyHC fluorescence area to the DAPI fluorescence area, and the MyHC positive cell ratio was determined as the percentage of the number of MyHC positive cell nuclei to the total number of cell nuclei. These calculations are performed by the Image pro plus software.

### 4.10 Lentivirus production and transduction

The short hairpin RNAs targeting circMEF2As were cloned into the pLV3ltr-Puro-U6 vector (named LV-si-circMEF2A1 and LV-si-circMEF2A2), while the linear sequence of circMEF2As and their flanking circularize element were cloned into the pLV4ltr-Puro-CMV-NC vector (named LV-ov-circMEF2A1 and LV-ov-circMEF2A2). The resulting vectors were co-transfected with the psPAX2 and pMD2.G vectors into HEK293T cells to generate lentivirus. After purification, the lentivirus titer was adjusted to 5 × 10^8^ Transduction Units (TU)/mL. Subsequently, a total volume of 100 μL, containing 1 × 10^7^ TU lentivirus, 0.1 μL of 4 mg/mL Polybrene (Tsingke biotechnology), and moderate PBS, were directly injected into the breast muscle of 1-day-old Tianfu broiler chicks (n = 6 for each group). This injection was repeated at a triple dose after 7 days. The chicks were allowed to incubate for 14 days, and then the breast muscle samples were harvested for H&E staining and qRT-PCR analysis. The cross-sectional area (CSA) of myofibers was determined by the Image J software.

### 4.11 RNA sequencing

SMSCs were cultured in a 6-well plate and transfected with circMEF2A1 siRNA and negative control siRNA for 36 hours. Total RNAs was extracted using TRIzol Reagent (TaKaRa) and DNase I (TaKaRa) was used to remove genomic DNA. RNA quality was assessed using the 2100 Bioanalyzer (Agilent Technologies, California, USA) and quantified with the ND-2000 (NanoDrop Technologies). A TruSeq RNA sample preparation kit (Illumina, San Diego, USA) was used to prepare RNA-seq transcriptome libraries, which were then sequenced using the Illumina HiSeq X Ten/NovaSeq 6000 sequencer (Illumina). The raw reads were trimmed and quality-controlled using SeqPrep and Sickle software, and the clean reads were aligned to the reference genome (Gallus gallus 5.0) using HISAT2 software. The transcripts’ expression levels were calculated using the transcripts per million reads (TPM) method, and differential gene expression analysis was performed using DESeq2 software, with a condition of fold change >1.2 and *P* value <0.05.

### 4.12 Cut & tag

SMSCs were cultured in a T25 flask and transfected with the MEF2A-Flag overexpression vector for 48 hours. Following this, SMSCs were collected, and the cut & tag assay was performed using the Hieff NGS G-Type In-Situ DNA Binding Profiling Library Prep Kit (Yeasen Biotechnology, Shanghai, China) in accordance with the manufacturer’s instructions. The Flag antibody (ZenBio; 1:20) was utilized to capture the flag-tagged MEF2A bound DNA sequence, with mouse normal IgG (ZenBio; 1:20) serving as the negative control. The binding site was subsequently amplified using a pair of cut & tag primers, and the amplification products were visualized through agarose gel electrophoresis.

### 4.13 RNA binding protein immunoprecipitation-qPCR (RIP-qPCR)

SMSCs were seeded in a T25 cell culture flask and harvested after induction of differentiation for 48 hours. The RIP assay was carried out using a Magna RNA Binding Protein Immunoprecipitation Kit (Millipore) following the manufacturer’s instructions. An AGO2 antibody was employed to perform the RIP assay, and AGO2-bound RNAs were isolated and reverse-transcribed into cDNA samples. Linear MEF2A and circMEF2As were subsequently detected by qRT-PCR analysis.

### 4.14 Nuclear-cytoplasmic fractionation

SMSCs were cultured in a T25 cell culture flask and harvested after induction of differentiation for 48 hours. The cell nucleus and cytoplasm were then separated using NE-PER nuclear and cytoplasmic extraction reagents (Thermo Fisher, Carlsbad, USA) following the manufacturer’s instructions. Total RNAs and proteins were extracted from the nucleus and cytoplasm samples separately, and were subjected to qRT-PCR analysis and western blot analysis, respectively, using standard methods.

### 4.15 Bioinformatics analysis

We utilized the CircAtlas database to identify circRNAs expressed across multiple species [57]. The Ensembl and National Center for Biotechnology Information (NCBI) databases were queried to obtain gene promoter or mRNA sequences. RNAhybrid web tool was employed to predict potential miRNA targets of the circRNAs [58]. The TargetScan [59] and miRDB [60] databases were utilized to identify the target genes of the miRNAs. The Draw Venn diagram web tool (http://bioinformatics.psb.ugent.be/webtools/Venn/) was utilized to identify the intersections between gene sets. Furthermore, the JASPAR database was employed to predict transcription factor binding sites [61], and the Cistrome database was used to identify binding signals of transcription factors in the genome [62]. In addition, MEGA7 software was used for evolutionary analysis [63], and TimeTree database was used to query species divergence time [64].

### 4.16 Statistical analysis

All experiments were conducted with at least three independent replicates. The data are presented as mean ± standard error of the mean (SEM) and were graphed using GraphPad Prism software. Statistical analysis was performed using SPSS 20 software, with independent sample *t*-tests for comparisons between two datasets and one-way ANOVA for comparisons between more than two datasets. Statistical significance is indicated as **P < 0.01, *P < 0.05, and lowercase letters (a, b, c) indicate *P* < 0.05.

## Supporting information

S1 Fig The retrieval of MEF2A-derived circRNAs. (A) Myogenic regulate factors and myocyte enhancer factor 2 family produced circRNA scanning from the circAtlas database. (B) CircMEF2As were identified from the sequencing data in the SRA database with accession number PRJNA516545 (assembly to genome Gallus gallus-5.0). (C) CircMEF2As were identified from the sequencing data in the BIG Data Center with accession number CRA002573 (assembly to genome Gallus gallus-5.0). (D) CircMEF2As were identified from the sequencing data in the GEO database with accession number GSE89355 (assembly to genome Gallus gallus-4.0). Data were displayed as mean ± SEM.
(TIF)

S2 Fig Evolutionary analysis of circMEF2As across multiple species. (A) Source information and basic characteristics of circMEF2A1 in 6 species including humans, macaque, mice, rats, pigs, and chickens. (B) Source information and basic characteristics of circMEF2A2 in 6 species. (C-E) Neighbor-Joining tree analysis of MEF2A, circMEF2A1 and circMEF2A2 in 6 species performed by MEGA7 software; Rats and mice are clustered together, humans and macaques are clustered together, pigs are in a separate cluster, and chickens are also in a separate cluster but are the furthest away from the other five species. (F) Phylogenetic tree of the 6 species, data were downloaded from the NCBI database; the results of the cluster analysis are basically similar to (C-E). (G) Species differentiation time of chicken and mouse analyzed by TimeTree database.
(TIF)

S3 Fig Detection of the knockdown efficiency of different siRNAs. (A) Knockdown efficiency of three siRNAs against circMEF2A1 analyzed by qRT-PCR, and siRNA-3 was chosen for further analysis and named si-circMEF2A1 in the main documents, n = 3. (B) Knockdown efficiency of three siRNAs against PPP3CA analyzed by qRT-PCR, siRNA-2 was chosen for further analysis and named si-PPP3CA in the main documents, n = 3. (C) Knockdown efficiency of three siRNAs against circMEF2A2 analyzed by qRT-PCR, siRNA-2 was chosen for further analysis and named si-circMEF2A2 in the main documents, n = 3. (D) Knockdown efficiency of three siRNAs against SLIT3 analyzed by qRT-PCR, siRNA-3 was chosen for further analysis and named si-SLIT3 in the main documents, n = 3. (E) Knockdown efficiency of three siRNAs against ROBO2 analyzed by qRT-PCR, siRNA-3 was chosen for further analysis and named si-ROBO2 in the main documents, n = 3. (F) Knockdown efficiency of three siRNAs against MEF2A analyzed by qRT-PCR, siRNA-2 was chosen for further analysis and named si-MEF2A in the main documents, n = 3. (G) Knockdown efficiency of two siRNAs against mmu-circMef2a1 analyzed by qRT-PCR, siRNA-2 was chosen for further analysis and named si-mmu-circMef2a1 in the main documents, n = 3. (H) Knockdown efficiency of two siRNAs against mmu-circMef2a2 analyzed by qRT-PCR, siRNA-1 was chosen for further analysis and named si-mmu-circMef2a2 in the main documents, n = 3. Data were displayed as mean ± SEM, independent sample *t*-test was used to analyze the statistical differences between each dataset, **P < 0.01 and *P < 0.05.
(TIF)

S4 Fig Lentivirus-mediated circMEF2A1 function investigation in vivo. (A, B) qRT-PCR analysis of circMEF2A1 in the cDNA samples generated from the breast muscles of LV-si-circMEF2A1, LV-si-NC, LV-ov-circMEF2A1, and LV-ov-NC infected chicks, n = 3. (C, D) Body weight of LV-si-circMEF2A1, LV-si-NC, LV-ov-circMEF2A1, and LV-ov-NC infected chicks, n = 6. (E, F) Breast muscle weight of LV-si-circMEF2A1, LV-si-NC, LV-ov-circMEF2A1, and LV-ov-NC infected chicks, n = 6. Data were displayed as mean ± SEM, independent sample *t*-test was used to analyze the statistical differences between each dataset, **P < 0.01 and *P < 0.05.
(TIF)

S5 Fig The subcellular localization and AGO2 protein adsorption of circMEF2As and linear MEF2A. (A)The subcellular localization of linear MEF2A and circMEF2As, n = 3. (B) The interaction enrichment of linear MEF2A and circMEF2As in AGO2 protein pull-down products, n = 3. Data were displayed as mean ± SEM, independent sample *t*-test was used to analyze the statistical differences between each dataset, **P < 0.01.
(TIF)

S6 Fig Conservative target analysis of the circMEF2A/miR-30a-3p/PPP3CA axis. (A) The interaction site of circMEF2A1 and miR-30a-3p is conserved in humans, mice, and chickens. (B) Venn analysis of miR-30a-3p target genes in TargetScan and miRDB databases. (C) The target site of miR-30a-3p on PPP3CA 3’UTR is conserved in humans, mice, and chickens.
(TIF)

S7 Fig Differentially expressed gene analysis between siRNA-NC and si-circMEF2A1 transfected SMSCs (fold change > 1.2 and *P*-value < 0.05). (A) The volcano plot of the differentially expressed genes between circMEF2A1 knockdown and negative control. (B) The heat map of myogenic marker genes and circMEF2A1/miR-30a-3p target genes in the differentially expressed genes.
(TIF)

S8 Fig Lentivirus-mediated circMEF2A2 function investigation in vivo. (A, B) qRT-PCR analysis of circMEF2A2 in the cDNA samples generated from the breast muscles of LV-si-circMEF2A2, LV-si-NC, LV-ov-circMEF2A2, and LV-ov-NC infected chicks, n = 3. (C, D) Body weight of LV-si-circMEF2A2, LV-si-NC, LV-ov-circMEF2A2, and LV-ov-NC infected chicks, n = 6. (E, F) Breast muscle weight of LV-si-circMEF2A2, LV-si-NC, LV-ov-circMEF2A2, and LV-ov-NC infected chicks, n = 6. Data were displayed as mean ± SEM, independent sample *t*-test was used to analyze the statistical differences between each dataset, **P < 0.01 and *P < 0.05.
(TIF)

S9 Fig Conservative target analysis of the circMEF2A2/miR-148a-5p/SLIT3. (A) The interaction site of circMEF2A2 and miR-148a-5p is conserved in humans, mice, and chickens. (B) Venn analysis of miR-148a-5p target genes in TargetScan and miRDB databases. (C) The target site of miR-148a-5p on SLIT3 3’UTR is conserved in humans, mice, and chickens.
(TIF)

S10 Fig SLIT3 receptor ROBO2 affects circMEF2A2 functions. (A) Western blot analysis of myogenic proteins, circMEF2A2/miR-148a-5p target proteins, and housekeeper proteins, in total proteins or nuclear proteins extracted from si-ROBO2 or siRNA-NC and ov-circMEF2A2 or ov-NC co-transfected SMSCs.
(TIF)

S11 Fig Conservative analysis of MEF2A functions. (A) qRT-PCR analysis of myogenic genes in the cDNA samples generated from si-MEF2A and siRNA-NC transfected SMSCs, n = 3. (B, C) The relative myotube area and proportion of MyHC^+^ cells of si-MEF2A and siRNA-NC transfected SMSCs were calculated by Image pro plus software, n = 3. (D) Immunofluorescence of MyHC in si-MEF2A and siRNA-NC transfected SMSCs. Scale bars: 200 μm. (E-G) The MEF2A protein binding element is downloaded from the JASPAR database. (H) Chicken MEF2A promoter containing MEF2A protein binding site and transcriptional start site was amplified by Cut & tag PCR primers and sequenced by Sanger sequencing. (I) The MEF2A protein binding site on the MEF2A promoter in humans, mice, and chickens. (J, K) The chip-seq data from the Cistrome database revealed MEF2A promoters have a strong binding signal of MEF2A protein in humans and mice. Data were displayed as mean ± SEM, independent sample *t*-test was used to analyze the statistical differences between each dataset, **P < 0.01 and *P < 0.05.
(TIF)

S12 Fig Conservative target analysis of miR-30a-3p or miR-148a-5p on MEF2A. (A) The interaction site of miR-30a-3p on MEF2A mRNA in humans, mice, and chickens. (B) The target site of miR-30a-3p on MEF2A 5’UTR is conserved in humans, mice, and chickens. (C) The interaction site of miR-148a-5p on MEF2A mRNA in humans, mice, and chickens. (D) The target site of miR-148a-5p on the MEF2A coding sequence is conserved in humans, mice, and chickens.
(TIF)

S1 Table qRT-PCR primers in this article. (XLSX)

S2 Table RNA oligonucleotides in this article. (XLSX)

S3 Table RNA FISH probes. (XLSX)

S4 Table Information of antibodies. (XLSX)

S1 Data Gene sets underlying graphs in figures. (XLSX)

S2 Data Numerical data underlying graphs in figures. (XLSX)

S3 Data The original blots and gels. (ZIP)

S4 Data The original photos of muscle samples. (ZIP)
