# Supplementary figures and images for: Evolutionary conserved circular MEF2A RNAs regulate myogenic differentiation and skeletal muscle development
Source: PLoS Genet. 2023 Sep 7;19(9):e1010923. doi: 10.1371/journal.pgen.1010923 (PMC10508632; doi:10.1371/journal.pgen.1010923)

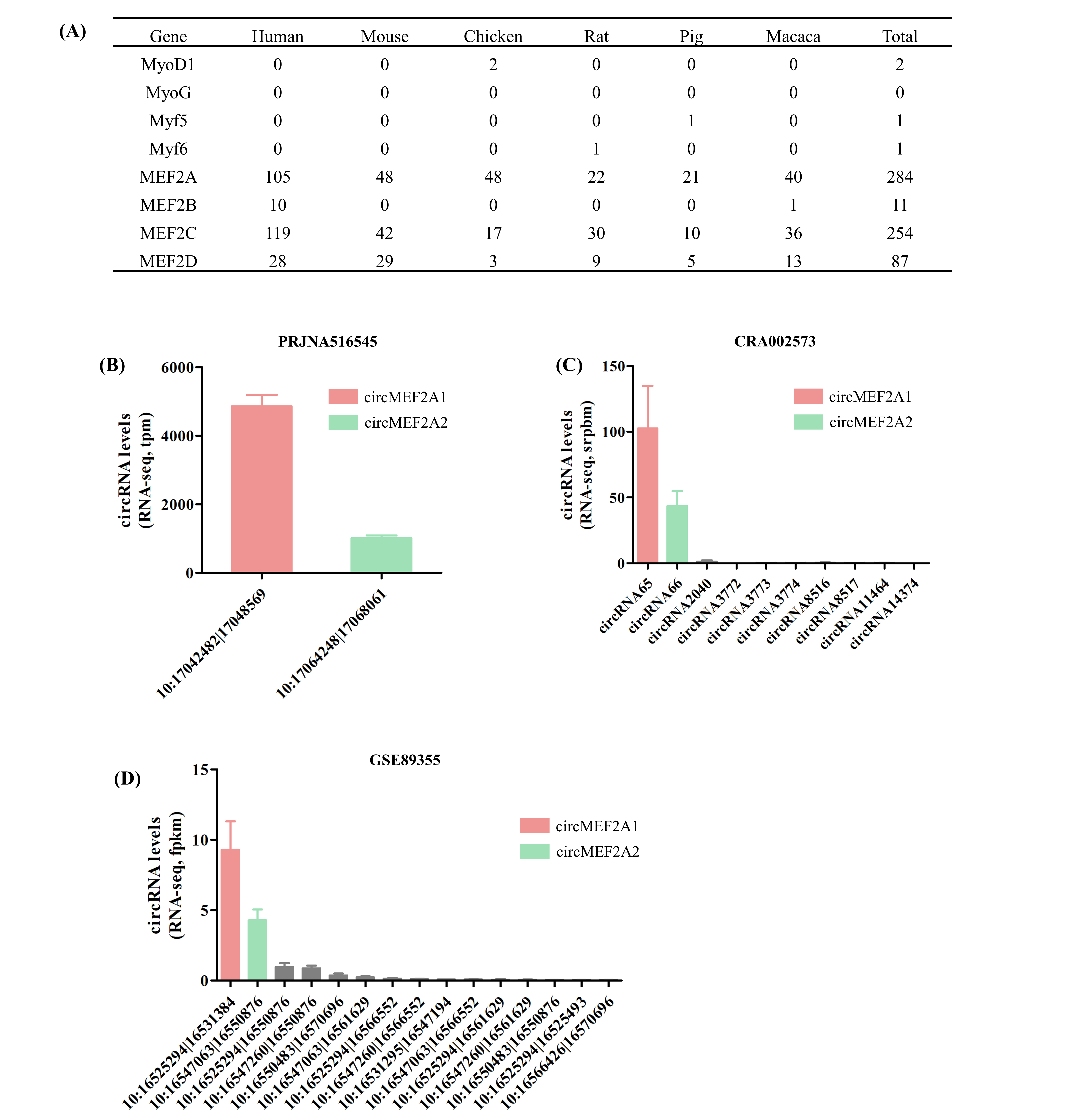

Supplement: S1 Fig — (A) Myogenic regulate factors and myocyte enhancer factor 2 family produced circRNA scanning from the circAtlas database. (B) CircMEF2As were identified from the sequencing data in the SRA database with accession number PRJNA516545 (assembly to genome Gallus gallus-5.0). (C) CircMEF2As were identified from the sequencing data in the BIG Data Center with accession number CRA002573 (assembly to genome Gallus gallus-5.0). (D) CircMEF2As were identified from the sequencing data in the GEO database with accession number GSE89355 (assembly to genome Gallus gallus-4.0). Data were displayed as mean ± SEM. (TIF) [file pgen.1010923.s001.TIF]

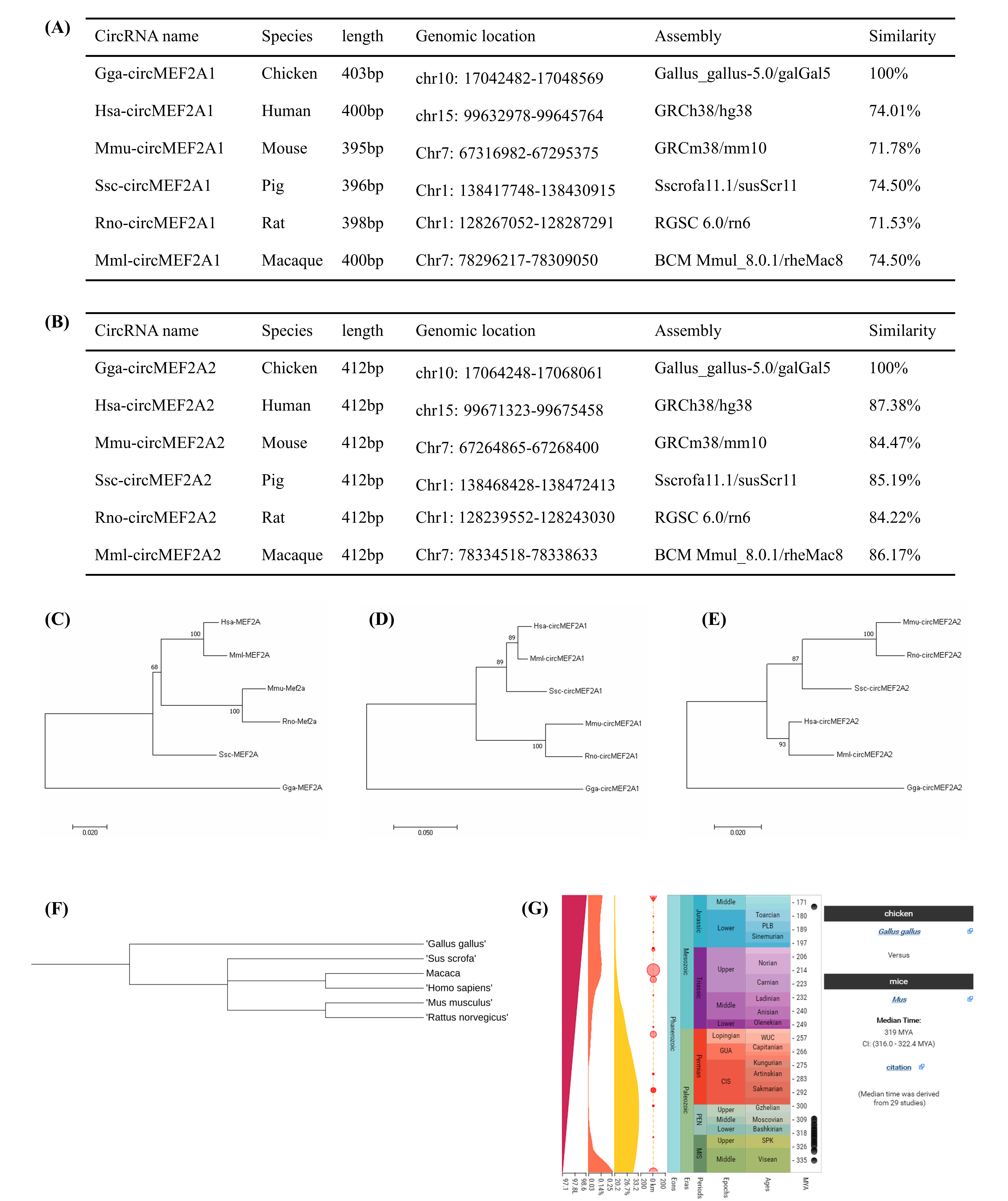

Supplement: S2 Fig — (A) Source information and basic characteristics of circMEF2A1 in 6 species including humans, macaque, mice, rats, pigs, and chickens. (B) Source information and basic characteristics of circMEF2A2 in 6 species. (C-E) Neighbor-Joining tree analysis of MEF2A, circMEF2A1 and circMEF2A2 in 6 species performed by MEGA7 software; Rats and mice are clustered together, humans and macaques are clustered together, pigs are in a separate cluster, and chickens are also in a separate cluster but are the furthest away from the other five species. (F) Phylogenetic tree of the 6 species, data were downloaded from the NCBI database; the results of the cluster analysis are basically similar to (C-E). (G) Species differentiation time of chicken and mouse analyzed by TimeTree database. (TIF) [file pgen.1010923.s002.TIF]

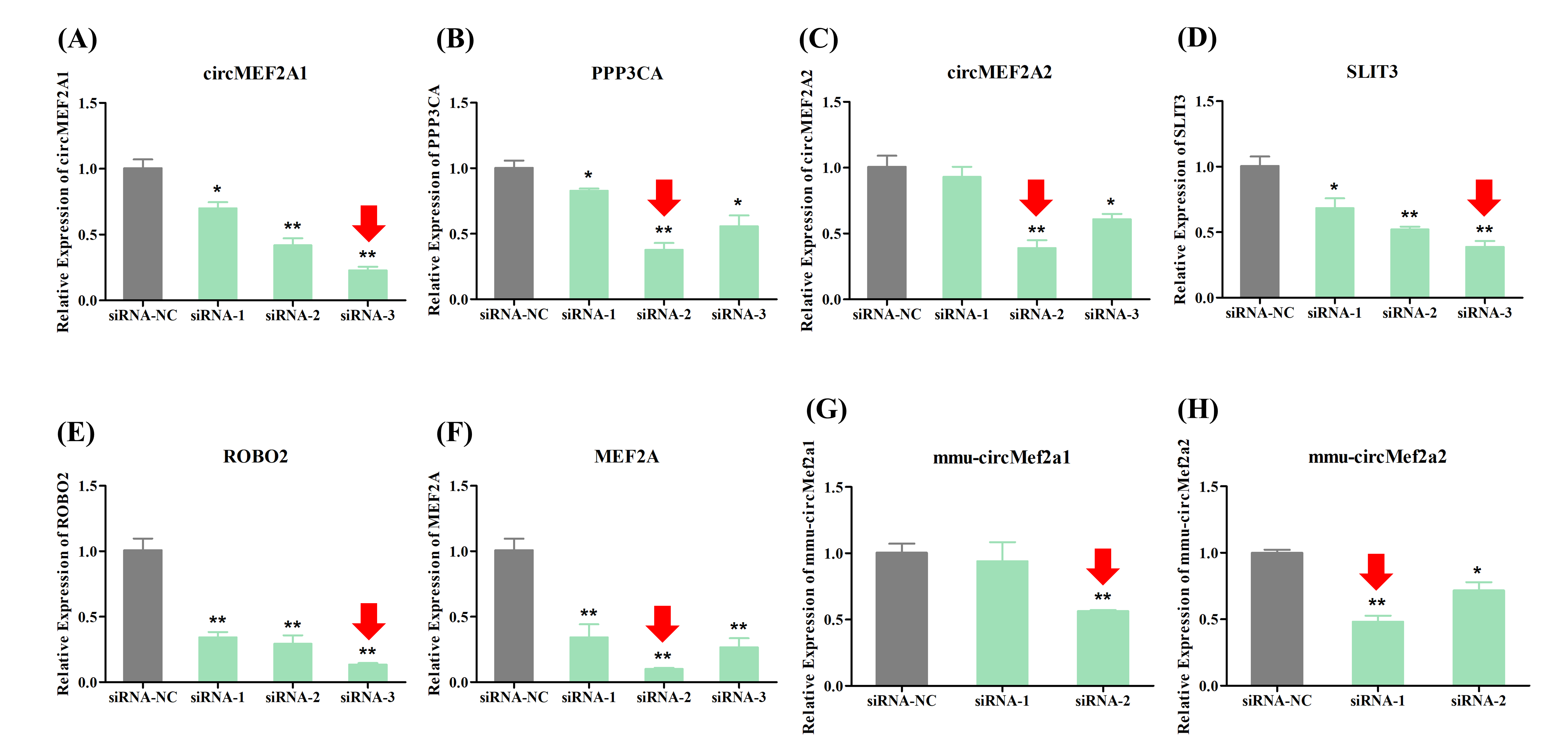

Supplement: S3 Fig — (A) Knockdown efficiency of three siRNAs against circMEF2A1 analyzed by qRT-PCR, and siRNA-3 was chosen for further analysis and named si-circMEF2A1 in the main documents, n = 3. (B) Knockdown efficiency of three siRNAs against PPP3CA analyzed by qRT-PCR, siRNA-2 was chosen for further analysis and named si-PPP3CA in the main documents, n = 3. (C) Knockdown efficiency of three siRNAs against circMEF2A2 analyzed by qRT-PCR, siRNA-2 was chosen for further analysis and named si-circMEF2A2 in the main documents, n = 3. (D) Knockdown efficiency of three siRNAs against SLIT3 analyzed by qRT-PCR, siRNA-3 was chosen for further analysis and named si-SLIT3 in the main documents, n = 3. (E) Knockdown efficiency of three siRNAs against ROBO2 analyzed by qRT-PCR, siRNA-3 was chosen for further analysis and named si-ROBO2 in the main documents, n = 3. (F) Knockdown efficiency of three siRNAs against MEF2A analyzed by qRT-PCR, siRNA-2 was chosen for further analysis and named si-MEF2A in the main documents, n = 3. (G) Knockdown efficiency of two siRNAs against mmu-circMef2a1 analyzed by qRT-PCR, siRNA-2 was chosen for further analysis and named si-mmu-circMef2a1 in the main documents, n = 3. (H) Knockdown efficiency of two siRNAs against mmu-circMef2a2 analyzed by qRT-PCR, siRNA-1 was chosen for further analysis and named si-mmu-circMef2a2 in the main documents, n = 3. Data were displayed as mean ± SEM, independent sample t-test was used to analyze the statistical differences between each dataset, **P < 0.01 and *P < 0.05. (TIF) [file pgen.1010923.s003.TIF]

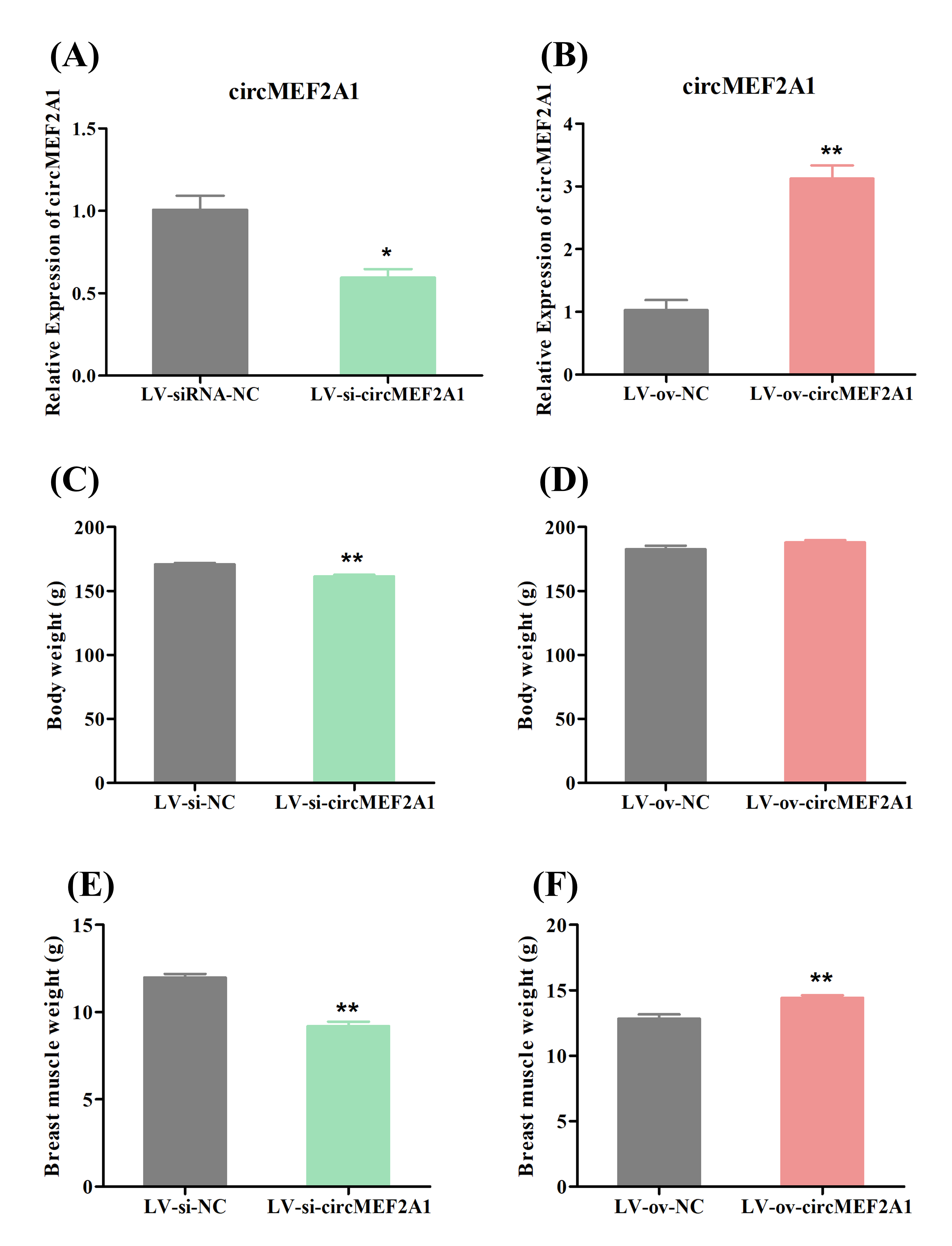

Supplement: S4 Fig — (A, B) qRT-PCR analysis of circMEF2A1 in the cDNA samples generated from the breast muscles of LV-si-circMEF2A1, LV-si-NC, LV-ov-circMEF2A1, and LV-ov-NC infected chicks, n = 3. (C, D) Body weight of LV-si-circMEF2A1, LV-si-NC, LV-ov-circMEF2A1, and LV-ov-NC infected chicks, n = 6. (E, F) Breast muscle weight of LV-si-circMEF2A1, LV-si-NC, LV-ov-circMEF2A1, and LV-ov-NC infected chicks, n = 6. Data were displayed as mean ± SEM, independent sample t-test was used to analyze the statistical differences between each dataset, **P < 0.01 and *P < 0.05. (TIF) [file pgen.1010923.s004.TIF]

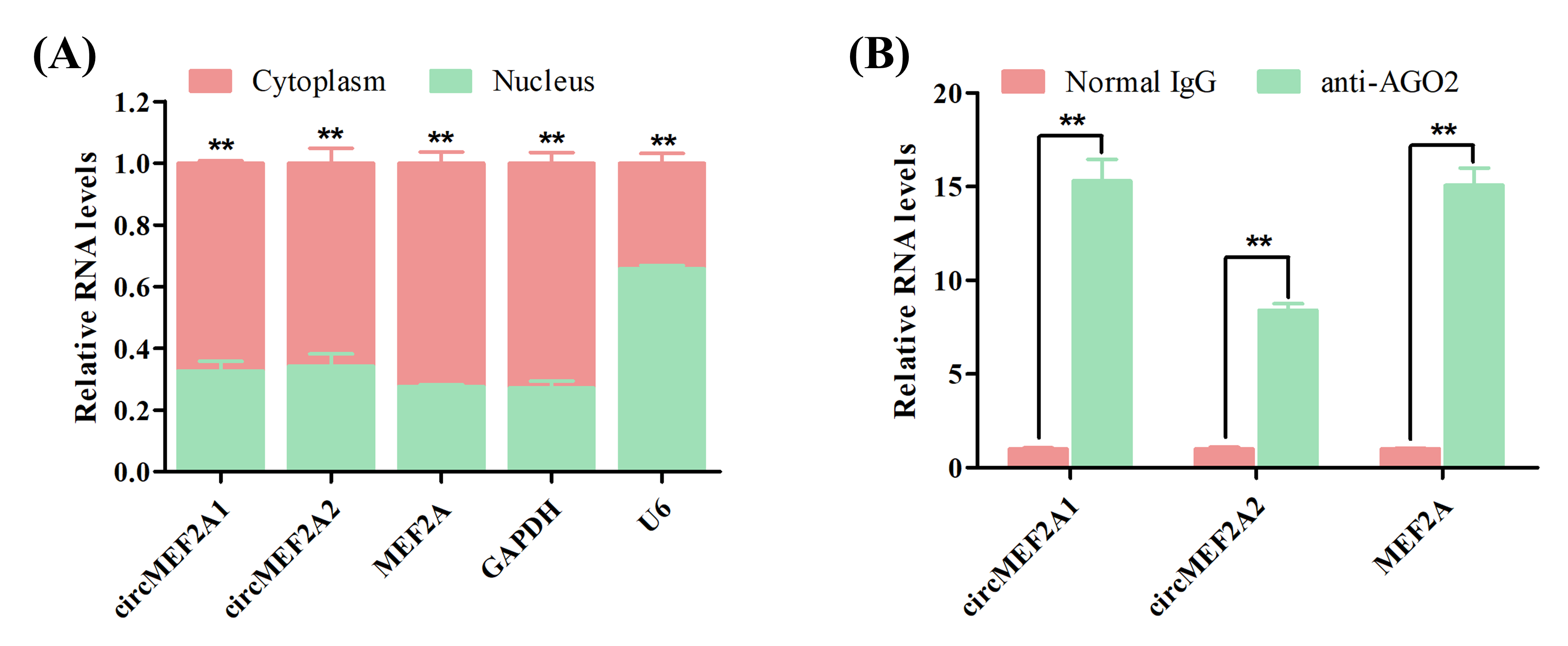

Supplement: S5 Fig — (A)The subcellular localization of linear MEF2A and circMEF2As, n = 3. (B) The interaction enrichment of linear MEF2A and circMEF2As in AGO2 protein pull-down products, n = 3. Data were displayed as mean ± SEM, independent sample t-test was used to analyze the statistical differences between each dataset, **P < 0.01. (TIF) [file pgen.1010923.s005.TIF]

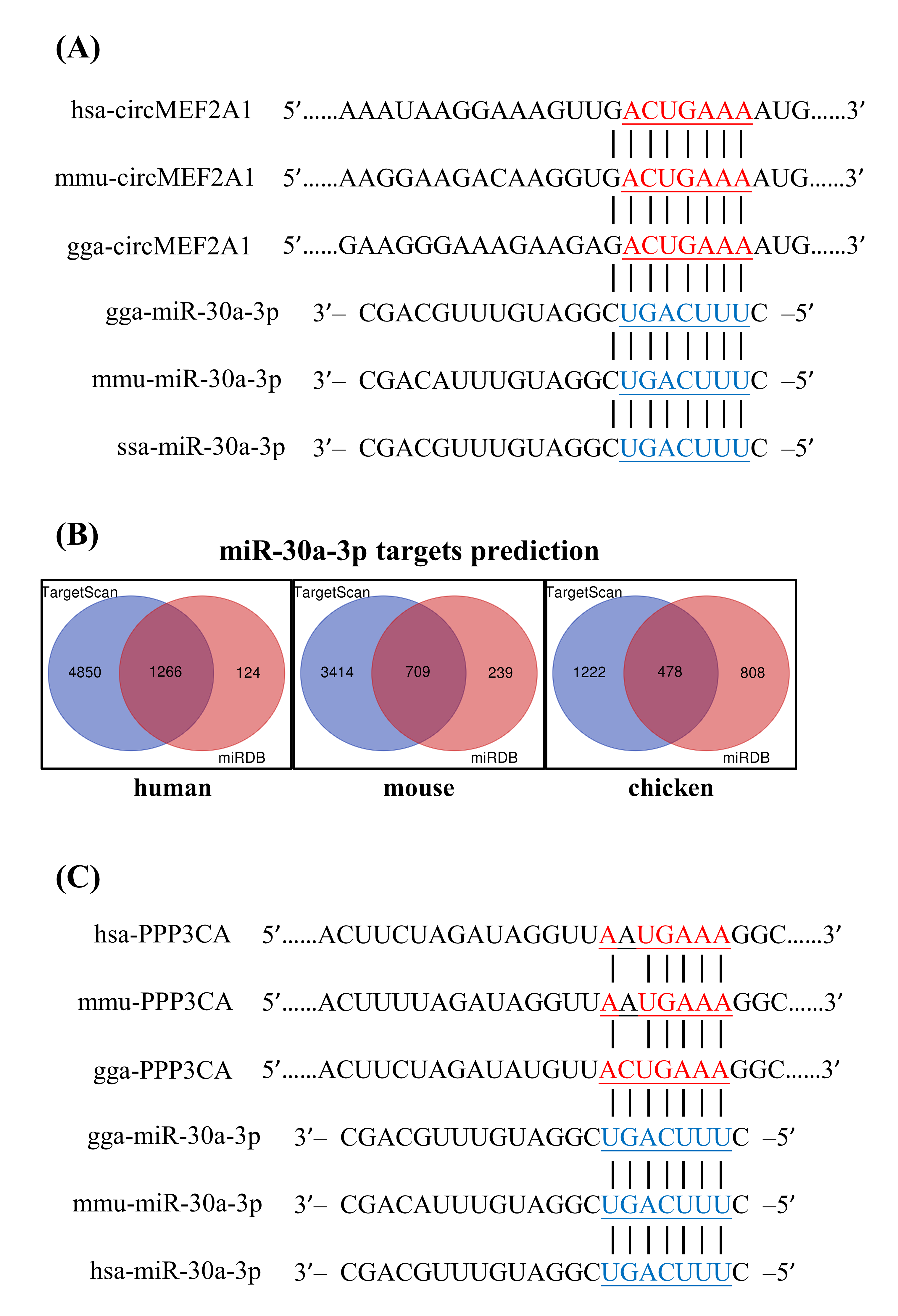

Supplement: S6 Fig — (A) The interaction site of circMEF2A1 and miR-30a-3p is conserved in humans, mice, and chickens. (B) Venn analysis of miR-30a-3p target genes in TargetScan and miRDB databases. (C) The target site of miR-30a-3p on PPP3CA 3’UTR is conserved in humans, mice, and chickens. (TIF) [file pgen.1010923.s006.TIF]

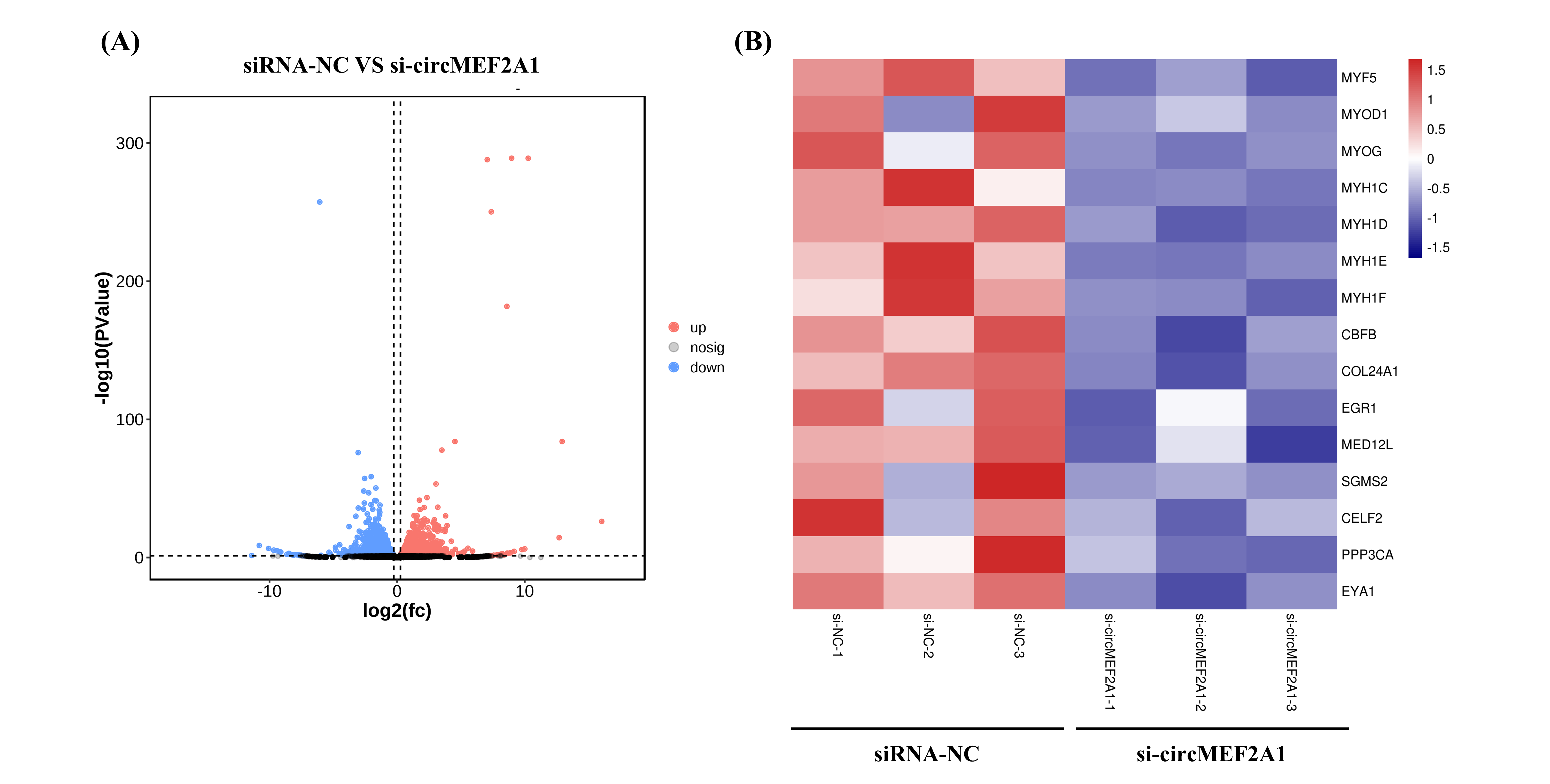

Supplement: S7 Fig — (A) The volcano plot of the differentially expressed genes between circMEF2A1 knockdown and negative control. (B) The heat map of myogenic marker genes and circMEF2A1/miR-30a-3p target genes in the differentially expressed genes. (TIF) [file pgen.1010923.s007.TIF]

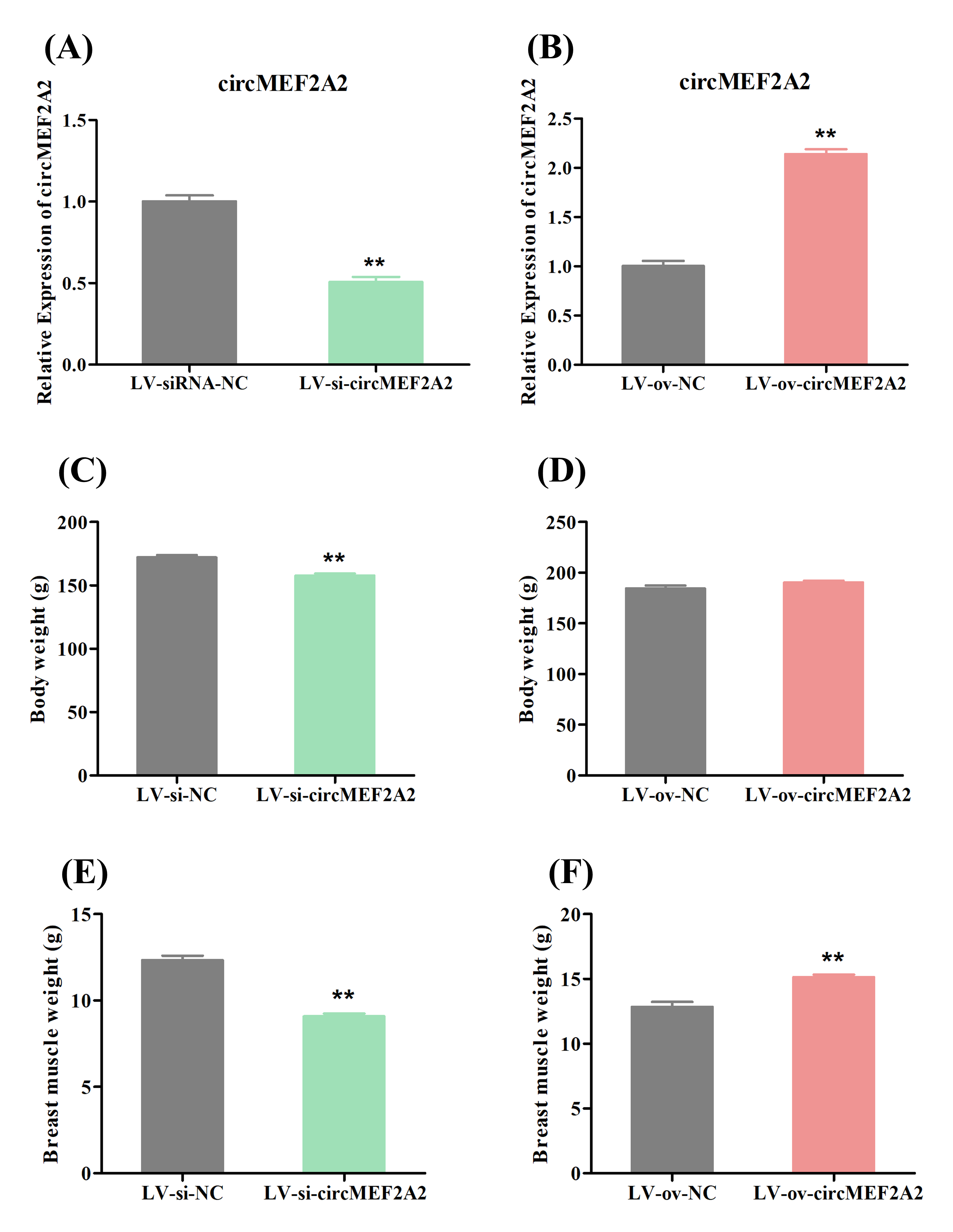

Supplement: S8 Fig — (A, B) qRT-PCR analysis of circMEF2A2 in the cDNA samples generated from the breast muscles of LV-si-circMEF2A2, LV-si-NC, LV-ov-circMEF2A2, and LV-ov-NC infected chicks, n = 3. (C, D) Body weight of LV-si-circMEF2A2, LV-si-NC, LV-ov-circMEF2A2, and LV-ov-NC infected chicks, n = 6. (E, F) Breast muscle weight of LV-si-circMEF2A2, LV-si-NC, LV-ov-circMEF2A2, and LV-ov-NC infected chicks, n = 6. Data were displayed as mean ± SEM, independent sample t-test was used to analyze the statistical differences between each dataset, **P < 0.01 and *P < 0.05. (TIF) [file pgen.1010923.s008.TIF]

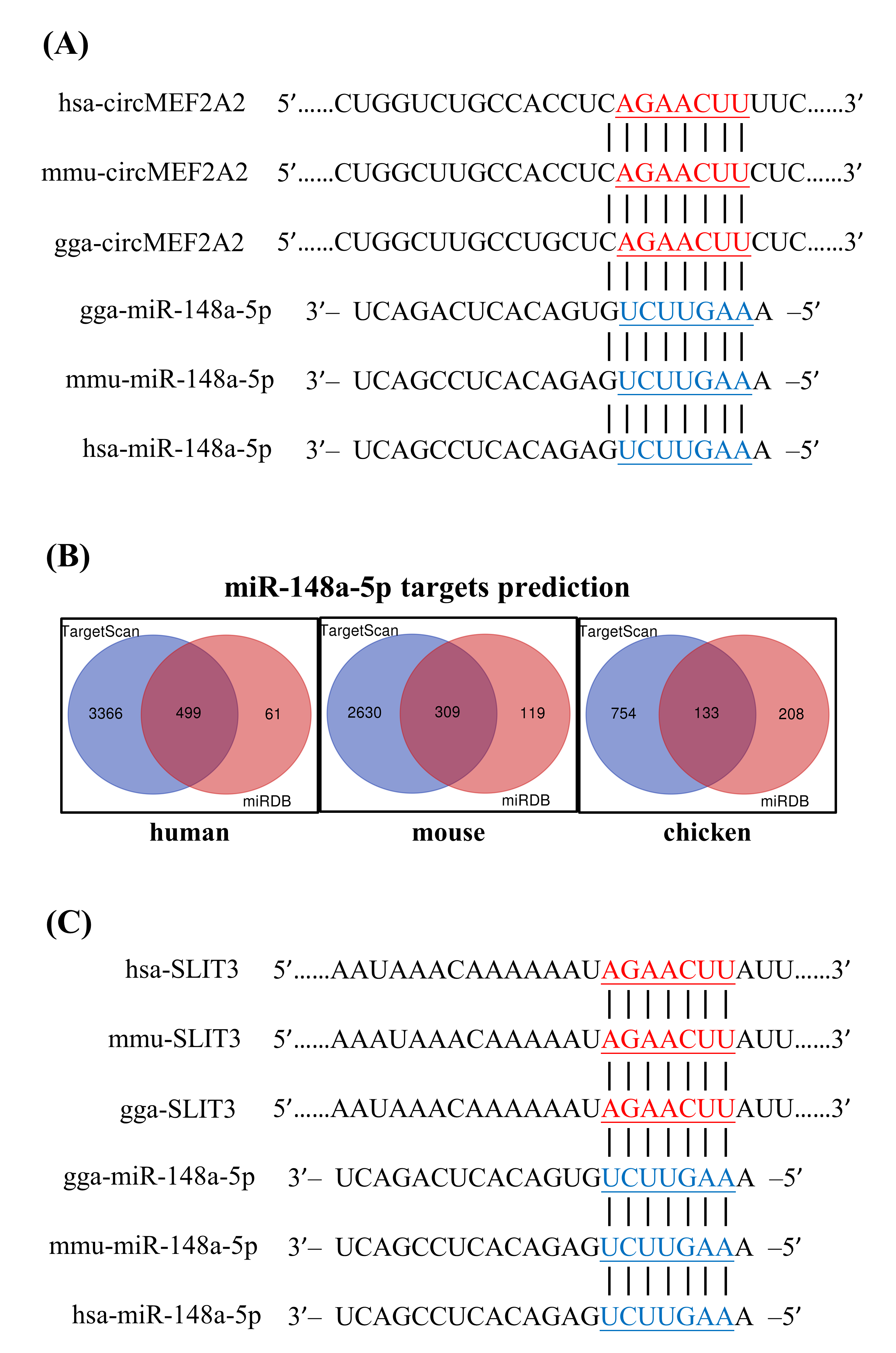

Supplement: S9 Fig — (A) The interaction site of circMEF2A2 and miR-148a-5p is conserved in humans, mice, and chickens. (B) Venn analysis of miR-148a-5p target genes in TargetScan and miRDB databases. (C) The target site of miR-148a-5p on SLIT3 3’UTR is conserved in humans, mice, and chickens. (TIF) [file pgen.1010923.s009.TIF]

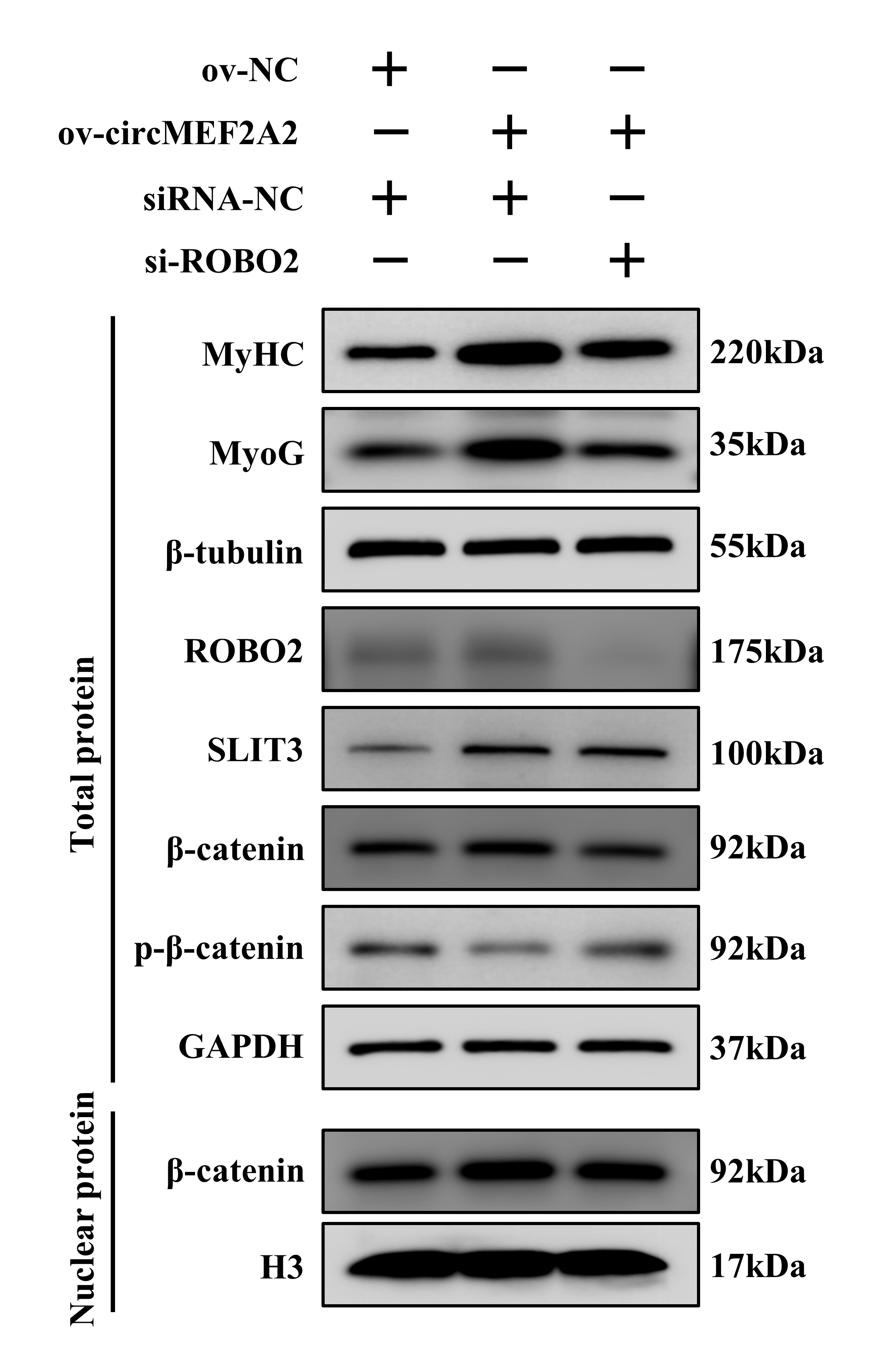

Supplement: S10 Fig — (A) Western blot analysis of myogenic proteins, circMEF2A2/miR-148a-5p target proteins, and housekeeper proteins, in total proteins or nuclear proteins extracted from si-ROBO2 or siRNA-NC and ov-circMEF2A2 or ov-NC co-transfected SMSCs. (TIF) [file pgen.1010923.s010.TIF]

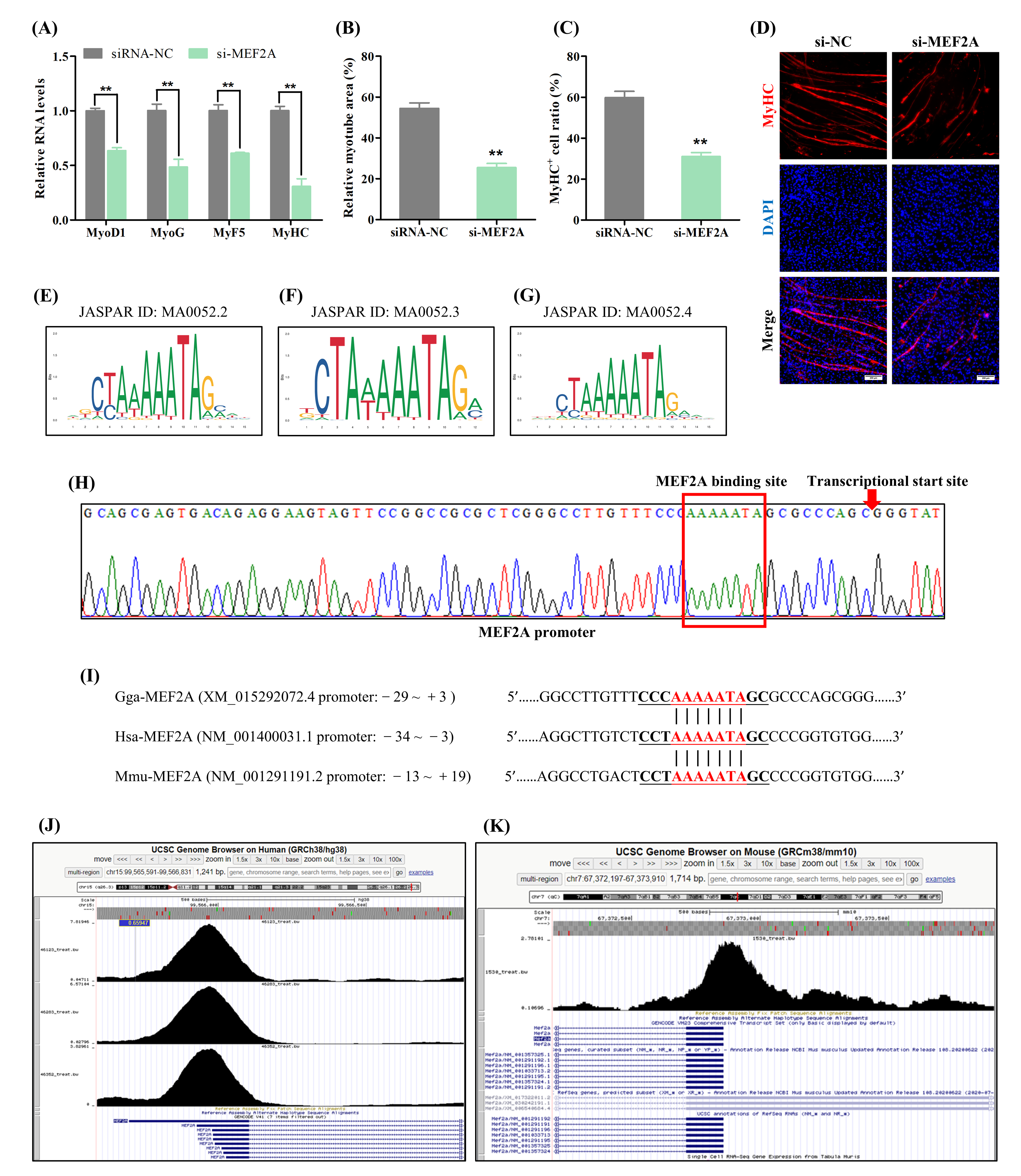

Supplement: S11 Fig — (A) qRT-PCR analysis of myogenic genes in the cDNA samples generated from si-MEF2A and siRNA-NC transfected SMSCs, n = 3. (B, C) The relative myotube area and proportion of MyHC+ cells of si-MEF2A and siRNA-NC transfected SMSCs were calculated by Image pro plus software, n = 3. (D) Immunofluorescence of MyHC in si-MEF2A and siRNA-NC transfected SMSCs. Scale bars: 200 μm. (E-G) The MEF2A protein binding element is downloaded from the JASPAR database. (H) Chicken MEF2A promoter containing MEF2A protein binding site and transcriptional start site was amplified by Cut & tag PCR primers and sequenced by Sanger sequencing. (I) The MEF2A protein binding site on the MEF2A promoter in humans, mice, and chickens. (J, K) The chip-seq data from the Cistrome database revealed MEF2A promoters have a strong binding signal of MEF2A protein in humans and mice. Data were displayed as mean ± SEM, independent sample t-test was used to analyze the statistical differences between each dataset, **P < 0.01 and *P < 0.05. (TIF) [file pgen.1010923.s011.tif]

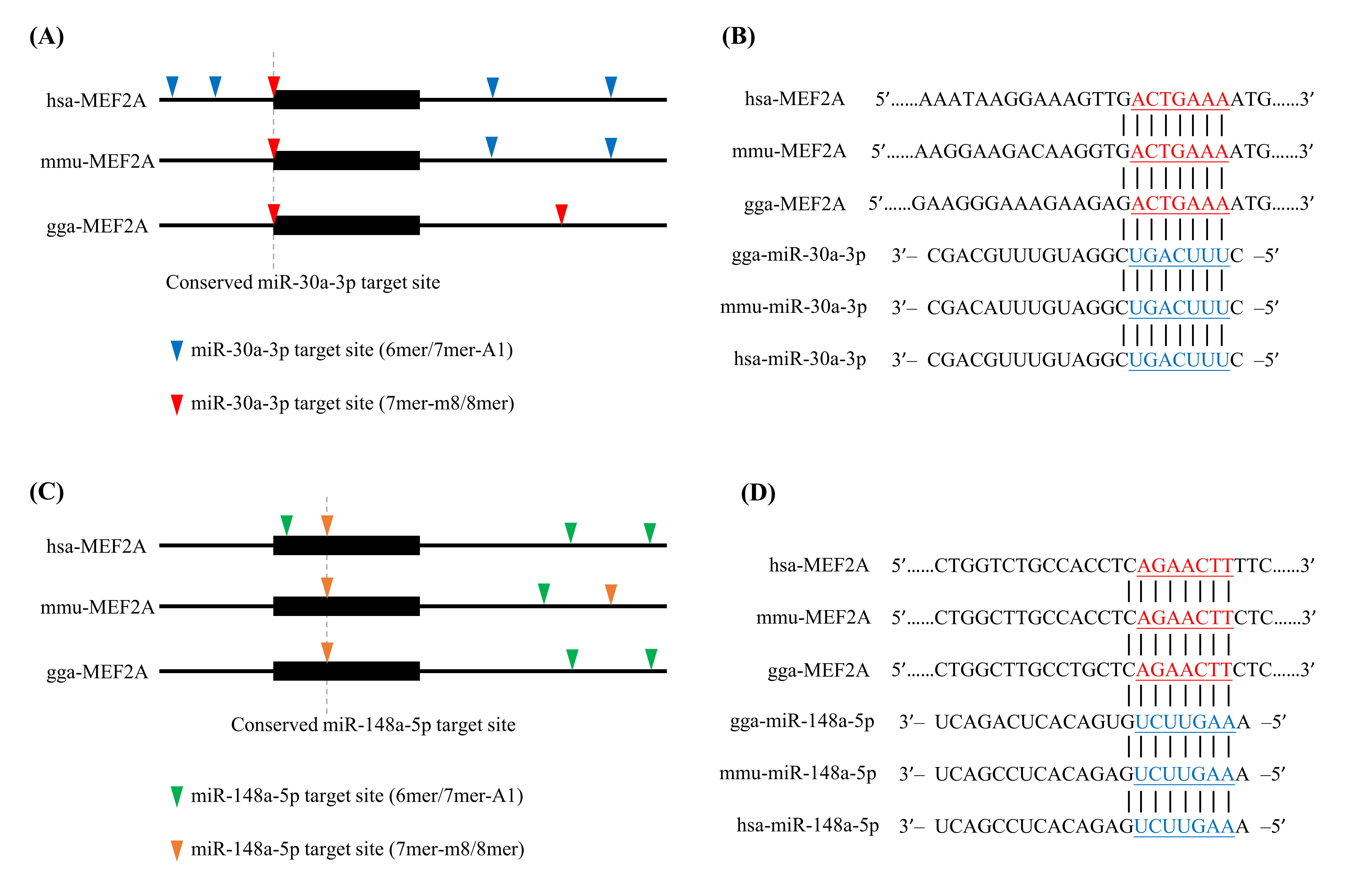

Supplement: S12 Fig — (A) The interaction site of miR-30a-3p on MEF2A mRNA in humans, mice, and chickens. (B) The target site of miR-30a-3p on MEF2A 5’UTR is conserved in humans, mice, and chickens. (C) The interaction site of miR-148a-5p on MEF2A mRNA in humans, mice, and chickens. (D) The target site of miR-148a-5p on the MEF2A coding sequence is conserved in humans, mice, and chickens. (TIF) [file pgen.1010923.s012.TIF]

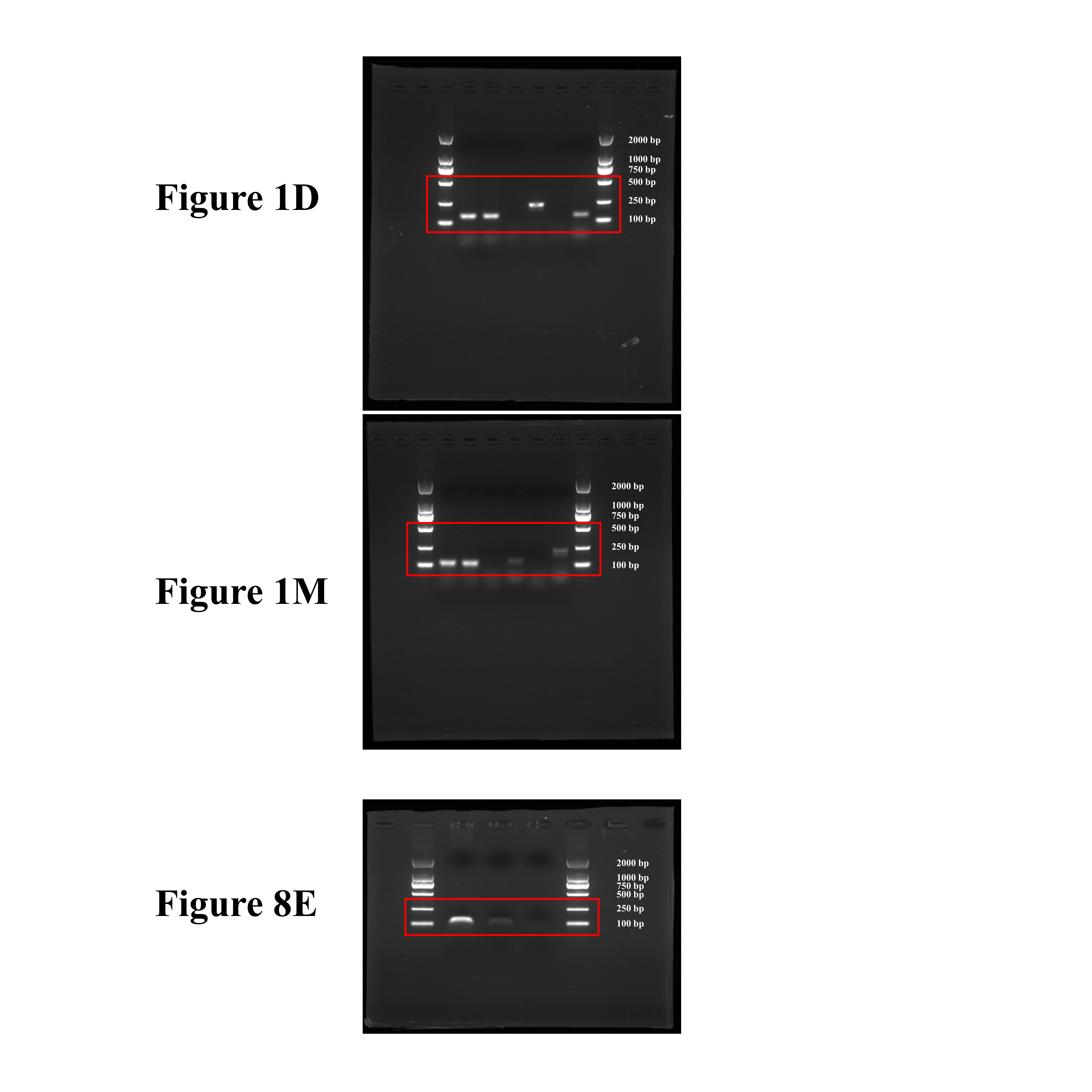

Supplement: S3 Data — (ZIP) [file pgen.1010923.s019.zip › S3 Data. The original blots and gels/The original gels.TIF]

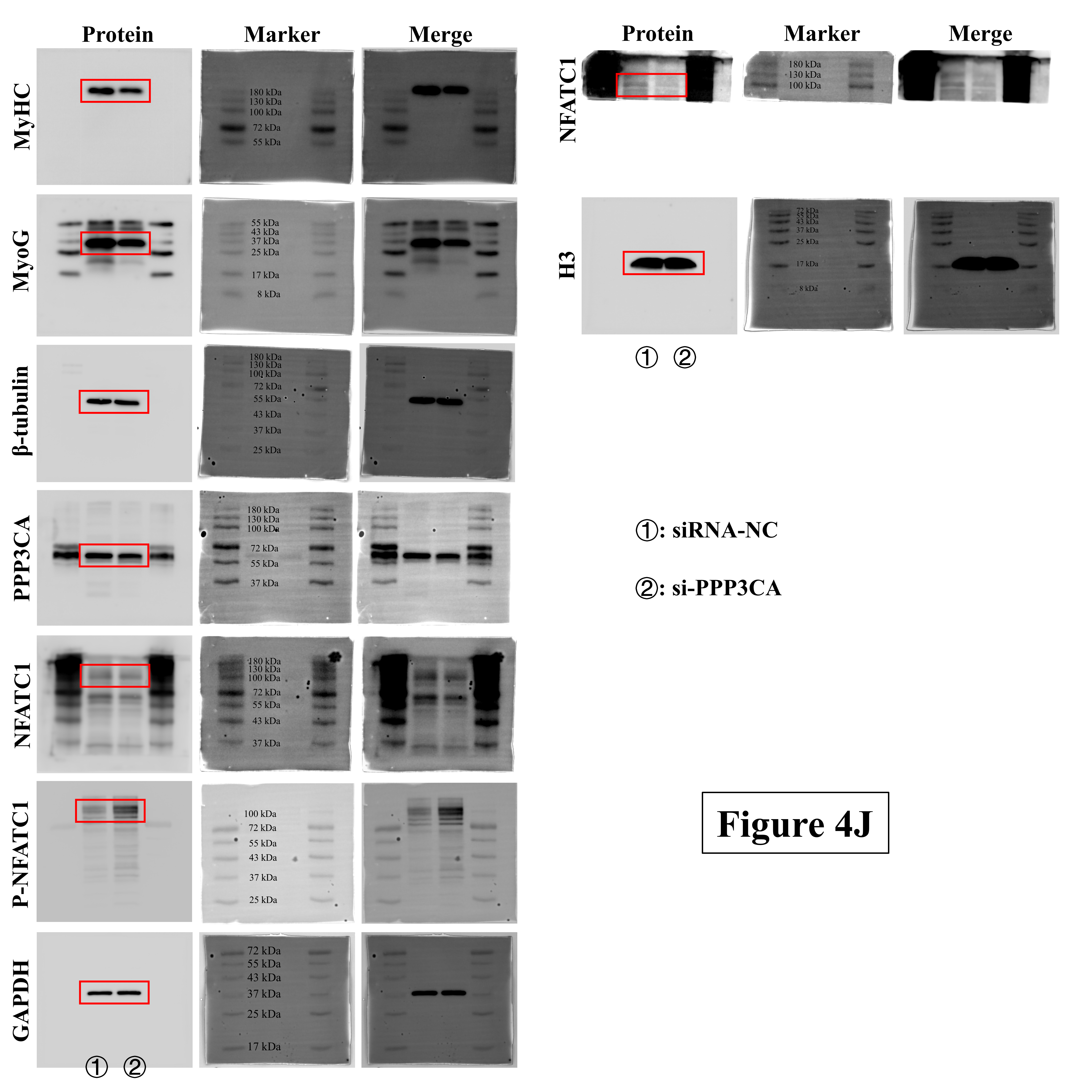

Supplement: S3 Data — (ZIP) [file pgen.1010923.s019.zip › S3 Data. The original blots and gels/The original western blot for figure 4J.TIF]

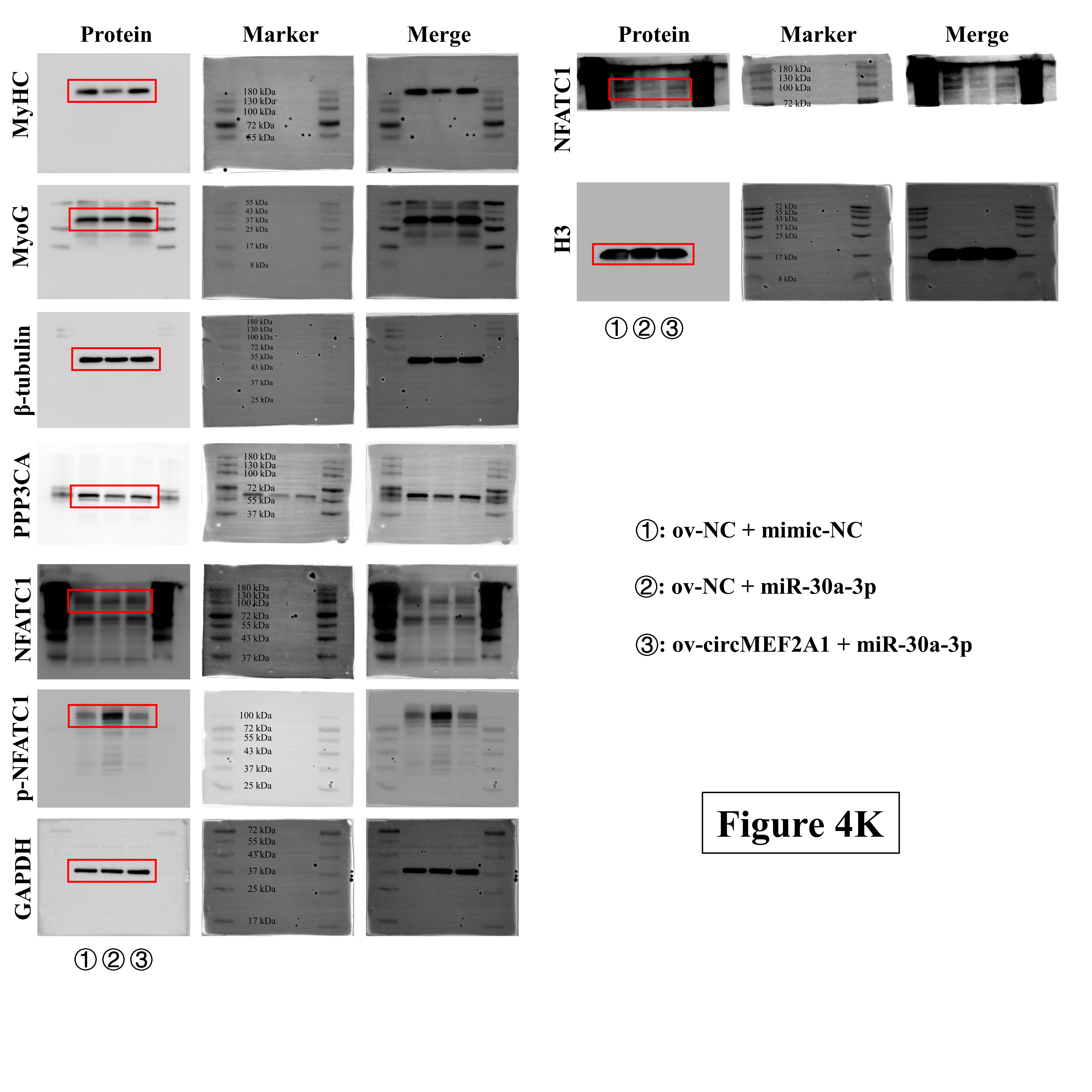

Supplement: S3 Data — (ZIP) [file pgen.1010923.s019.zip › S3 Data. The original blots and gels/The original western blot for figure 4K.TIF]

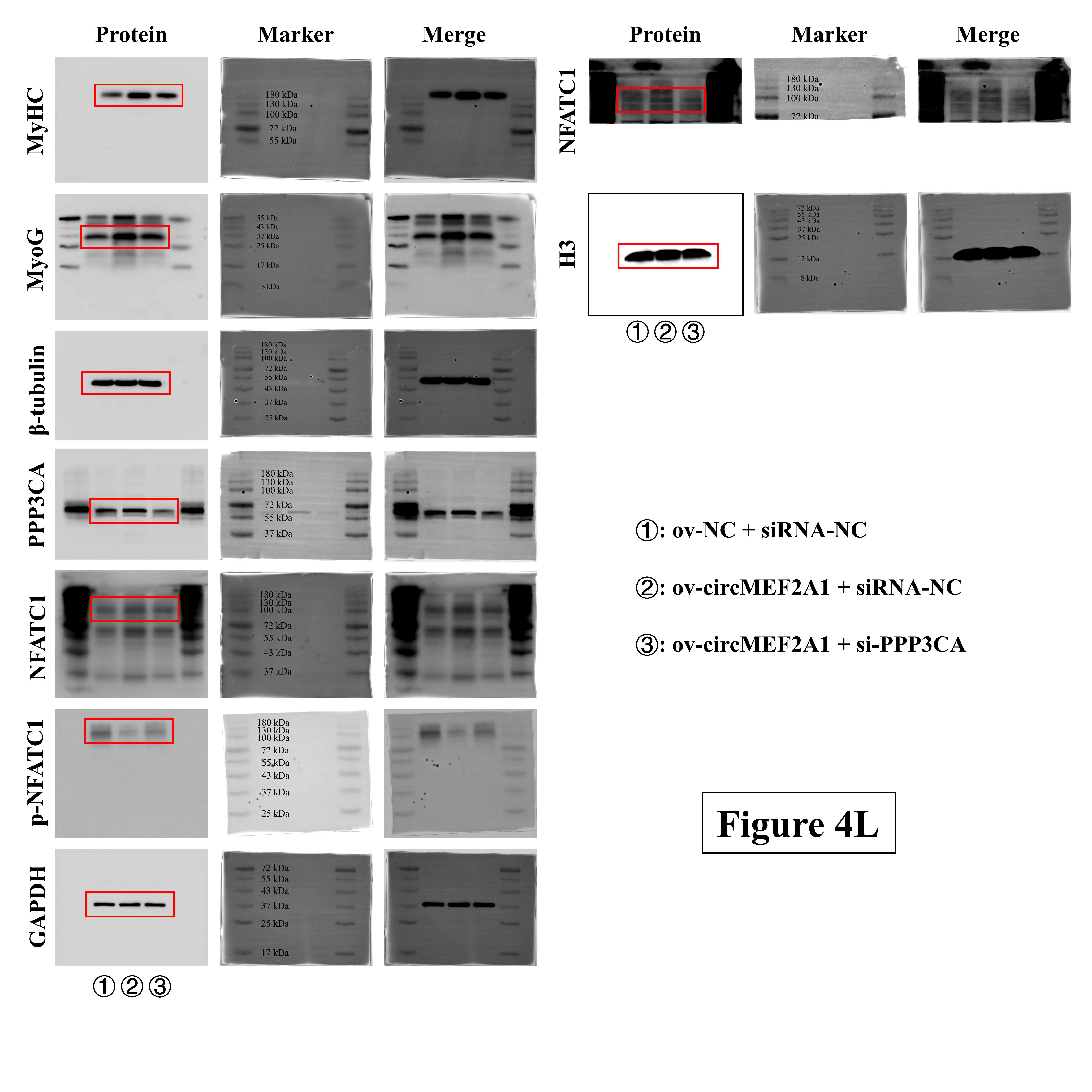

Supplement: S3 Data — (ZIP) [file pgen.1010923.s019.zip › S3 Data. The original blots and gels/The original western blot for figure 4L.TIF]

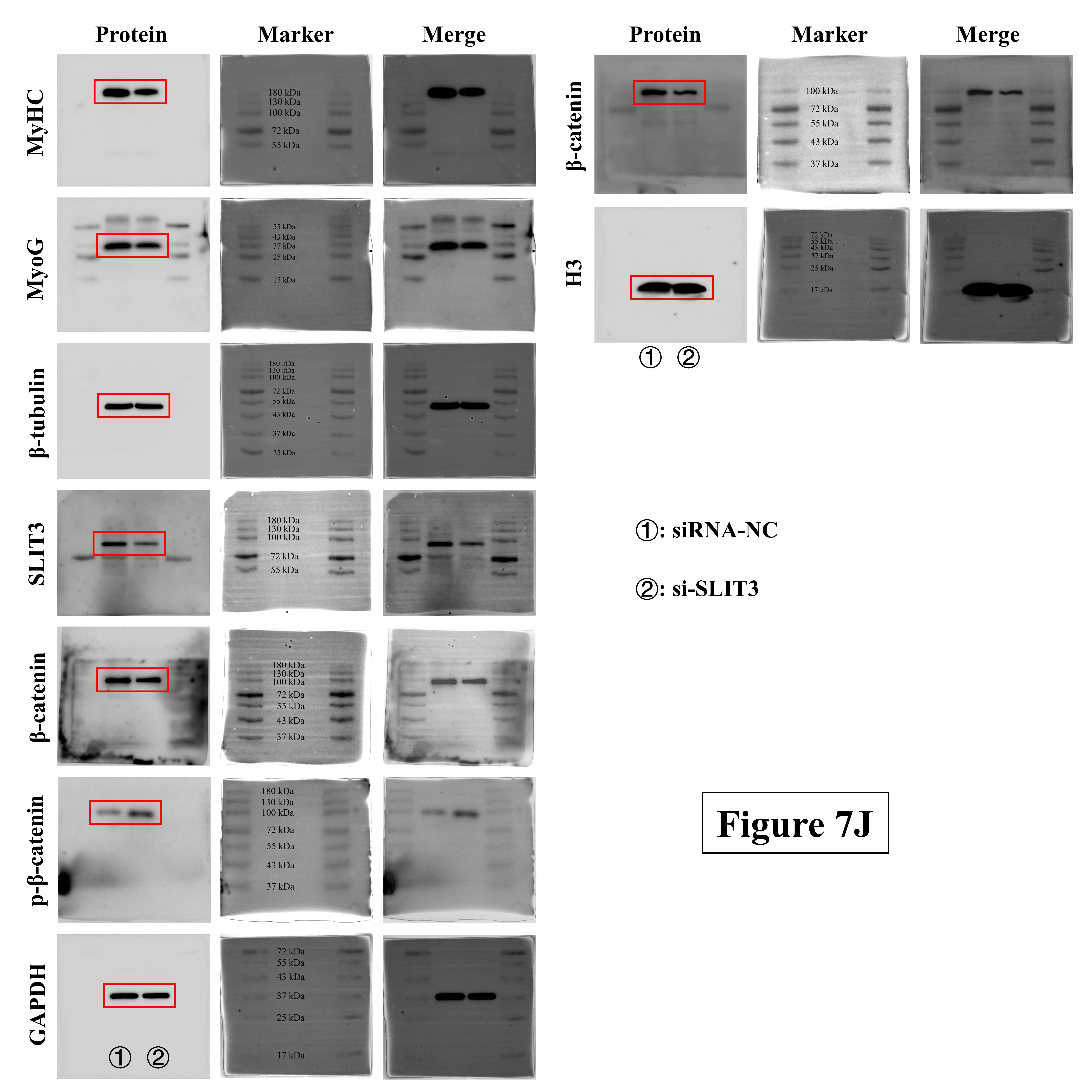

Supplement: S3 Data — (ZIP) [file pgen.1010923.s019.zip › S3 Data. The original blots and gels/The original western blot for figure 7J.TIF]

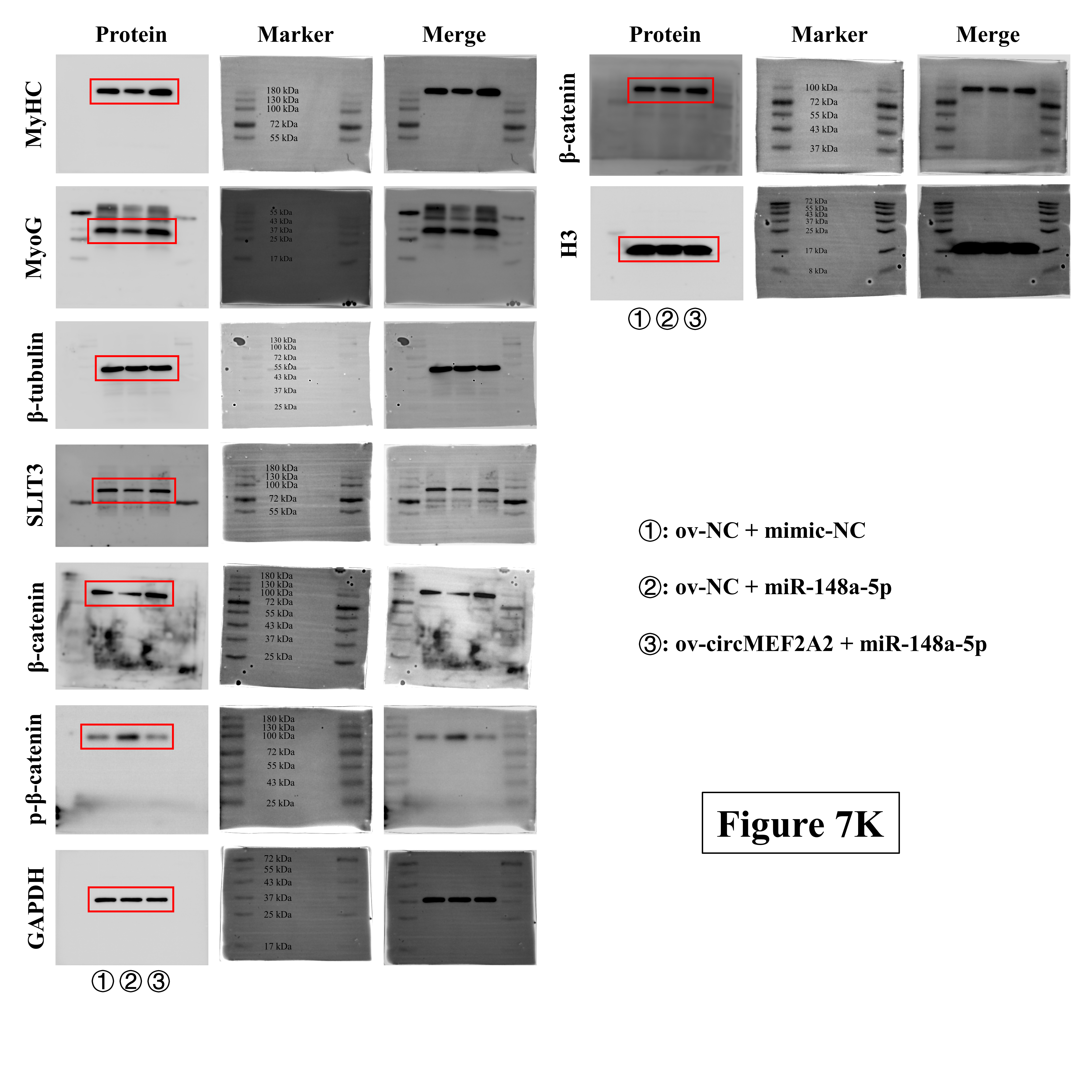

Supplement: S3 Data — (ZIP) [file pgen.1010923.s019.zip › S3 Data. The original blots and gels/The original western blot for figure 7K.TIF]

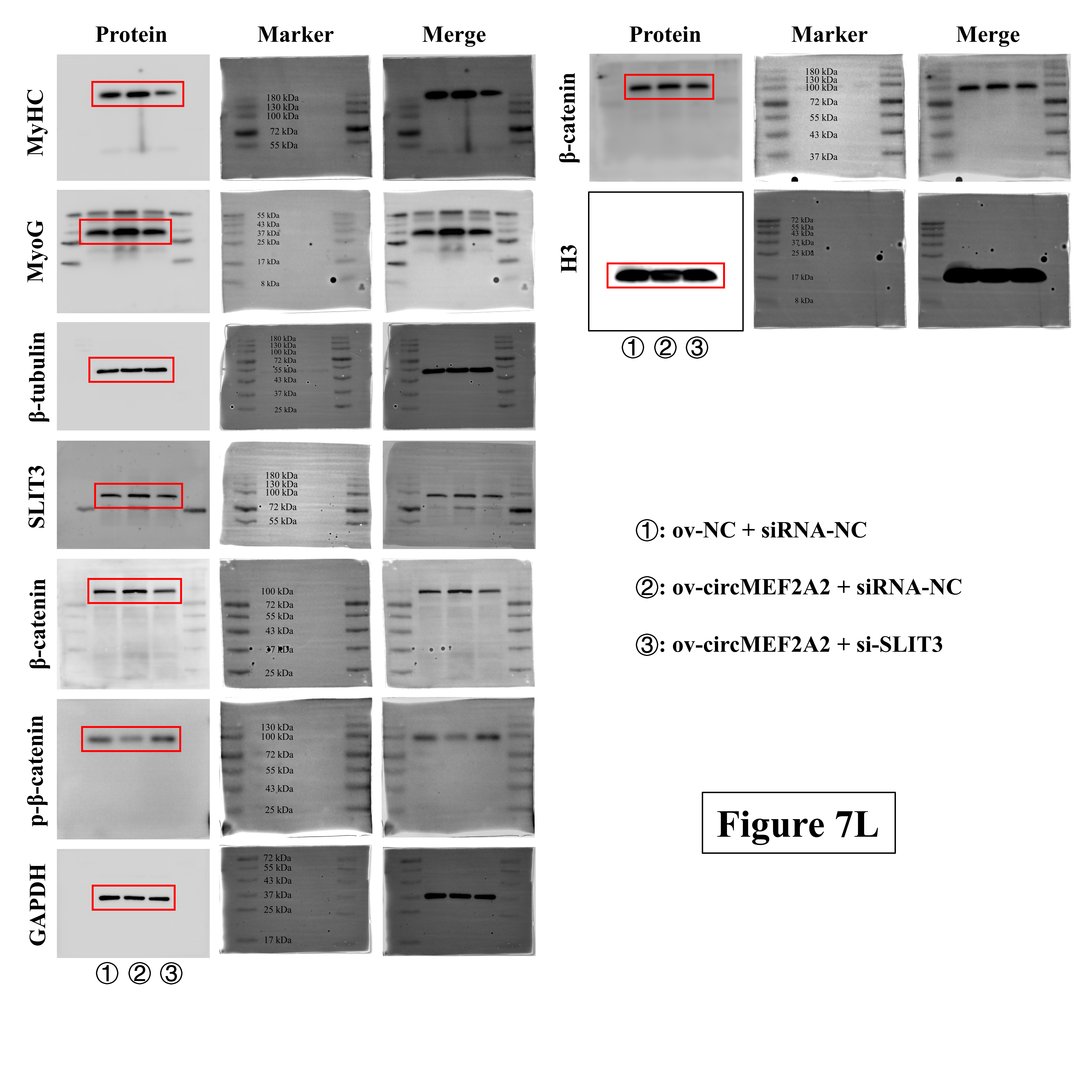

Supplement: S3 Data — (ZIP) [file pgen.1010923.s019.zip › S3 Data. The original blots and gels/The original western blot for figure 7L.TIF]

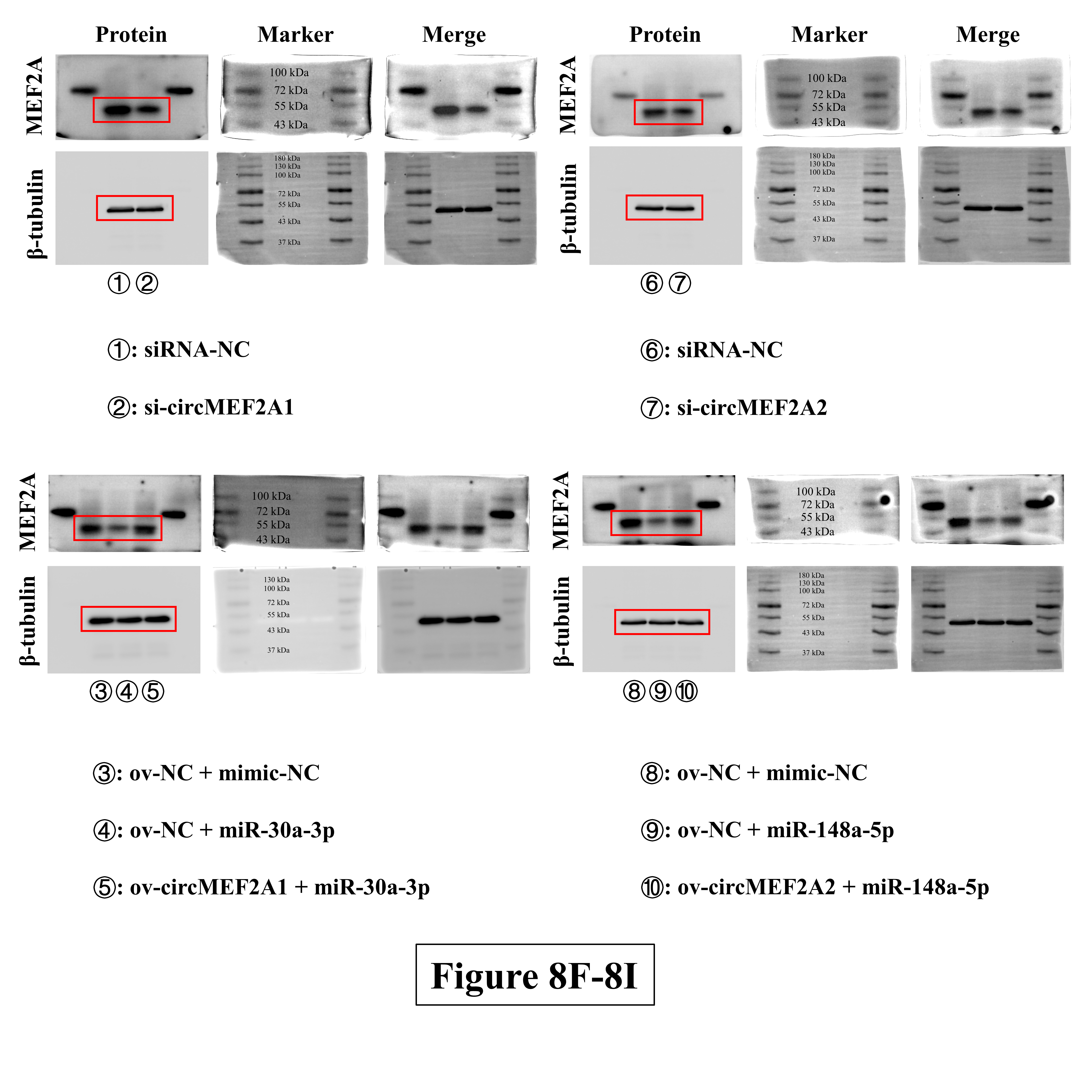

Supplement: S3 Data — (ZIP) [file pgen.1010923.s019.zip › S3 Data. The original blots and gels/The original western blot for figure 8F-8I.TIF]

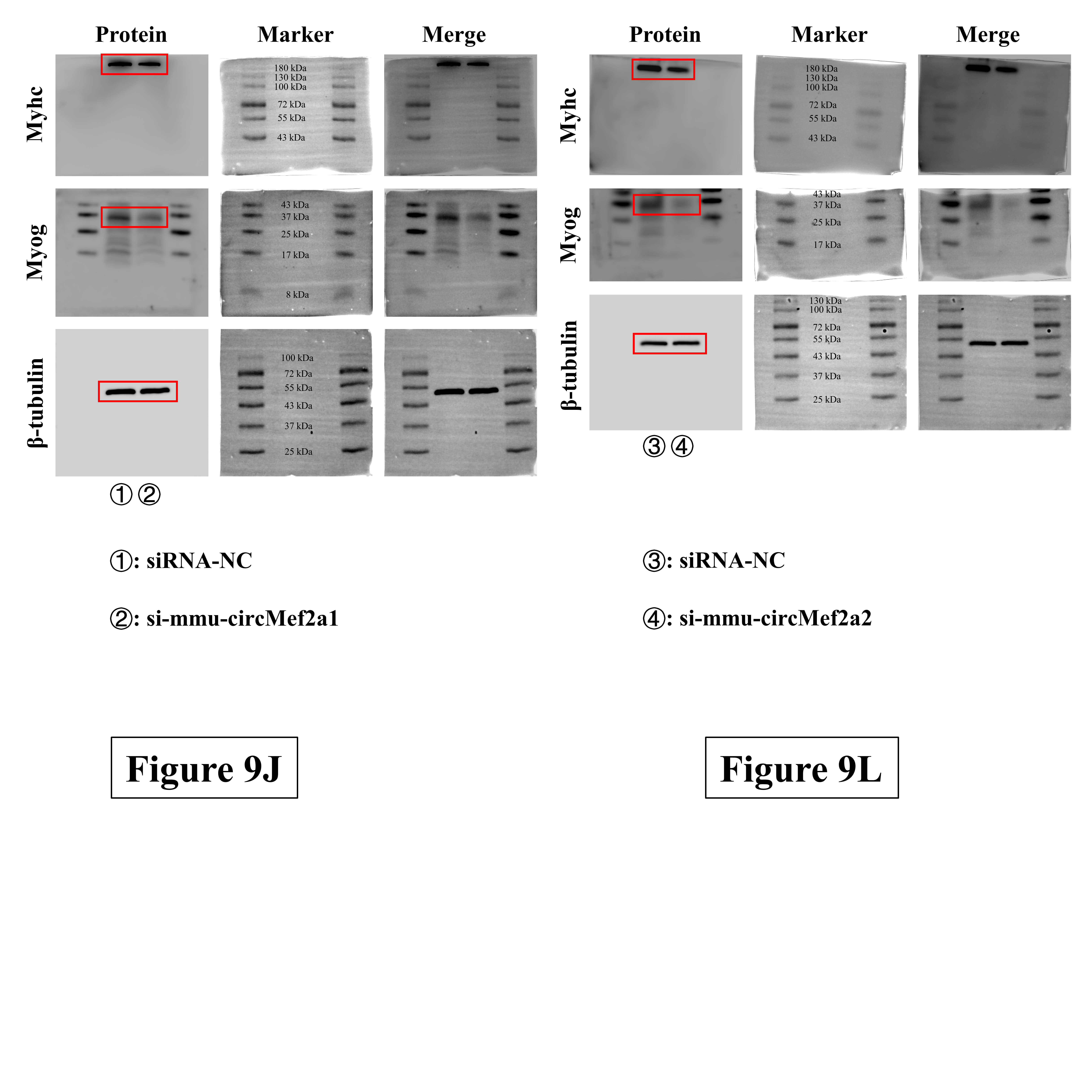

Supplement: S3 Data — (ZIP) [file pgen.1010923.s019.zip › S3 Data. The original blots and gels/The original western blot for figure 9J and 9L.TIF]

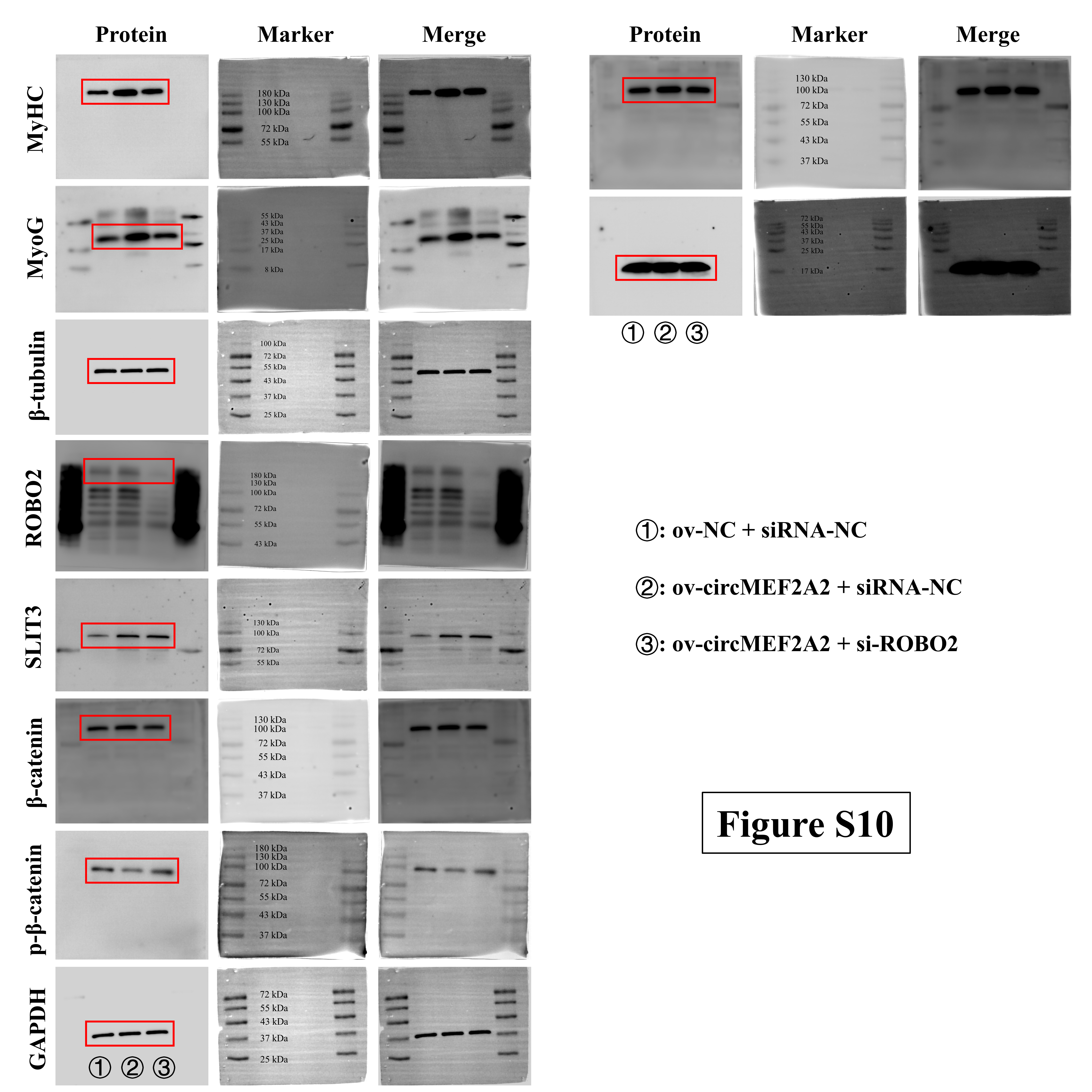

Supplement: S3 Data — (ZIP) [file pgen.1010923.s019.zip › S3 Data. The original blots and gels/The original western blot for figure S10.TIF]

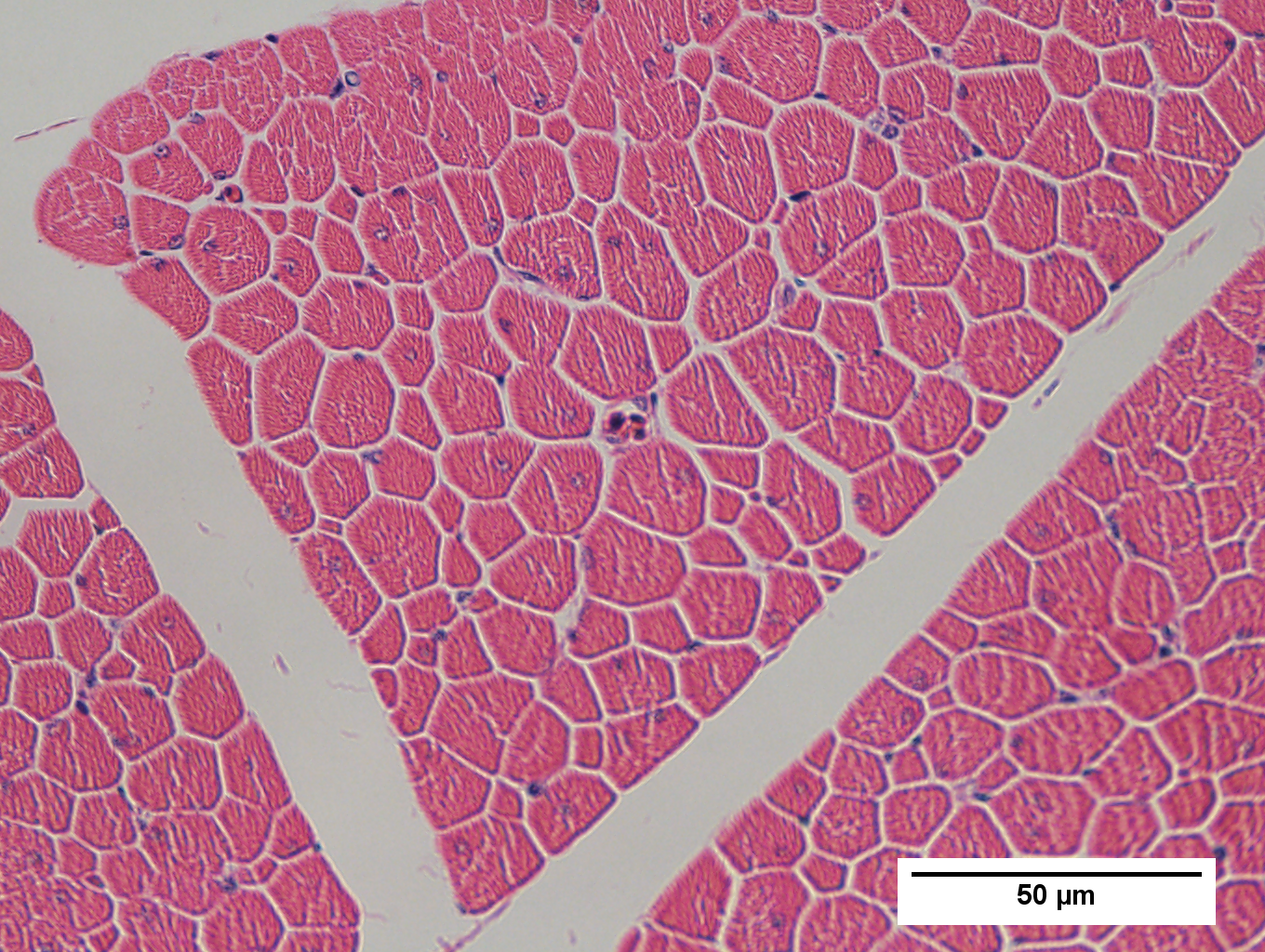

Supplement: S4 Data — (ZIP) [file pgen.1010923.s020.zip › S4 Data. The original photos of muscle samples/Cross section of muscle samples/LV-ov-circMEF2A1-NC.tif]

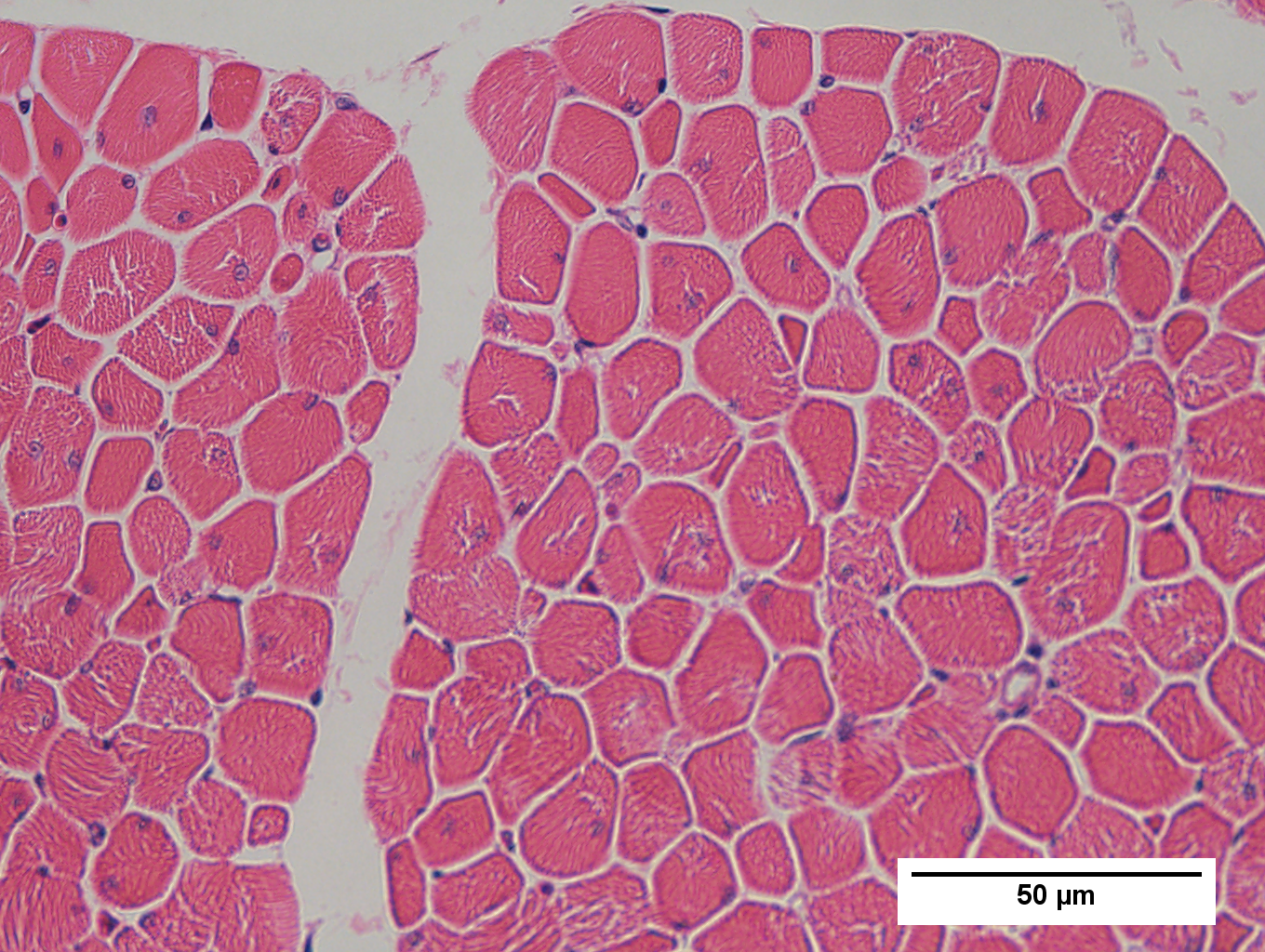

Supplement: S4 Data — (ZIP) [file pgen.1010923.s020.zip › S4 Data. The original photos of muscle samples/Cross section of muscle samples/LV-ov-circMEF2A1.tif]

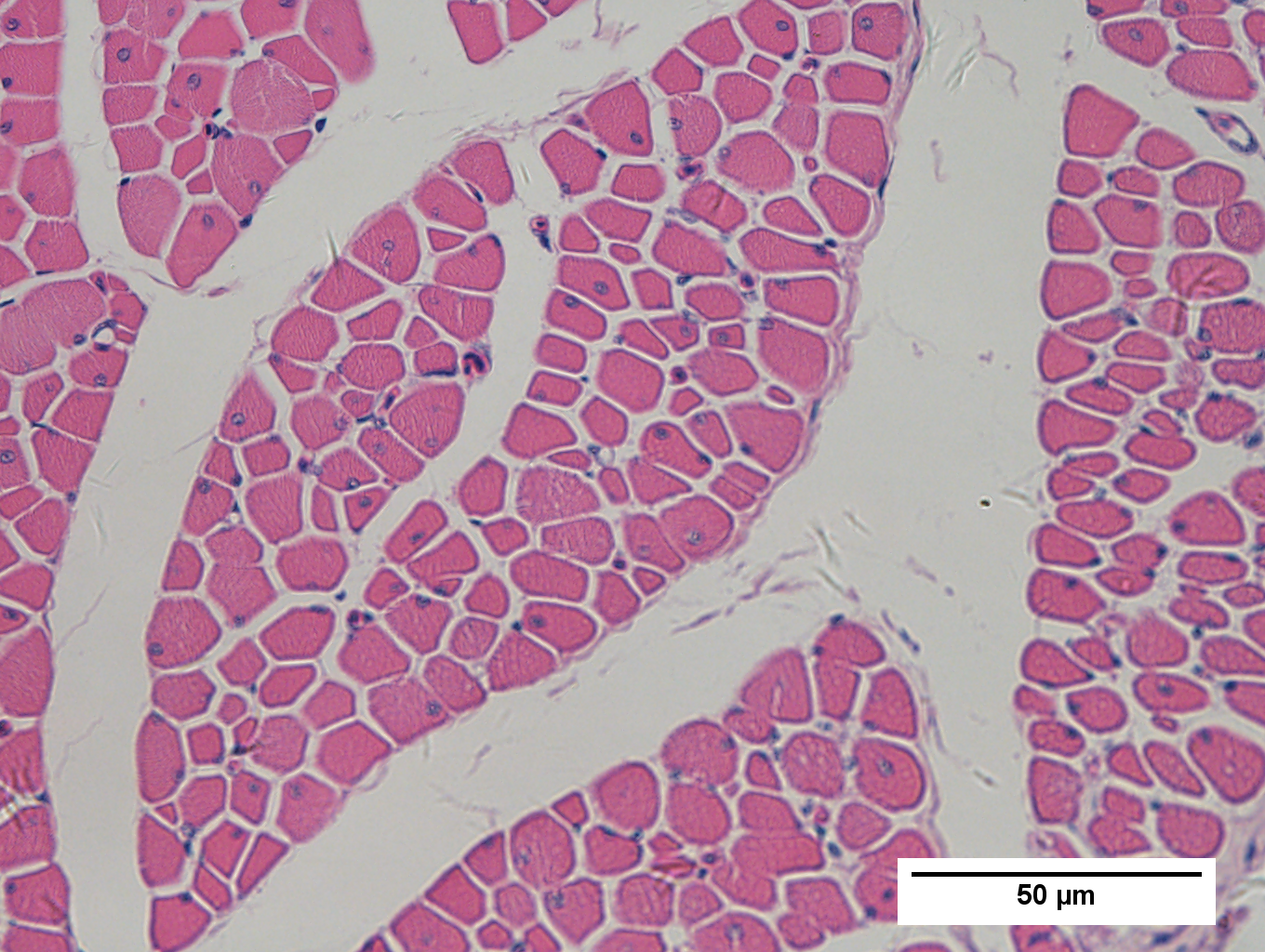

Supplement: S4 Data — (ZIP) [file pgen.1010923.s020.zip › S4 Data. The original photos of muscle samples/Cross section of muscle samples/LV-ov-circMEF2A2-NC.tif]

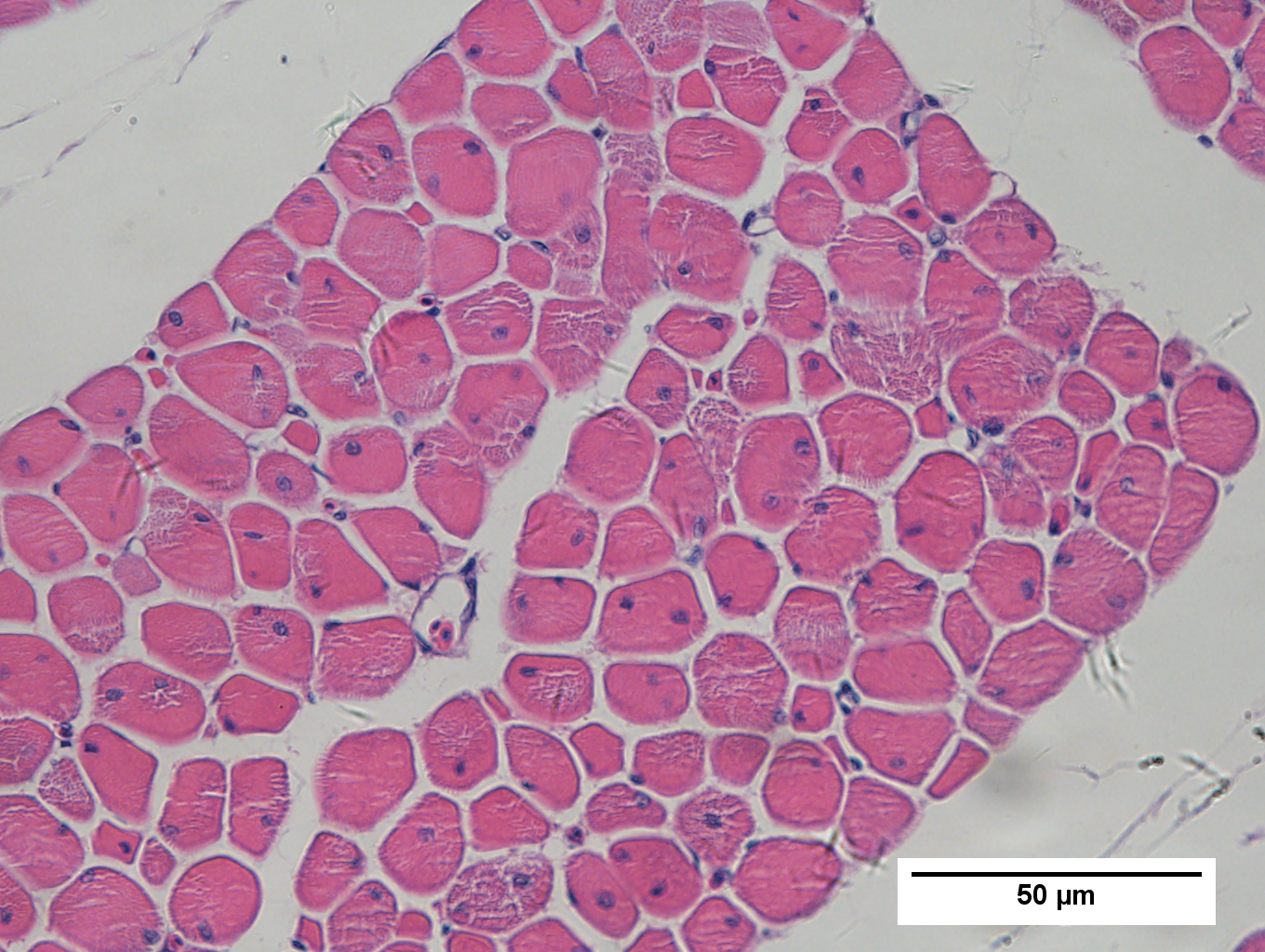

Supplement: S4 Data — (ZIP) [file pgen.1010923.s020.zip › S4 Data. The original photos of muscle samples/Cross section of muscle samples/LV-ov-circMEF2A2.tif]

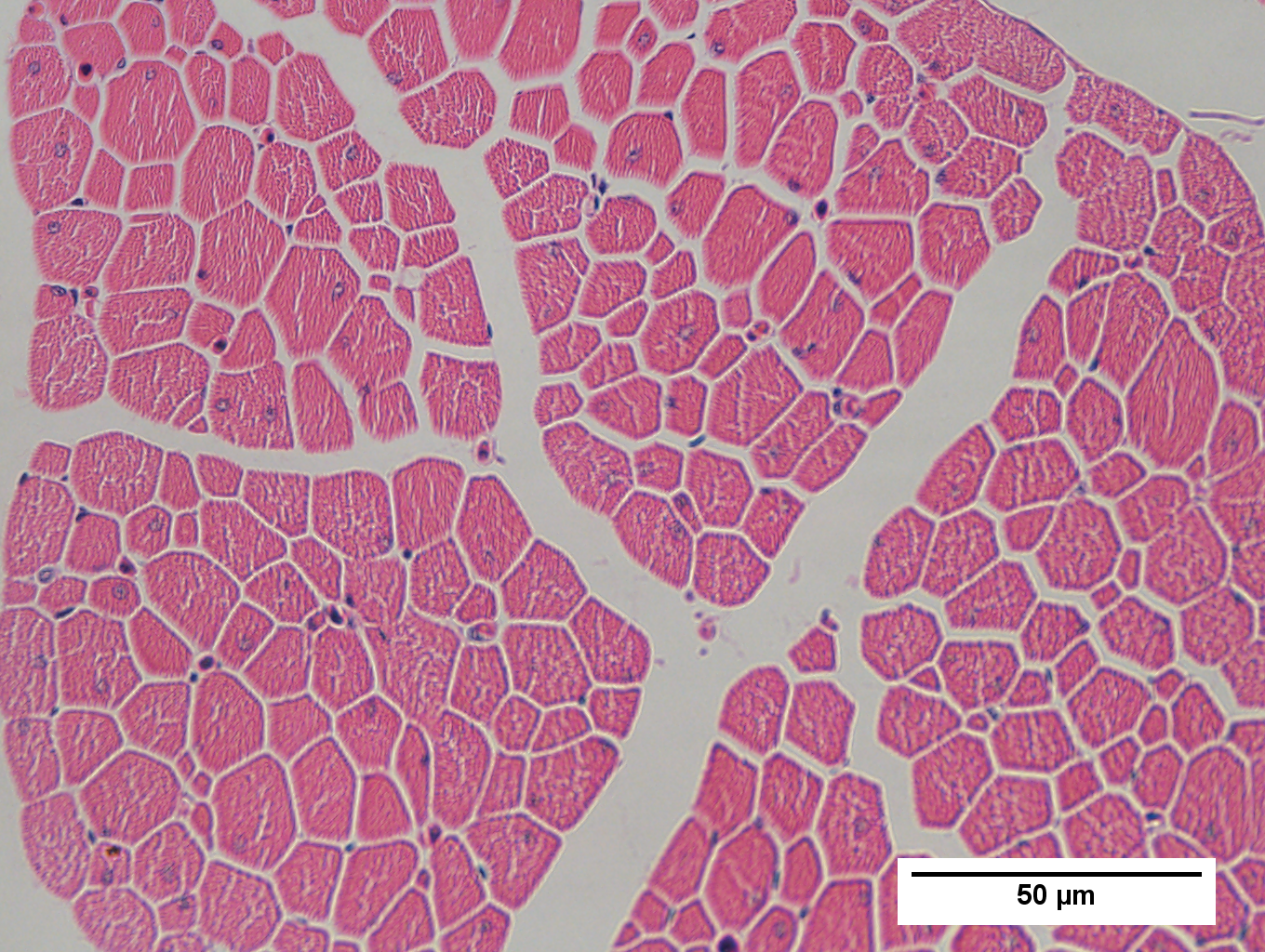

Supplement: S4 Data — (ZIP) [file pgen.1010923.s020.zip › S4 Data. The original photos of muscle samples/Cross section of muscle samples/LV-si-circMEF2A1-NC.tif]

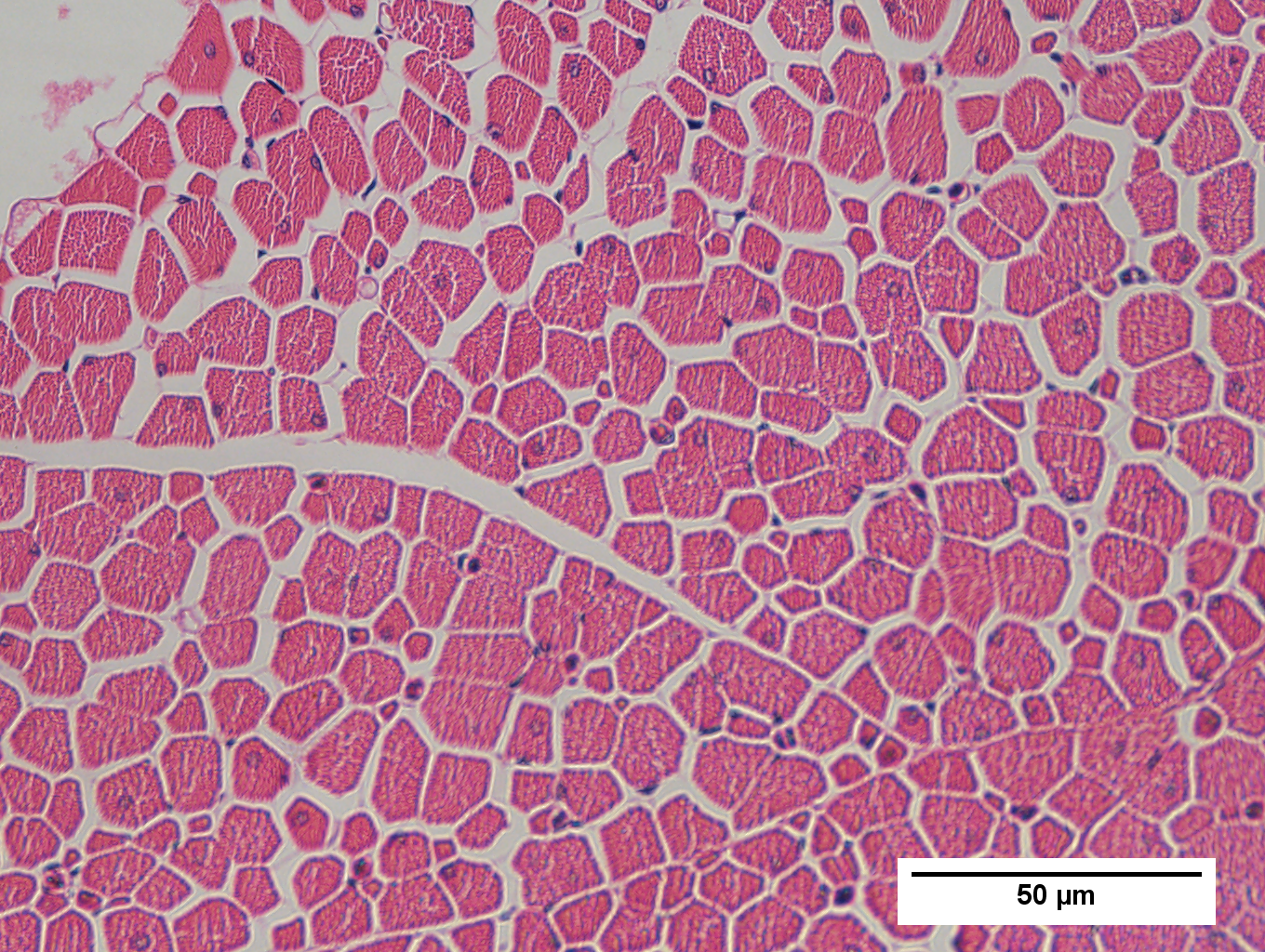

Supplement: S4 Data — (ZIP) [file pgen.1010923.s020.zip › S4 Data. The original photos of muscle samples/Cross section of muscle samples/LV-si-circMEF2A1.tif]

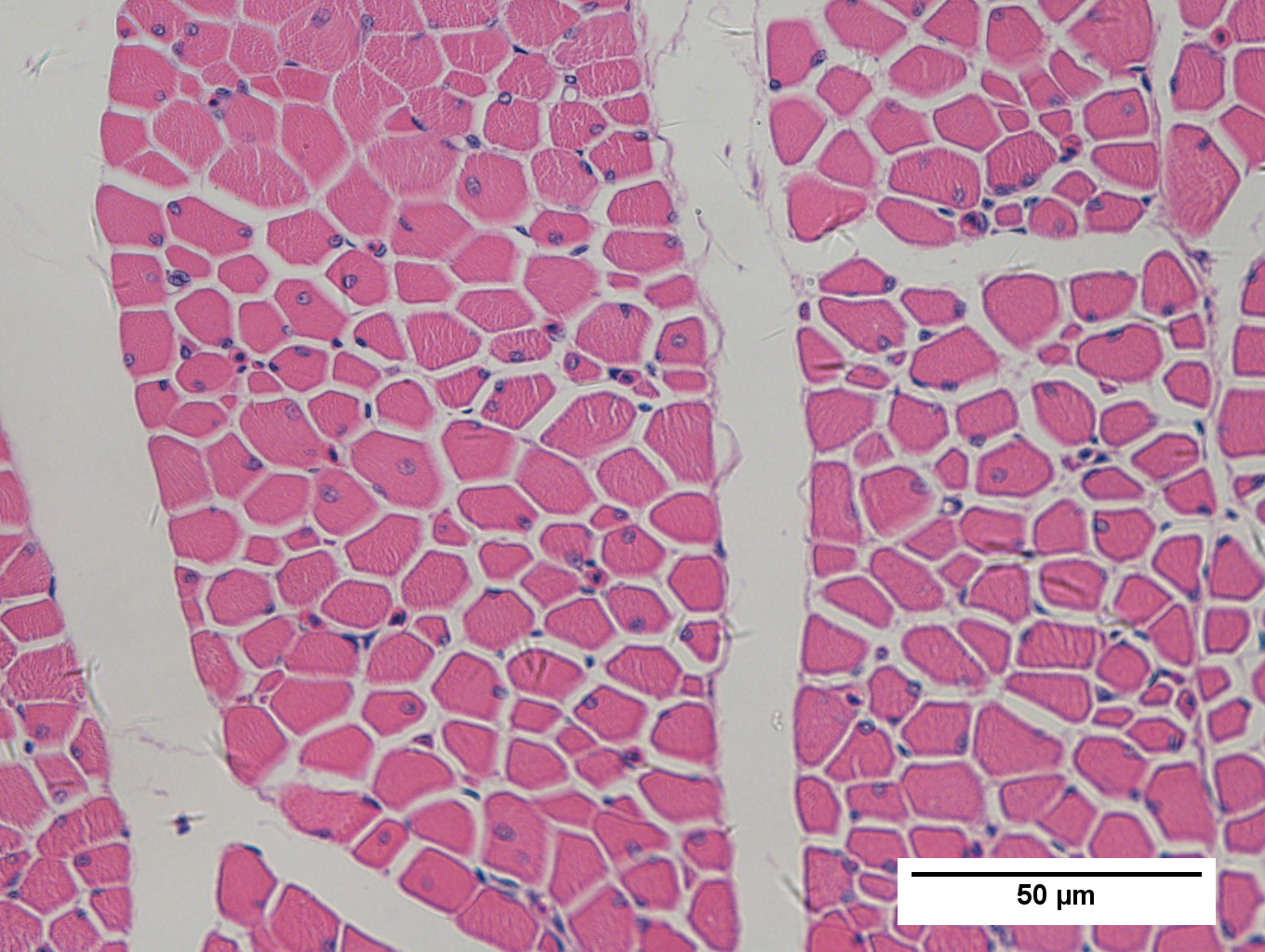

Supplement: S4 Data — (ZIP) [file pgen.1010923.s020.zip › S4 Data. The original photos of muscle samples/Cross section of muscle samples/LV-si-circMEF2A2-NC.tif]

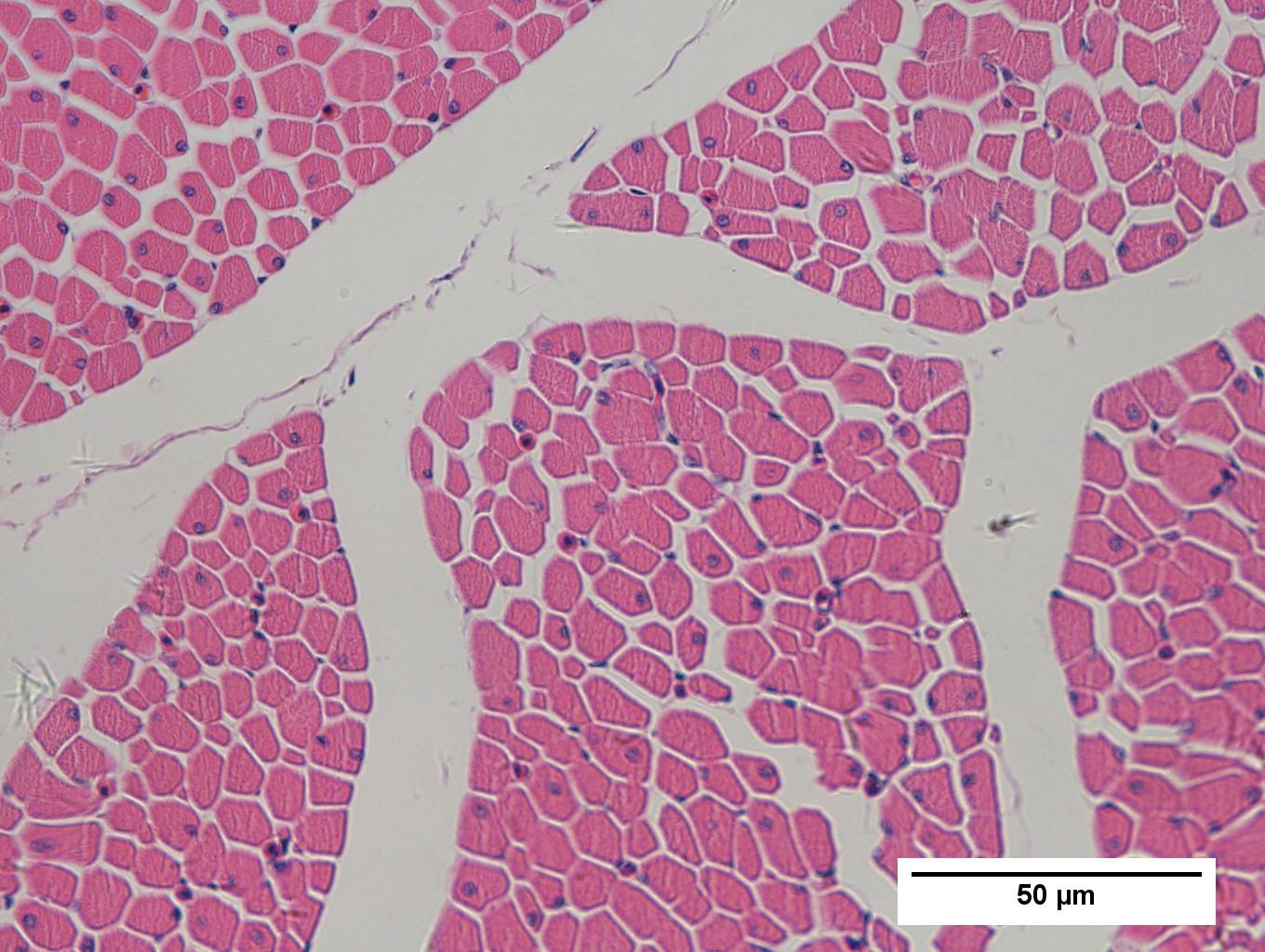

Supplement: S4 Data — (ZIP) [file pgen.1010923.s020.zip › S4 Data. The original photos of muscle samples/Cross section of muscle samples/LV-si-circMEF2A2.tif]

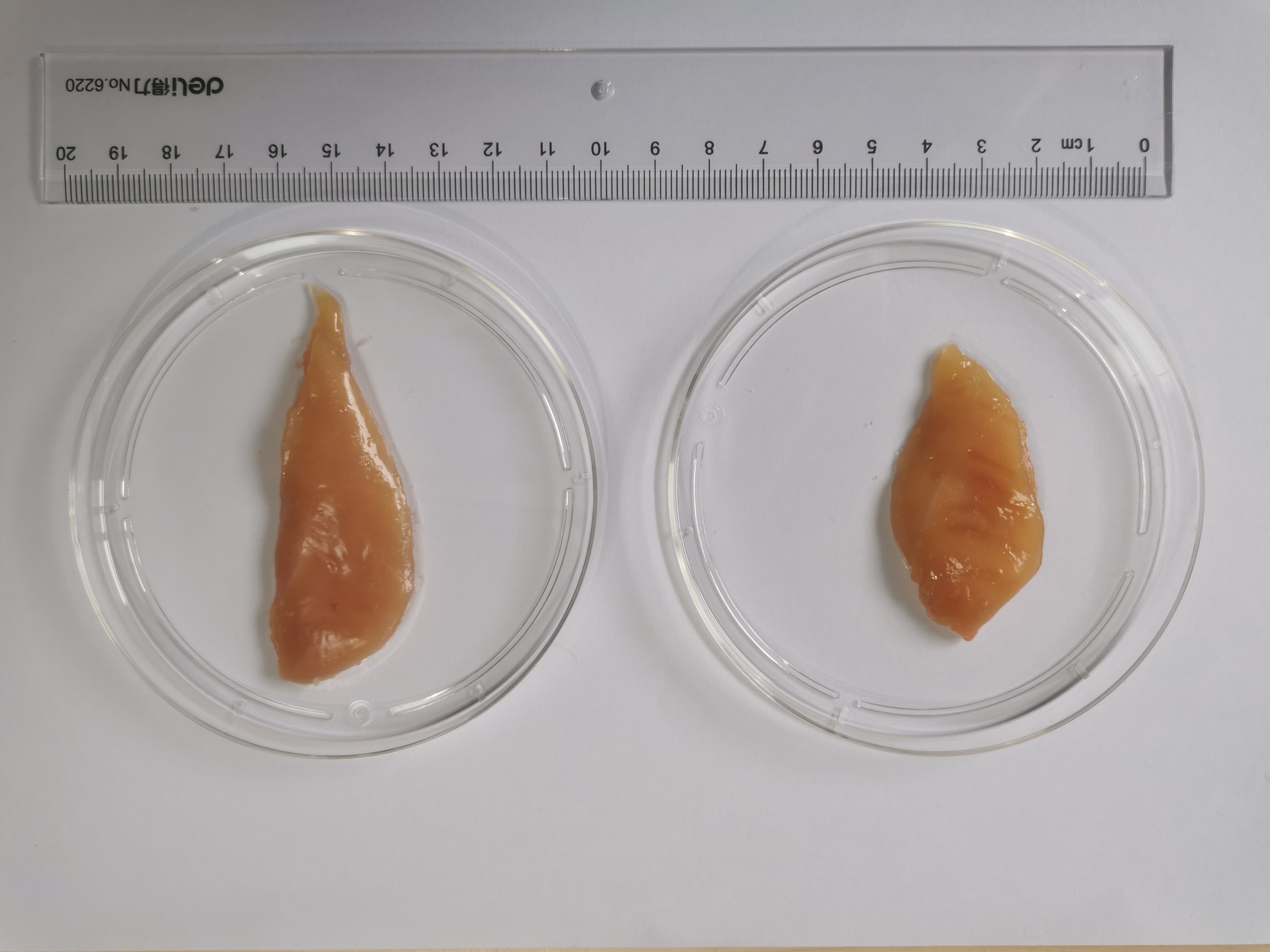

Supplement: S4 Data — (ZIP) [file pgen.1010923.s020.zip › S4 Data. The original photos of muscle samples/Muscle sample morphology/LV-ov-NC vs LV-ov-circMEF2A1.jpg]

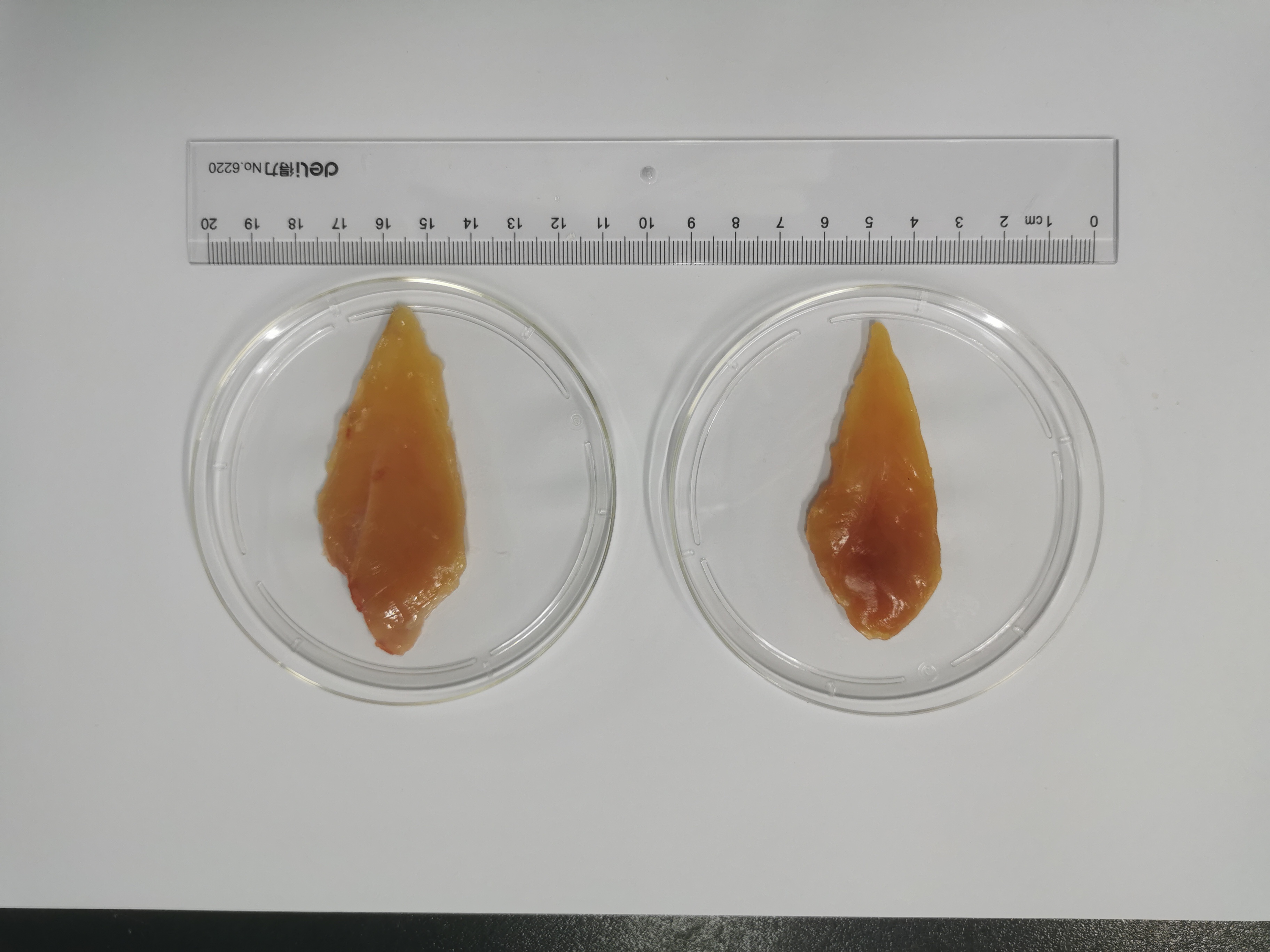

Supplement: S4 Data — (ZIP) [file pgen.1010923.s020.zip › S4 Data. The original photos of muscle samples/Muscle sample morphology/LV-ov-NC vs LV-ov-circMEF2A2.jpg]

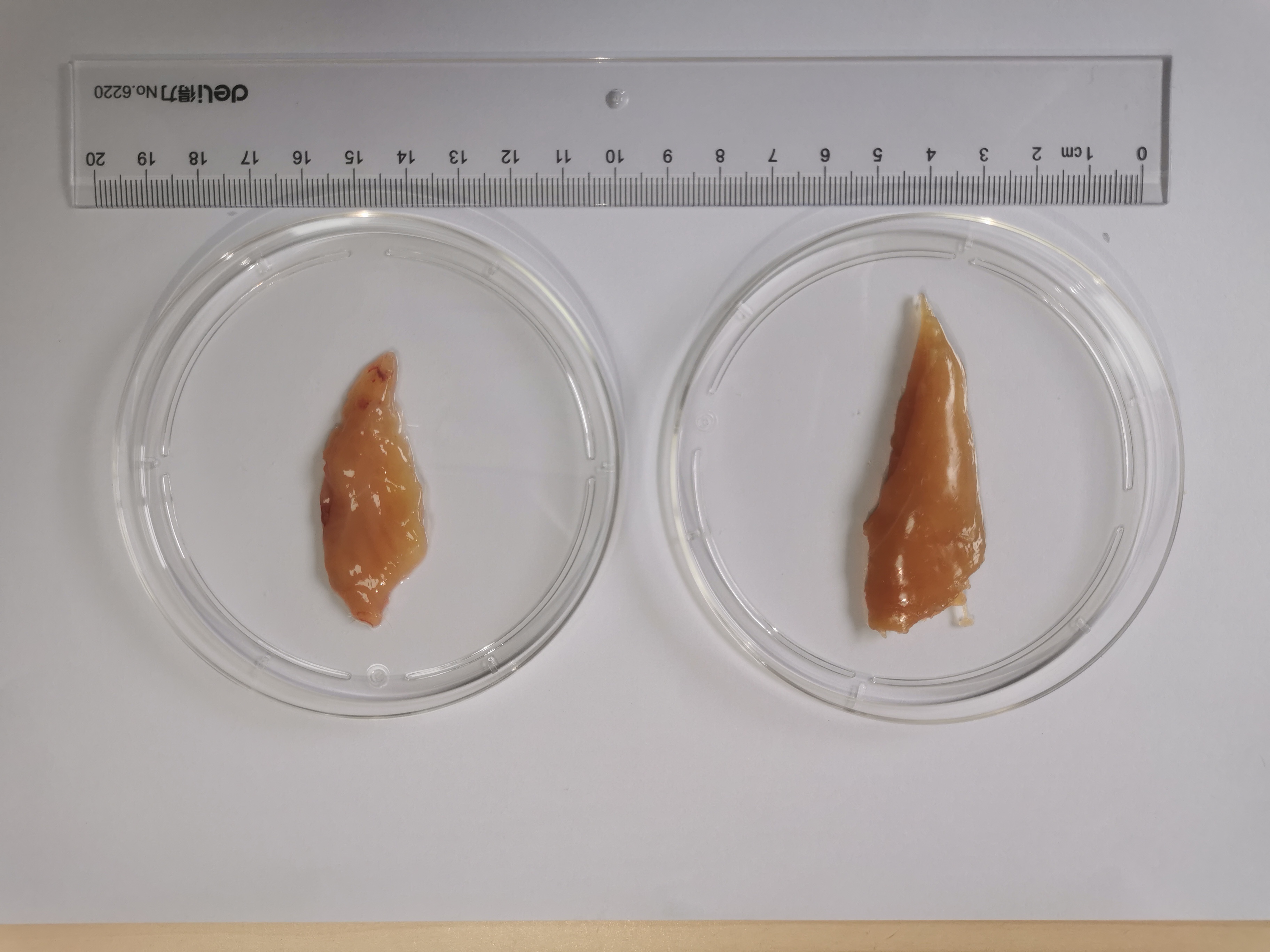

Supplement: S4 Data — (ZIP) [file pgen.1010923.s020.zip › S4 Data. The original photos of muscle samples/Muscle sample morphology/LV-si-NC vs LV-si-circMEF2A1.jpg]

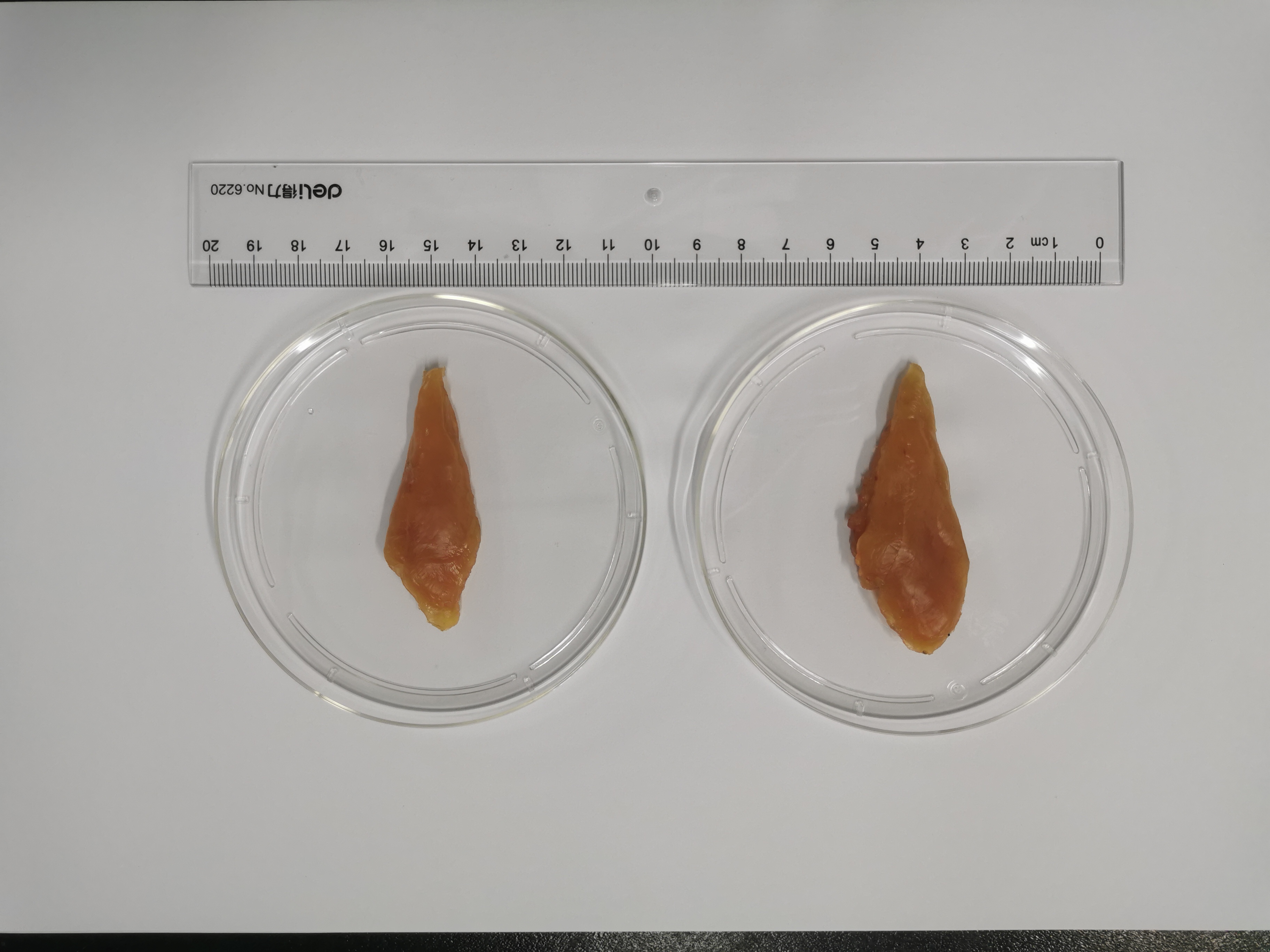

Supplement: S4 Data — (ZIP) [file pgen.1010923.s020.zip › S4 Data. The original photos of muscle samples/Muscle sample morphology/LV-si-NC vs LV-si-circMEF2A2.jpg]
